# VANGL2 alleviates inflammatory bowel disease by recruiting the ubiquitin ligase MARCH8 to limit NLRP3 inflammasome activation through OPTN-mediated selective autophagy

**DOI:** 10.1371/journal.pbio.3002961

**Published:** 2025-02-03

**Authors:** Huaji Jiang, Yingchao Xie, Zhiqiang Hu, Jiansen Lu, Jiahuan Zhang, Hongyu Li, Ke Zeng, Wenqiang Peng, Cheng Yang, Junsheng Huang, Zelong Han, Xiaochun Bai, Xiao Yu

**Affiliations:** 1 Department of Immunology, School of Basic Medical Sciences, Southern Medical University, Guangzhou, Guangdong, China; 2 Department of Orthopaedics, Yue Bei People’s Hospital Affiliated to Shantou University Medical College, Shaoguan, Guangdong, China; 3 Zhejiang Provincial Key Laboratory of Pancreatic Disease, The First Affiliated Hospital, Institute of Translational Medicine, Zhejiang University School of Medicine, Zhejiang University, Hangzhou, China; 4 Department of Joint Surgery, the Fifth Affiliated Hospital of Southern Medical University, Guangzhou, Guangdong, China; 5 Guangdong Provincial Key Laboratory of Bone and Joint Degeneration Diseases, Department of Cell Biology, School of Basic Medical Sciences, Southern Medical University, Guangzhou, Guangdong, China; 6 Department of Clinical Laboratory Medicine, Guangdong Provincial People’s Hospital (Guangdong Academy of Medical Sciences), Southern Medical University, Guangzhou, Guangdong, China; 7 First School of Clinic Medicine, Guangzhou University of Chinese Medicine, Guangzhou, China; 8 Youth Medical Association of Macao, Macao, China; 9 Guangdong Provincial Key Laboratory of Gastroenterology, Department of Gastroenterology, Nanfang Hospital, Southern Medical University, Guangzhou, Guangdong, China; University of Pennsylvania, UNITED STATES OF AMERICA

## Abstract

Inflammatory bowel disease (IBD) is a chronic and potentially life-threatening inflammatory disease of gastroenteric tissue characterized by episodes of intestinal inflammation, but the underlying mechanisms remain elusive. Here, we explore the role and precise mechanism of Van-Gogh-like 2 (VANGL2) during the pathogenesis of IBD. VANGL2 decreases in IBD patients and dextran sulfate sodium (DSS)-induced colitis in mice. Myeloid VANGL2 deficiency exacerbates the progression of DSS-induced colitis in mice and specifically enhances the activation of NLRP3 inflammasome in macrophages. NLRP3-specific inhibitor MCC950 effectively alleviates DSS-induced colitis in VANGL2 deficient mice. Mechanistically, VANGL2 interacts with NLRP3 and promotes the autophagic degradation of NLRP3 through enhancing the K27-linked polyubiquitination at lysine 823 of NLRP3 by recruiting E3 ligase MARCH8, leading to optineurin (OPTN)-mediated selective autophagy. Notably, decreased VANGL2 in the peripheral blood mononuclear cells from IBD patients results in overt NLRP3 inflammasome activation and sustained inflammation. Taken together, this study demonstrates that VANGL2 acts as a repressor of IBD progression by inhibiting NLRP3 inflammasome activation and provides insights into the crosstalk between inflammation and autophagy in preventing IBD.

## Abstract 2

Although NLRP3 inflammasome activation and impaired autophagy are linked to inflammatory bowel disease (IBD), the underlying mechanisms remain unclear. This study shows that VANGL2 reduces IBD progression by recruiting the ubiquitin ligase MARCH8 to regulate NLRP3 activation through OPTN-mediated autophagy.

## Introduction

Inflammatory bowel disease (IBD) is idiopathic intestinal chronic inflammation characterized by autoimmune dysfunction, including Crohn’s disease (CD) and ulcerative colitis (UC) [1]. IBD is mainly caused by the disorder of intestinal immune system under the action of environmental and genetic factors [2], but the mechanisms underlying remain elusive. IL-1β is a vital pro-inflammatory factor in the inflammatory cascade of IBD that impedes the integrity of the intestinal epithelial barrier [3]. The NLR family pyrin domain containing 3 (NLRP3) inflammasome is a multiprotein complex that includes NLRP3, apoptosis-associated speck-like protein containing a CARD (ASC) and Caspase-1 (Casp1) [4]. Once activated, NLRP3 inflammasome triggers proteolysis to cleavage into active Casp1 and catalyzes the conversion of pro-IL-1β to active IL-1β [4]. The aberrant activation of NLRP3 inflammasome is highly correlated with exacerbation of IBD [5,6], and 15 single-nucleotide polymorphisms in the NLRP3 gene region are associated with susceptibility to CD [7,8]. Upon activation, NLRP3 undergoes various posttranslational modifications (PTMs), including ubiquitination, phosphorylation, palmitoylation, SUMOylation, and acetylation [9–12]. The dynamic balance between ubiquitination and deubiquitination dynamically contributes to the stability of NLRP3 [10]. Negative regulators like E3 ubiquitin ligases TRIM31, MARCHF7, RNF125, and CPL-B are known to cause the K48/K63-linked polyubiquitination of NLRP3 and its degradation [13–15]. In contrast, deubiquitinating enzymes ABRO1 and BRCC3 cleave the ubiquitin chain of NLRP3 and maintain its stability [16,17]. However, the specific molecular mechanism regulating the activation of NLRP3 inflammasome during IBD progression has not been fully elucidated.

Autophagy is a critical intracellular degradation pathway that decomposes cytoplasmic components, organelles, and invading pathogens, and supports intracellular homeostasis, nutrient circulation, and stress response [18]. Autophagy can be highly selective by transporting protein aggregates and damaged or redundant organelles to the autophagosome through several cargo receptors [19]. Abnormal autophagy is implicated in the pathogenesis of IBD [8,20], and its modulators are important therapeutic agents for alleviating IBD [8,20,21]. In addition, autophagy is also involved in regulating NLRP3 inflammasome activation [22,23]. Chaperone-mediated autophagy has been reported to promote the degradation of NLRP3 [12], whereas cargo receptors p62 and CCDC50 have been identified to mediate the selective autophagic degradation of NLRP3 [20,22]. However, whether other types of autophagy and regulators are involved in moderating NLRP3 stability during IBD progression remains unclear.

Van-Gogh-like 2 (VANGL2) is a core protein of the planar cellular polarity (PCP) pathway, which is highly conserved among species [24,25]. VANGL2 encodes a transmembrane protein containing an N-terminal cytoplasmic region harbors a serine cluster motif, and the C-terminal cytoplasmic tail region encompasses a PDZ-binding domain that is implicated in intracellular signal transduction and interaction with other intracellular signal molecules [25,26]. VANGL2 has been reported to play an important role in coordinating intestinal epithelial morphogenesis, lumen formation, and intestinal tube elongation [27]. In addition, VANGL2 regulates the aggregation of mesenchymal cells and the formation of villi during intestinal development [28]. Despite the close association between VANGL2 and IBD [29], its role and mechanism in IBD progression is unclear.

Here, we demonstrate the role and regulatory mechanism of VANGL2 in IBD patients and DSS-induced colitis in mice. Myeloid *Vangl2*-deficiency in mice results in increased susceptibility to DSS-induced colitis due to the overactivation of NLRP3 inflammasome. In mechanism, VANGL2 interacts with NLRP3 and promotes the K27-linked ubiquitination of NLRP3 at lysine 823 by recruiting the E3 ubiquitin ligase MARCH8, thereby accelerating its selective autophagic degradation mediated by Optineurin (OPTN) and ultimately represses NLRP3 activation. Notably, knockdown of VANGL2 or MARCH8 effectively boosts NLRP3 inflammasome activation in healthy PBMCs, and decreased VANGL2 results in overt NLRP3 inflammasome activation in the PBMCs of IBD patients. These findings suggest that VANGL2 plays a negative role in NLRP3 inflammasome-driven inflammation of DSS-induced colitis, offering a new therapeutic approach for the treatment of IBD.

## Results

### Myeloid depletion of VANGL2 exacerbates intestinal inflammation in DSS-induced colitis in mice

**Fig 1:**
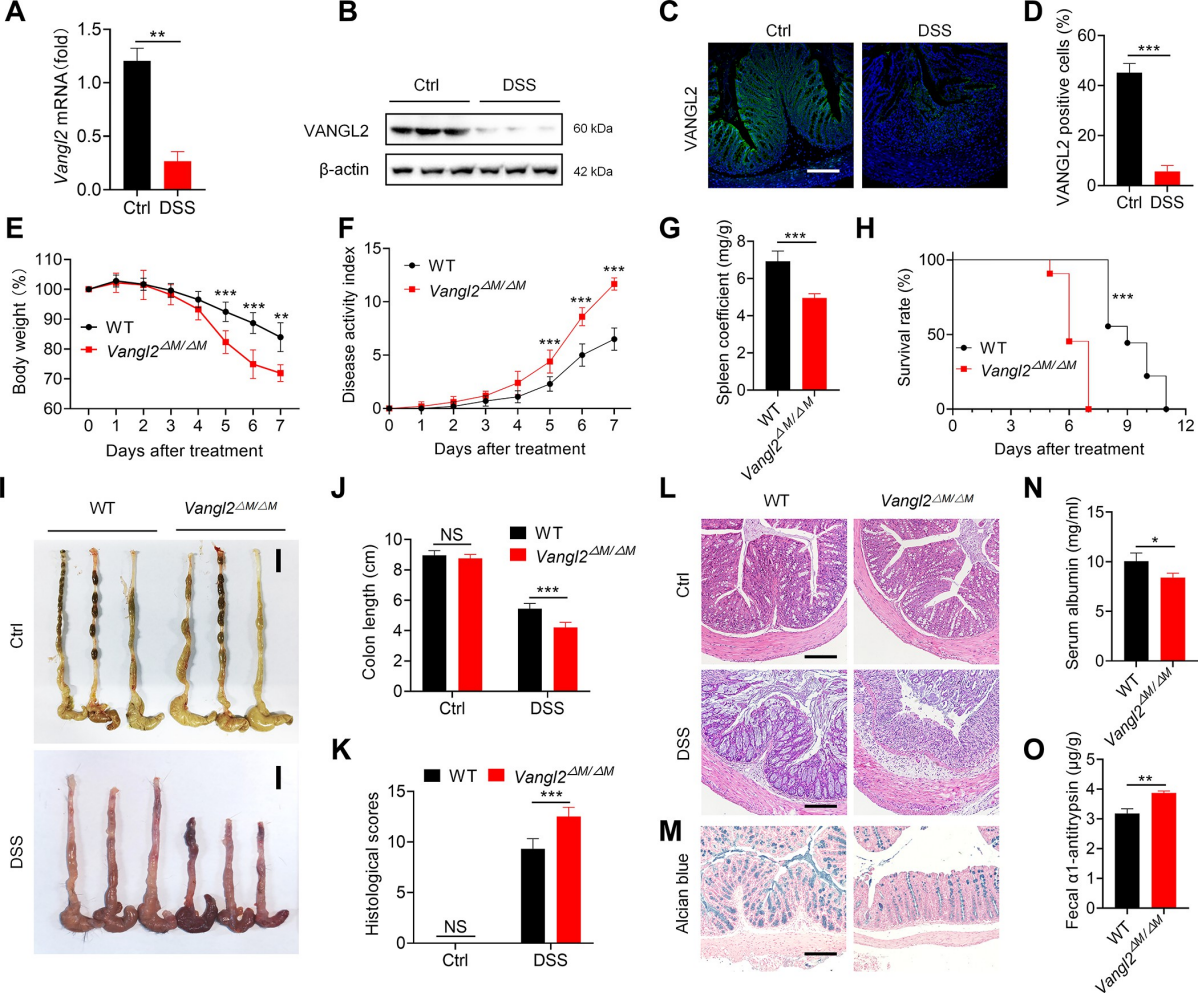
Myeloid knockout *VANGL2* exacerbates the progression of DSS-induced colitis in mice. (A) The mRNA expression of *Vangl2* in colon was detected by qRT-PCR on day 7 after DSS treatment (*n* = 8). (B) The protein expression of VANGL2 in colon was detected by immunoblot analysis on day 7 after DSS treatment. (C) Immunofluorescence staining was used to detect the expression of VANGL2 in colon on day 7 after DSS treatment (*n* = 3). Scale bar = 100 μm. (D) Statistical analysis of VANGL2 positive cells for (C). (E–H) The body weight change (E), disease activity index (F), spleen index (G), and survival rate (H) of WT and *Vangl2*^ΔM/ΔM^ mice after DSS treatment (*n* = 10). (I, J) Gross histological morphology (I) and intestinal length (J) were detected in WT and *Vangl2*^ΔM/ΔM^ mice with or without DSS treatment for 7 days (*n* = 12). Scale bar = 1 cm. (K, L) HE staining was performed in WT and *Vangl2*^ΔM/ΔM^ mice with or without DSS treatment for 7 days. Scale bar = 100 μm. (M) Alcian blue staining was performed in WT and *Vangl2*^ΔM/ΔM^ mice with DSS treatment for 7 days. Scale bar = 100 μm. (N, O) Intestinal permeability was determined by albumin level of serum (N) and fecal α1-antitrypsin level (O) in WT and *Vangl2*^ΔM/ΔM^ mice on day 7 after the DSS treatment (*n* = 3). Data are expressed as means ± SD. *P < 0.05, **P < 0.01, ***P < 0.001, NS means not significant. The data underlying this figure can be found in S1 Data and S1 Raw Images. DSS, dextran sulfate sodium; VANGL2, Van-Gogh-like 2; WT, wild-type.

We first assessed the expression of VANGL2 during IBD progression. Database biogenic analysis indicated a decrease in *VANGL2* mRNA expression in both human CD and UC (S1A Fig), suggesting that VANGL2 is down-regulated during IBD. Subsequently, we established a DSS-induced acute colitis model in C57BL/6 wild-type (WT) mice and traced the change of VANGL2 expression. We found that the mRNA level (Fig 1A) and protein level (Figs 1B and S1B) of VANGL2 in colon were decreased in mice with DSS-induced colitis. Similarly, immunofluorescence staining analysis also showed a reduction in VANGL2 protein levels in the colon (Fig 1C and 1D). These results demonstrate that VANGL2 is down-regulated in both human IBD and DSS-induced colitis in mice.

We recently explored the function of VANGL2 in anti-viral infection and sepsis [18,30]. To determine the role of VANGL2 in IBD and whether key innate immune pathways are involved, we generated myeloid conditional knockout of *Vangl2* (*Vangl2*^ΔM/ΔM^) by breeding *Vangl2*^flox/flox^ mice with *Lyz2*-Cre mice (S1C Fig), and the knockout efficiency of VANGL2 protein in myeloid cells was verified by immunoblot analysis (S1D Fig). We challenged WT and *Vangl2*^ΔM/ΔM^ mice with 2.5% DSS to induce acute colitis and found that *Vangl2*^ΔM/ΔM^ mice were highly susceptible to DSS-induced colitis, showed more dramatic weight loss (Fig 1E), higher clinical disease scores (Fig 1F), and lower spleen coefficient (Fig 1G), compared to WT mice. Consistently, DSS-treated *Vangl2*^ΔM/ΔM^ mice died earlier (Fig 1H) with significantly shortened colons (Fig 1I and 1J) than WT mice. Meanwhile, histological staining showed more intense intestinal inflammatory infiltration, reduced goblet cells, and increased histological scores in *Vangl2*^ΔM/ΔM^ mice (Fig 1K–1M). In addition, *Vangl2*^ΔM/ΔM^ colitis mice exhibited lower levels of serum albumin (Fig 1N) and higher levels of fecal α1-antitrypsin (Fig 1O) than WT mice. Therefore, these results suggest that myeloid knockout of VANGL2 causes excessive inflammation and exacerbates the progression of DSS-induced colitis in mice.

Previous studies have shown that miR-335 can directly target VANGL2 and inhibit its expression [31]. Moreover, miR-335 expression is up-regulated during IBD [32]. Consistent with previous study, our data demonstrated that miR-335 is up-regulated during IBD and DSS-induced colitis (S1E and S1F Fig). Also, miR-335 effectively suppressed the expression of VANGL2 (S1G Fig). Furthermore, we examined the expression of VANGL2 in both active IBD and inactive IBD patients. The data showed that the *VANGL2* expression of active IBD patients was significantly lower than that of inactive IBD patients (S1H Fig). Therefore, these results suggest that the down-regulation of VANGL2 in IBD patients might be caused by the elevated miR-335.

### VANGL2 specifically inhibits the activation of the NLRP3 inflammasome

**Fig 2:**
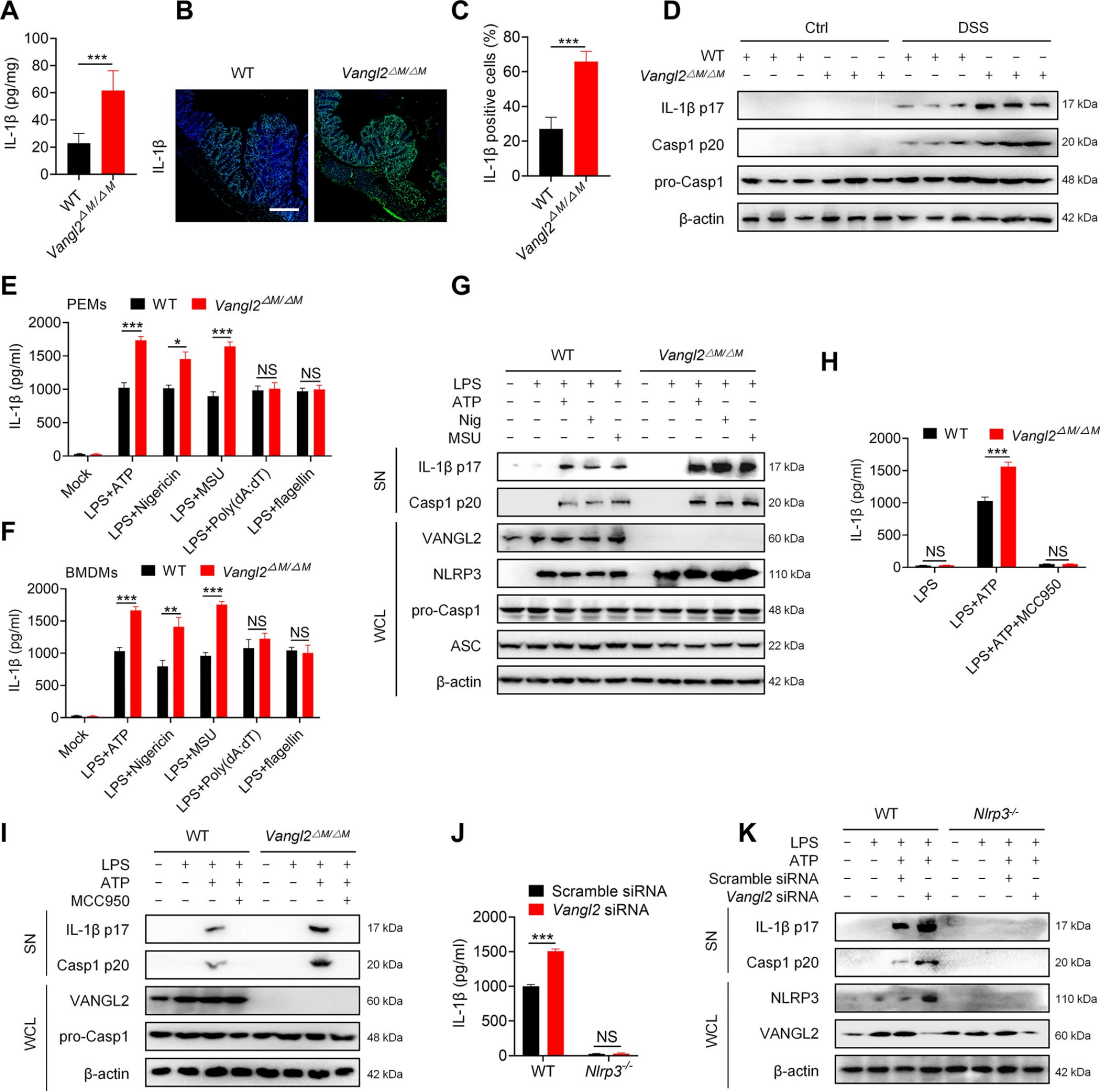
VANGL2 specifically inhibits the activation of NLRP3 inflammasome. (A) The expression of IL-1β p17 in intestinal supernatant (SN) of WT and *Vangl2*^ΔM/ΔM^ mice with DSS-induced colitis was detected by ELISA (*n* = 8). (B) The expression of IL-1β p17 in WT and *Vangl2*^ΔM/ΔM^ mice with DSS-induced colitis was detected by immunofluorescence staining. Scale bar = 100 μm. (C) Statistical analysis of IL-1β positive cells for (B) (*n* = 6). (D) Immunoblot analysis was used to detect the expression of intestinal inflammasome-related proteins (IL-1β p17, Casp1 p20, and pro-Casp1) in WT and *Vangl2*^ΔM/ΔM^ mice with DSS-induced colitis. (E) LPS-pretreated PEMs (WT and *Vangl2*^ΔM/ΔM^) were stimulated with different inflammasome-related agonists for indicated times. The expression of IL-1β p17 in the supernatant (SN) was detected by ELISA. (F) LPS-pretreated BMDMs (WT and *Vangl2*^ΔM/ΔM^) were stimulated with different inflammasomes-related agonists for indicated times, and the expression of IL-1β p17 in the cell SN was detected by ELISA. (G) LPS-pretreated PEMs were stimulated with different NLRP3 inflammasome agonists for indicated times. Immunoblot analysis was used to detect the expression of SN proteins (IL-1β p17 and Casp1 p20) and WCL proteins (VANGL2, NLRP3, pro-Casp1, and ASC). (H) LPS-primed PEMs (WT and *Vangl2*^ΔM/ΔM^) were pretreated with MCC950 (1 μM) for 2 h, followed by the treatment with ATP (5 mM) for 30 min, and the expression of IL-1β p17 in the SN was detected by ELISA. (I) LPS-primed PEMs (WT and *Vangl2*^ΔM/ΔM^) were pretreated with MCC950 (1 μM) for 2 h, followed by the treatment with ATP (5 mM) for 30 min, and the expressions of IL-1β p17, Casp1 p20, pro-Casp1, and VANGL2 were detected by immunoblot analysis. (J) PEMs (WT and *Nlrp3*^*-/-*^) were silenced by *Vangl2* siRNA for 24 h, followed by LPS and ATP treatment. The expression of IL-1β was detected by ELISA. (K) PEMs (WT and *Nlrp3*^*-/-*^) were silenced by *Vangl2* siRNA for 24 h, followed by LPS and ATP treatment, and the expressions of IL-1β p17, Casp1 p20, NLRP3, and VANGL2 were detected by immunoblot analysis. Data are expressed as means ± SD. *P < 0.05, **P < 0.01, ***P < 0.001, NS means not significant. The data underlying this figure can be found in S1 Data and S1 Raw Images. BMDM, bone marrow-derived macrophage; PEM, peritoneal macrophage; VANGL2, Van-Gogh-like 2; WCL, whole cell lysate; WT, wild-type.

Inflammasome activation and release of IL-1β is known to be an important pathological factor that accelerates the progression of DSS-induced colitis [6]. We next investigated whether VANGL2 affects inflammasome activation in mice with DSS-induced colitis. Our results of immunofluorescence staining and ELISA indicated that myeloid knockout of *Vangl2* promoted the release of IL-1β in the colon supernatant (Fig 2A) and the expression of IL-1β in colon (Fig 2B and 2C), which were also verified by increased cleavage of IL-1β and Caspase-1 in *Vangl2*^ΔM/ΔM^ mice (Fig 2D). Therefore, VANGL2 negatively regulates the inflammasome activation in mice with DSS-induced colitis.

To clarify which specific inflammasome was suppressed by VANGL2, we silenced *Vangl2* in primary mouse macrophages by using small interfering RNA (siRNA) (S2A Fig). ELISA results showed that silencing *Vangl2* in peritoneal macrophages (PEMs) significantly promoted the release of IL-1β induced by ATP, Nigericin, and MSU (S2B Fig), but not by genomic DNA (gDNA), Poly(dA:dT), or flagellin (S2B Fig), indicated that VANGL2 specifically inhibits the NLRP3 inflammasome activation, but did not affect the activation of AIM2 or NLRC4 inflammasome. Consistent results were also obtained in bone marrow-derived macrophages (BMDMs) (S2C Fig). In addition, immunoblot analysis further revealed that silencing of *Vangl2* substantially promoted the cleavage of IL-1β and Caspase-1 induced by ATP, Nigericin, and MSU (S2D Fig). Furthermore, VANGL2 did not affect the phosphorylation of p65 and IκBα in mice with DSS-induced colitis (S2E Fig). Moreover, VANGL2 cannot affect with the secretion of IL-6 and TNF-α in the colon (S2F and S2G Fig). These results demonstrate that VANGL2 has no significant effect on the priming signal of the NLRP3 inflammasome in DSS-induced colitis. Therefore, these results suggest that silencing VANGL2 promotes the NLRP3 inflammasome activation.

Furthermore, we verified these results in primary macrophages from WT and *Vangl2*^ΔM/ΔM^ mice. Compared with WT PEMs, the expression of IL-1β induced by ATP, Nigericin, and MSU was significantly enhanced in *Vangl2*^ΔM/ΔM^ PEMs and BMDMs (Fig 2E and 2F). In contrast, *Vangl2* deletion did not alter the release of IL-1β after Poly(dA:dT) or flagellin stimulation (Fig 2E and 2F). Moreover, immunoblot analysis showed that the absence of *Vangl2* in PEMs escalated the cleavages of IL-1β and Caspase-1 mediated by ATP, Nigericin, and MSU, while pro-Casp1 expression remained unaffected (Fig 2G). Therefore, these results indicate that VANGL2 effectively suppresses the activation of NLRP3 inflammasome.

To further investigate the specific impact of VANGL2 on NLRP3 inflammasome activation, we utilized the NLRP3-specific inhibitor MCC950 to pharmacologically inhibit NLRP3 activity. Our findings indicated that the absence of *Vangl2* no longer affected the expression of IL-1β and Casp1 in PEMs in the presence of MCC950 (Fig 2H and 2I). Notably, *Vangl2* knockdown did not impact the expression of IL-1β and Casp1 in *Nlrp3*^*-/-*^ PEMs (Fig 2J and 2K). Taken together, these data indicate that VANGL2 has a specific inhibitory effect on the activation of the NLRP3 inflammasome.

### VANGL2 interacts with NLRP3

**Fig 3:**
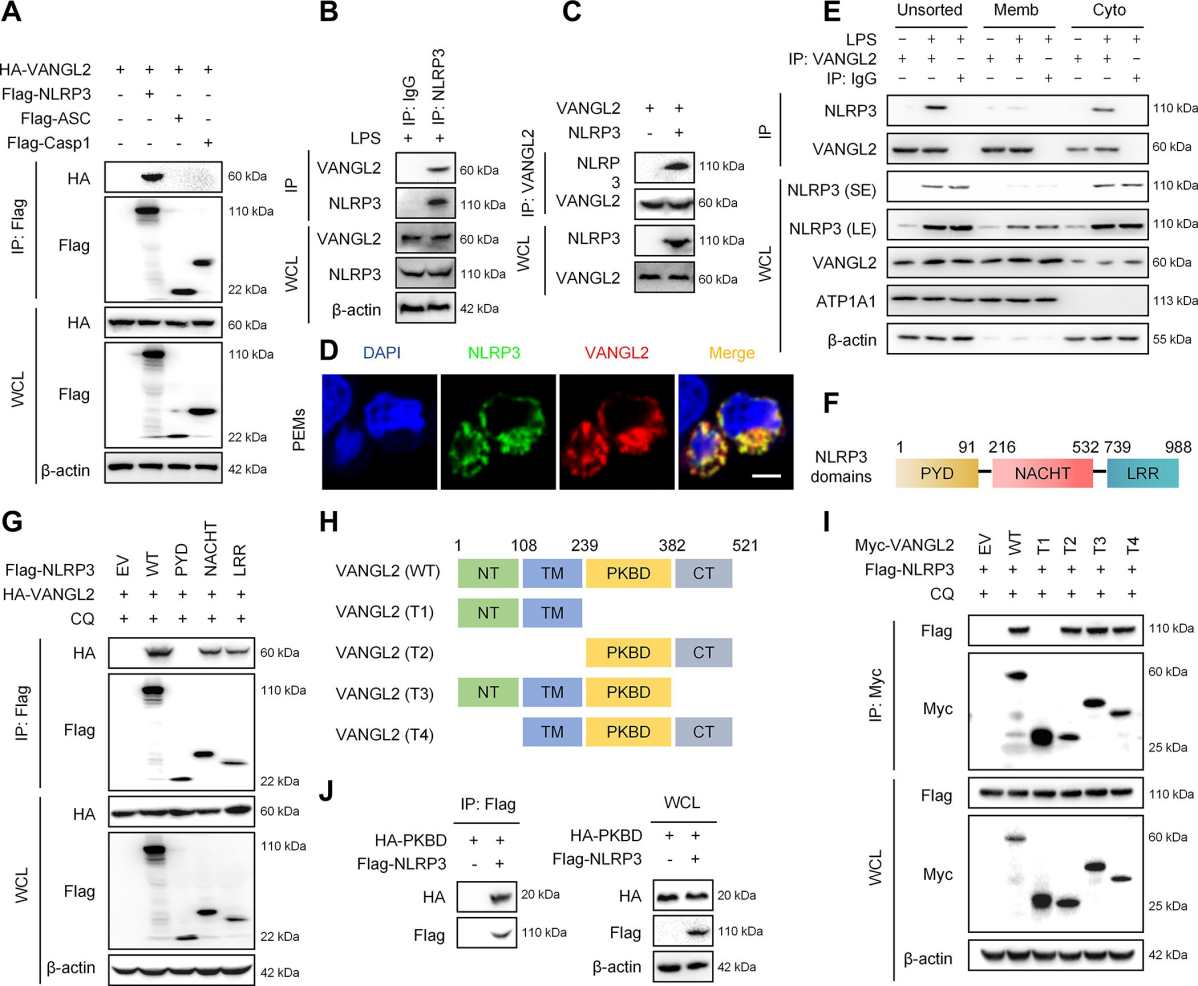
VANGL2 interacts with NLRP3. (A) HA-VANGL2, Flag-NLRP3, Flag-ASC, and Flag-Casp1 plasmids were transfected into HEK293T cells for 24 h. The expression of HA and Flag tagged proteins were detected by immunoblot analysis. (B) PEMs were pretreated with LPS (100 ng/ml) for 6 h, then NLRP3 was pulled down by IP assay, and the expressions of VANGL2 and NLRP3 were detected by immunoblot analysis. (C) Co-IP analysis of the interaction between purified VANGL2 and NLRP3 in vitro. Recombinant VANGL2 and NLRP3 proteins were incubated for 1 h, and then the mixture was subjected to IP with anti-VANGL2 antibody followed by immunoblot analysis. (D) PEMs were pretreated with LPS (100 ng/ml) for 6 h, and the co-localization of NLRP3 (green) and VANGL2 (red) was detected by immunofluorescence staining. Scale bar = 5 μm. (E) PEMs were pretreated with LPS (100 ng/ml) for 6 h, and cytoplasmic and cell membrane proteins were isolated by cellular fractionation. VANGL2 was pulled down by IP assay, and the expression of inflammasome-associated proteins were detected by immunoblot analysis. (F) Different domains of NLRP3 protein. (G) Truncations of Flag-NLRP3 and HA-VANGL2 plasmids were transfected into HEK293T cells for 24 h, and then the interaction between VANGL2 and NLRP3 was detected by Co-IP and immunoblot analysis. (H) Different domain segments of VANGL2. (I) Truncations of Myc-VANGL2 and Flag-NLRP3 plasmids were transfected into HEK293T cells for 24 h, and then the combination of VANGL2 and NLRP3 was detected by Co-IP and immunoblot analysis. (J) HA-PKBD (VANGL2) and Flag-NLRP3 plasmids were transfected into HEK293T cells for 24 h, and then the expressions of HA and Flag proteins were detected by Co-IP and immunoblot analysis. The data underlying this figure can be found in S1 Raw Images. PEM, peritoneal macrophage; VANGL2, Van-Gogh-like 2.

To explore the molecular mechanism by which VANGL2 affects NLRP3 inflammasome activation, we examined whether VANGL2 interacts with NLRP3 inflammasome components. We co-transfected the plasmids containing NLRP3, ASC, Casp1, and VANGL2 into HEK293T cells, and co-immunoprecipitation (co-IP) data showed that VANGL2 interacted with NLRP3 but not with ASC or Casp1 (Figs 3A and S3A). Similarly, endogenous co-IP assay also showed the interaction between VANGL2 and NLRP3 upon stimulation (Figs 3B and S3B). To further determine whether VANGL2 could directly bind to NLRP3, we performed an *in vitro* IP experiment and found that VANGL2 directly interacted with NLRP3 (Fig 3C). Next, confocal microscopy analysis was conducted to investigate the cellular localization of VANGL2-NLRP3 binding. Endogenous and exogenous co-localization experiments both showed that VANGL2 and NLRP3 co-located in the cytoplasm (Figs 3D and S3C). To further determine the subcellular localization of the VANGL2-NLRP3 complex, we isolated cytosolic and cytomembrane fractions and subjected to a co-IP assay. The results showed that VANGL2 and NLRP3 predominantly interacted in the cytoplasm (Figs 3E and S3D).

NLRP3 is a tripartite protein composed of an N-terminal pyrin (PYD), a central NACHT, and a C-terminal leucine-rich repeat (LRR) domain [4] (Fig 3F). To determine the crucial domain for the interaction between NLRP3 and VANGL2, we generated various truncates of NLRP3. Co-IP experiments showed that NLRP3 interacted with VANGL2 through its NACHT domain and LRR domain, but not the PYD domain (Fig 3G). Next, we proceed to explore which domains of VANGL2 interact with NLRP3. VANGL2 contains an N terminal domain (NT), a transmembrane domain (TM), a Prickle/cadherin-binding domain (PKBD), and a C terminal (CT) (Fig 3H). We constructed truncated VANGL2 plasmids (Fig 3H) and found only VANGL2 (T1) cannot interact with NLRP3, rather than other truncates (Figs 3I and S3E). Since all other NLRP3-interacting VANGL2 truncates contain the PKBD domain, we directly constructed the PKBD domain plasmid to determine whether this domain is critical for the interaction between VANGL2 and NLRP3. The Co-IP experiment data showed that the PKBD domain interacted with NLRP3 (Fig 3J). Therefore, these results suggest that VANGL2 interacts with the NACHT and LRR domains of NLRP3 through its PKBD domain.

### VANGL2 promotes OPTN-mediated selective autophagic degradation of NLRP3

**Fig 4:**
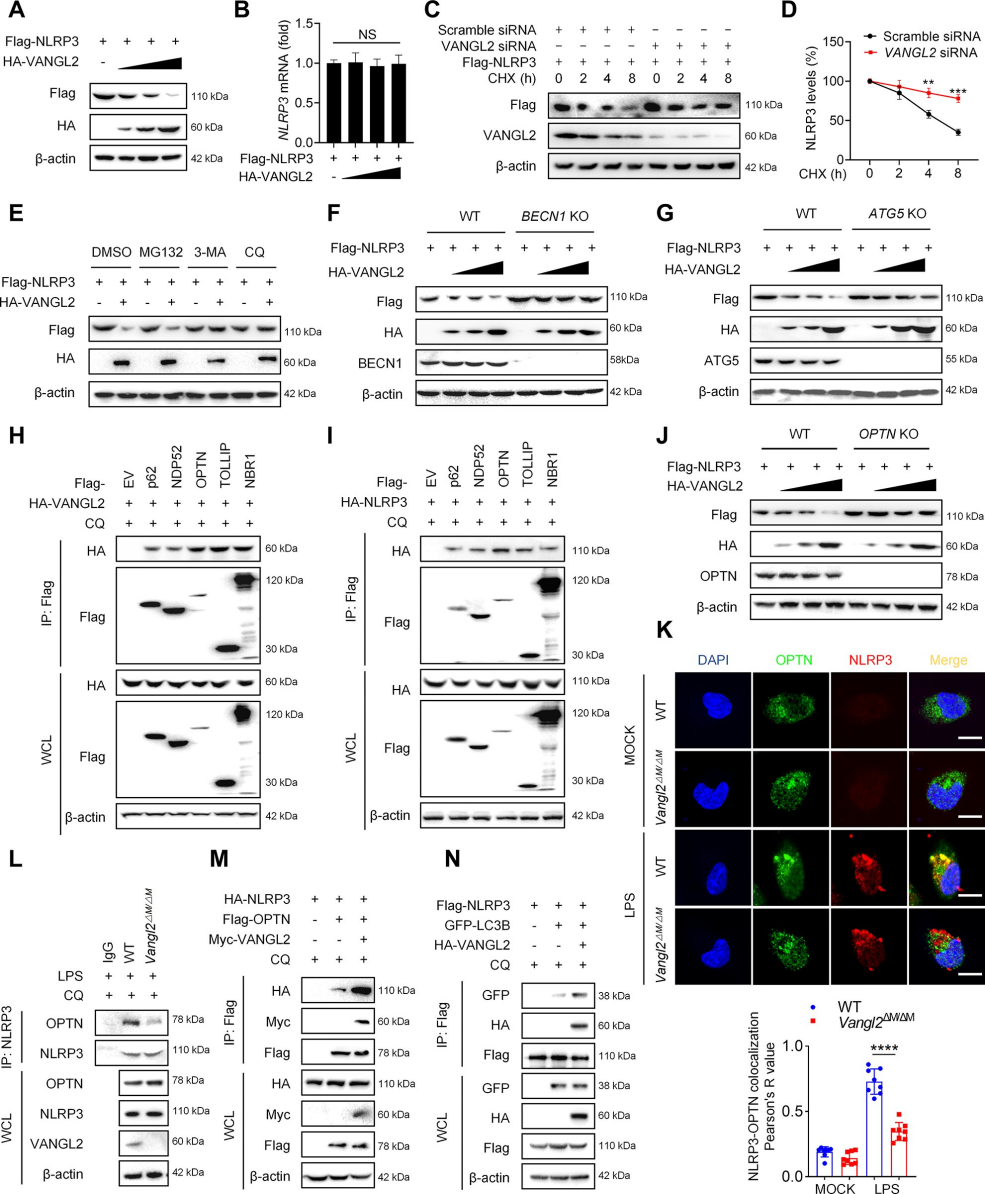
VANGL2 recruits NLRP3 to OPTN for selective autophagic degradation. (A, B) Flag-NLRP3 and HA-VANGL2 plasmids were transfected into HEK293T cells for 24 h. Immunoblot analysis was used to detect the protein expression of Flag-NLRP3 and HA-VANGL2 (A), and qRT-PCR was used to detect the mRNA expression of *NLRP3* (B). (C) HEK293T cells were silenced with *VANGL2* siRNA for 24 h, then transfected with Flag-NLRP3 plasmid for 24 h, and then treated with CHX (50 μg/ml) for indicated times. The expression of Flag-NLRP3 was detected by immunoblot analysis. (D) NLRP3 degradation rate for (C). (E) Flag-NLRP3 and HA-VANGL2 plasmids were transfected into HEK293T cells for 24 h, followed by treatment with MG132 (10 μM), 3-MA (10 mM), and CQ (50 μM) for 6 h. Immunoblot analysis was used to detect the protein expression of Flag-NLRP3 and HA-VANGL2. (F) Flag-NLRP3 and HA-VANGL2 plasmids were transfected into WT and *BECN1* KO HEK293T cells, and the expression of Flag-NLRP3, HA-VANGL2, and BECN1 was detected by immunoblot analysis. (G) Flag-NLRP3 and HA-VANGL2 plasmids were transfected into WT and *ATG5* KO HEK293T cells, and the protein expression of Flag-NLRP3, HA-VANGL2, and ATG5 were detected by immunoblot analysis. (H) Different cargo receptors and HA-VANGL2 plasmids were transfected into HEK293T cells for 24 h and then treated with CQ (50 μM) for 6 h. The expression of HA and Flag tagged proteins were detected by Co-IP and immunoblot analysis. (I) Different cargo receptors and HA-NLRP3 plasmids were transfected into HEK293T cells for 24 h and then treated with CQ (50 μM) for 6 h. The expression of HA and Flag tagged proteins were detected by Co-IP and immunoblot analysis. (J) Flag-NLRP3 and HA-VANGL2 plasmids were transfected into WT and *OPTN* KO HEK293T cells for 24 h, and the expression of Flag-NLRP3, HA-VANGL2, and OPTN were detected by immunoblot analysis. (K) PEMs (WT and *Vangl2*^ΔM/ΔM^) were stimulated by LPS (100 ng/ml) and CQ (50 μM) for 6 h, and the co-localization of OPTN and NLRP3 was detected by immunofluorescence staining. Scale bar = 5 μm. (L) WT and *Vangl2*^ΔM/ΔM^ PEMs were treated with LPS (100 ng/ml) and CQ (50 μM) for 6 h, and the expressions of OPTN, NLRP3, and VANGL2 were detected by Co-IP and immunoblot analysis. (M) HA-NLRP3, Flag-OPTN, and Myc-VANGL2 plasmids were transfected into HEK293T cells for 24 h, and then treated with CQ (50 μM) for 6 h. The expression of HA, Flag, and Myc tagged proteins were detected by Co-IP and immunoblot analysis. (N) Flag-NLRP3, GFP-LC3B, and HA-VANGL2 plasmids were transfected into HEK293T cells for 24 h, and then treated with CQ (50 μM) for 6 h. The expression of GFP, Flag, and HA tagged proteins were detected by Co-IP and immunoblot analysis. Data are expressed as means ± SD. **P < 0.01, ***P < 0.001, NS means not significant. The data underlying this figure can be found in S1 Data and S1 Raw Images. CHX, cycloheximide; CQ, chloroquine; PEM, peritoneal macrophage; VANGL2, Van-Gogh-like 2; WT, wild-type.

Next, we further explored the mechanism by which VANGL2 inhibits NLRP3 inflammasome activation. We first examined the effect of VANGL2 on the expression of NLRP3 inflammasome components. The results showed that overexpression of VANGL2 inhibited the protein expression of NLRP3 in a dose-dependent manner (Fig 4A), but did not affect the level of Casp1 and ASC (S4A and S4B Fig). Moreover, VANGL2 did not affect the transcription level of *NLRP3* (Fig 4B), suggesting that VANGL2 affects the posttranslational modification of the NLRP3 protein. To further investigate the potential impact of VANGL2 on NLRP3 protein degradation, we utilized the protein synthesis inhibitor cycloheximide (CHX) and found that knockdown of *VANGL2* delayed the degradation of NLRP3 in the presence of CHX (Fig 4C and 4D), indicating that VANGL2 plays a role in promoting NLRP3 degradation.

The protein degradation systems mainly include the proteasome pathway and the autophagy-lysosome pathway [33]. We investigated the VANGL2-mediated NLRP3 degradation pathway and discovered that both the autophagy inhibitor 3-methyladenine (3-MA) and the lysosome inhibitor chloroquine (CQ) prevented VANGL2-mediated NLRP3 degradation, whereas the proteasome inhibitor MG132 did not (Fig 4E). In addition, knockout of the autophagic gene *BECN1* or *ATG5* effectively blocked the degradation of NLRP3 induced by VANGL2 (Fig 4F and 4G). Furthermore, *Vangl2* knockout decelerated the autophagic degradation of NLRP3 induced by autophagy activator Earle’s balanced salt solution (EBSS) (S4C Fig). Therefore, these data suggest that VANGL2 promotes the degradation of NLRP3 through the autophagy-lysosome pathway.

Increasing evidence suggests that cargo receptor plays an important role in transporting substrates to autophagosomes for selective degradation [19,33]. Since VANGL2 is not a cargo receptor, we hypothesized that VANGL2 might bridge NLRP3 to the cargo receptor for autophagic degradation. We observed that while VANGL2 and NLRP3 can bind to various cargo receptors (Fig 4H and 4I), only knockout of *OPTN* blocked VANGL2-mediated NLRP3 degradation (Fig 4J), but not other cargo receptors (*p62*, *TOLLIP*, *NDP52*, and *NBR1*) (S4D–S4G Fig), suggesting that VANGL2 may promote NLRP3 degradation through OPTN-mediated selective autophagy. Subsequently, we investigate whether VANGL2 affects the interaction between OPTN and NLRP3. Immunofluorescence staining demonstrated that *Vangl2* knockout resulted in an effective reduction in the co-localization of OPTN and NLRP3 in PEMs (Fig 4K). Co-IP experiments revealed that the knockout or knockdown of *Vangl2* led to decreased binding of NLRP3 to OPTN (Figs 4L and S4H). In contrast, VANGL2 overexpression enhanced the interaction between OPTN and NLRP3 (Fig 4M). Furthermore, VANGL2 increased the interaction between NLRP3 and LC3B (Fig 4N), while knockout of *Vangl2* inhibited this interaction (S4I Fig). In addition, after knocking out of *OPTN*, VANGL2 no longer promoted the binding of NLRP3 to LC3B (S4J Fig). Thus, these data indicate that VANGL2 accelerates the degradation of NLRP3 via OPTN-mediated selective autophagy.

### VANGL2 recruits MARCH8 to promote the K27-linked ubiquitination of NLRP3

**Fig 5:**
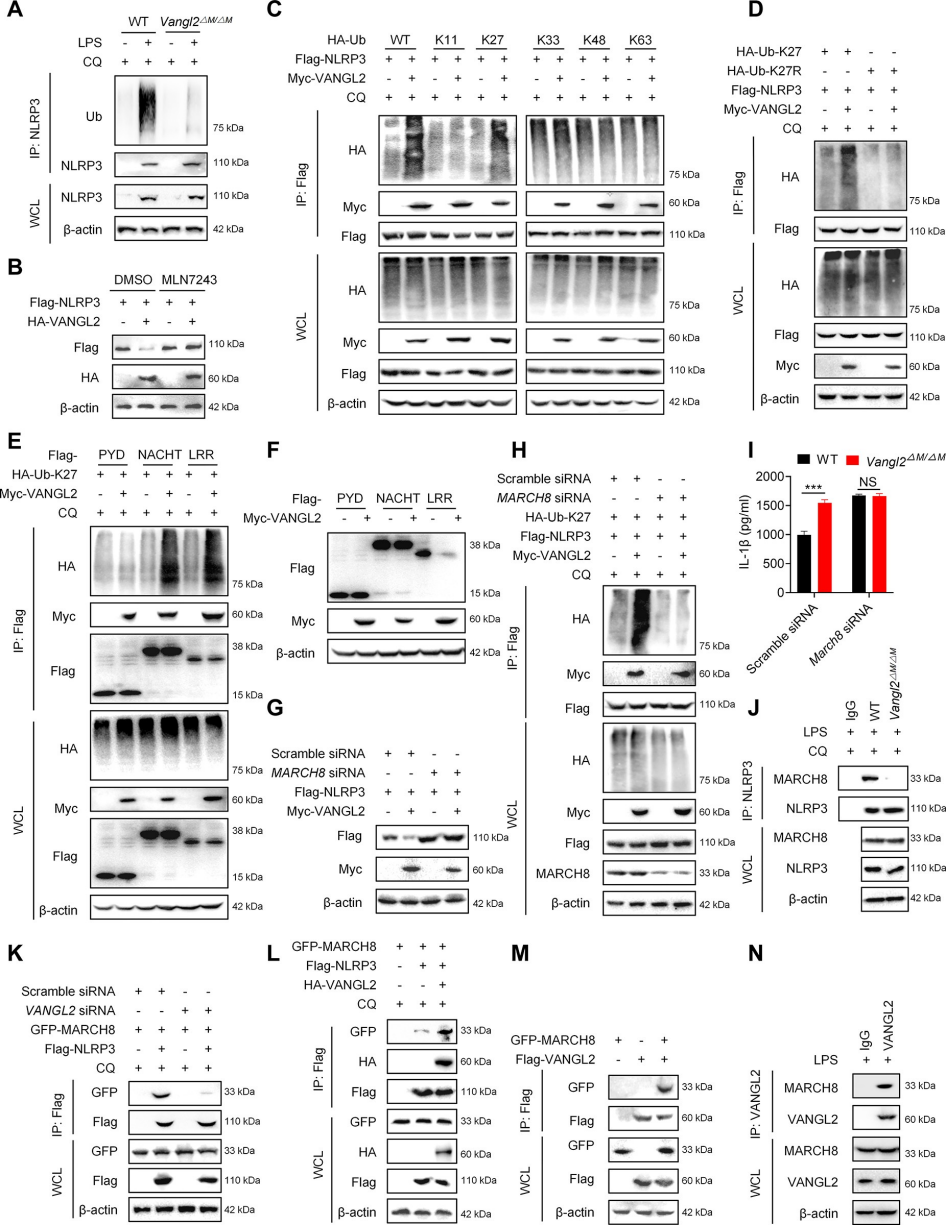
VANGL2 recruits MARCH8 to promote the K27-linked ubiquitination of NLRP3. (A) PEMs were pretreated with LPS (100 ng/ml) and CQ (50 μM) for 6 h, then NLRP3 was pulled down by IP, and the expression of Ub and NLRP3 were detected by immunoblot analysis. (B) Flag-NLRP3 and HA-VANGL2 plasmids were transfected into HEK293T cells for 24 h, followed by treatment with MLN7243 (5 μM) for 6 h. Immunoblot analysis was used to detect the expression of Flag and HA tagged proteins. (C) Different types of ubiquitination plasmids, Flag-NLRP3, and Myc-VANGL2 plasmids were transfected into HEK293T cells for 24 h, followed by treatment with CQ (50 μM) for 6 h. Flag was pulled down by IP, and the expression of HA, Myc, and Flag tagged proteins were detected by immunoblot analysis. (D) HA-Ub-K27, HA-Ub-K27R, Flag-NLRP3, and Myc-VANGL2 plasmids were transfected into HEK293T cells for 24 h, followed by treatment with CQ (50 μM) for 6 h. The Flag was pulled down by IP. The expression of HA, Flag, and Myc tagged proteins were detected by immunoblot analysis. (E) Flag-PYD, Flag-NACHT, Flag-LRR, HA-Ub-K27, and Myc-VANGL2 plasmids were transfected into HEK293T cells for 24 h, followed by treatment with CQ (50 μM) for 6 h. The expression of HA, Myc, and Flag tagged proteins were detected by IP and immunoblot analysis. (F) Flag-PYD (NLRP3), Flag-NACHT (NLRP3), Flag-LRR (NLRP3), and Myc-VANGL2 plasmids were transfected into HEK293T cells for 24 h, and immunoblot analysis was used to detect the expression of Myc and Flag tagged proteins. (G) HEK293T cells were silenced with *MARCH8* siRNA for 24 h, followed by transfection with Flag-NLRP3 and Myc-VANGL2 plasmids for 24 h. The expression of Flag and Myc tagged proteins were detected by immunoblot analysis. (H) HEK293T cells were silenced with *MARCH8* siRNA for 24 h, followed by transfection with HA-Ub-K27, Flag-NLRP3, and Myc-VANGL2 plasmids for 24 h. Flag was then pulled down through IP, and the expression of HA, Flag, and Myc tagged proteins were detected by immunoblot analysis. (I) WT and *Vangl2*^ΔM/ΔM^ PEMs were silenced by *March8* siRNA for 24 h, followed by the treatment of LPS and ATP, and the expression of IL-1β was detected by ELISA. (J) WT and *Vangl2*^ΔM/ΔM^ PEMs were pretreated with LPS (100 ng/ml) and CQ (50 μM) for 6 h, and then NLRP3 was pulled down by IP, and the expression of MARCH8 and NLRP3 were detected by immunoblot analysis. (K) HEK293T cells were silenced by *VANGL2* siRNA for 24 h and then transferred with GFP-MARCH8 and Flag-NLRP3 plasmids for 24 h. Flag was pulled down by IP. Immunoblot analysis was used to detect the expression of GFP and Flag tagged proteins. (L) GFP-MARCH8, Flag-NLRP3, and HA-VANGL2 plasmids were transfected into HEK293T cells for 24 h, and Flag was pulled down by IP. Immunoblot analysis was used to detect the expression of GFP, HA, and Flag tagged proteins. (M) GFP-MARCH8 and Flag-VANGL2 plasmids were transfected into HEK293T cells for 24 h, and Flag was pulled down by IP. The expression of GFP and Flag tagged proteins were detected by immunoblot analysis. (N) PEMs were pretreated with LPS (100 ng/ml) and CQ (50 μM) for 6 h, and then VANGL2 was pulled down by IP, and the expression of MARCH8 and VANGL2 was detected by immunoblot analysis. Data are expressed as means ± SD. ***P < 0.001, NS means not significant. The data underlying this figure can be found in S1 Data and S1 Raw Images.

The ubiquitin chain connected to the substrate serves as the main signal responsible for identifying cargo receptors [34,35]. Subsequently, we investigated whether VANGL2 regulates the ubiquitination of NLRP3 for the recognition by OPTN. Both silencing and knockout of *Vangl2* effectively reduced the poly-ubiquitination of NLRP3 in PEMs (Figs 5A and S5A). Importantly, the ubiquitination inhibitor MLN7243 effectively reversed VANGL2-mediated NLRP3 degradation (Fig 5B). These results suggest that VANGL2 promotes NLRP3 degradation through a ubiquitin-dependent pathway. Next, we co-expressed NLRP3 in the presence or absence of VANGL2, together with various ubiquitin linkages, to explore which type of ubiquitination modification in NLRP3 was regulated by VANGL2. The Co-IP results showed that VANGL2 specifically enhanced K27-linked poly-ubiquitination of NLRP3 (Fig 5C). In contrast, silencing *VANGL2* effectively inhibits the K27-linked ubiquitination of NLRP3 (S5B Fig). Additionally, VANGL2 could not increase the ubiquitination level of NLRP3 when overexpressing mutant HA-Ub-K27R (Fig 5D), indicating that VANGL2 specifically promotes the K27-linked poly-ubiquitination of NLRP3. Subsequently, we proceeded to explore which domain of NLRP3 is regulated by VANGL2 for K27-linked poly-ubiquitination and degradation. The results showed that VANGL2 promoted the K27-linked poly-ubiquitination of the NACHT and LRR domains in NLRP3, but not the PYD domain (Fig 5E). Moreover, VANGL2 only mediated the degradation of LRR domain of NLRP3 (Fig 5F). Therefore, these data show that the LRR domain of NLRP3 is critical for VANGL2-mediated K27-linked poly-ubiquitination and degradation.

Next, we investigated which E3 ubiquitin ligase is recruited by VANGL2 to promote NLRP3 ubiquitination. We analyzed potential E3 ubiquitin ligases for the K27-linked ubiquitination of NLRP3 using UbiBrowser (http://ubibrowser.bio-it.cn/ubibrowser_v3/home/index) [36] and found 4 highest scoring E3 ubiquitin ligases involved, MARCH1, MARCH3, MARCH8, and MARCH11. We then silenced the expression of these E3 ligases by siRNA and found that knockdown of *MARCH8* rescued VANGL2-mediated degradation of NLRP3 (Fig 5G), whereas knockdown of *MARCH1*, *MARCH3*, or *MARCH11* did not (S5C–S5E Fig). MARCH7 was also reported to induce autophagy degradation of NLRP3 [37]; however, knockdown of *MARCH7* was unable to impede VANGL2-mediated K27-linked ubiquitination of NLRP3 (S5F Fig). Meanwhile, silencing of *MARCH7* did not prevent the degradation of NLRP3 by VANGL2 (S5G Fig). Additionally, silencing *March8* promoted NLRP3 stability (S5H Fig) and the release of IL-1β (S5I Fig) in PEMs. Furthermore, VANGL2 no longer increased the poly-ubiquitination of NLRP3 after *MARCH8* silencing (Fig 5H), and *MARCH8* knockdown reversed the increase in IL-1β mediated by *Vangl2* deficiency (Fig 5I). Therefore, these results suggest that VANGL2 mediates the K27-linked poly-ubiquitination of NLRP3 by recruiting E3 ubiquitin ligase MARCH8.

To explore how VANGL2 regulates MARCH8-mediated K27-linked poly-ubiquitination and degradation of NLRP3, we evaluated the binding efficiency and found that *Vangl2* deficiency led to a reduced interaction between NLRP3 and MARCH8 in PEMs (Fig 5J). Similarly, silencing of *VANGL2* also decreased the association between NLRP3 and MARCH8 in HEK293T cells (Fig 5K), while VANGL2 overexpression enhanced this interaction (Fig 5L). Interestingly, we found that MARCH8 directly interacts with VANGL2 in exogenous experiments (Fig 5M). Likewise, the endogenous experiments (PEMs) also yielded consistent results (Fig 5N). Thus, these data suggest that VANGL2 accelerates NLRP3 ubiquitination and autophagic degradation by recruiting MARCH8.

### Lysine 823 in NLRP3 is essential for K27-linked ubiquitination and autophagic degradation

**Fig 6:**
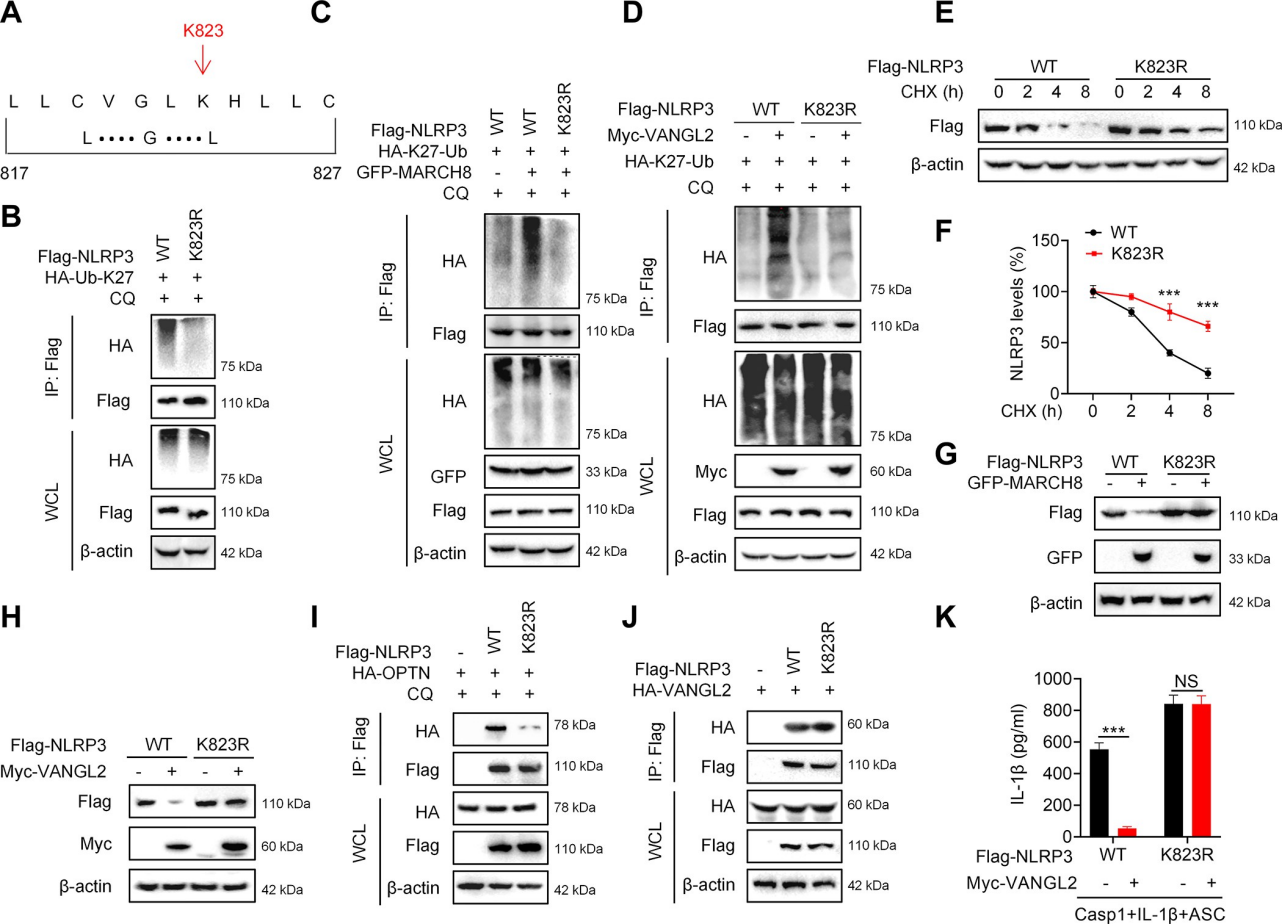
Lys823 in NLRP3 is essential for its K27-linked ubiquitination and autophagic degradation. (A) The motif of the interaction between MARCH8 and NLRP3. (B) Flag-NLRP3 (WT and K823R) and HA-Ub-K27 plasmids were transfected into HEK293T cells for 24 h, followed by CQ (50 μM) treatment for 6 h. Flag was pulled down by IP, and the expressions of HA and Flag tagged proteins were detected by immunoblot analysis. (C) Flag-NLRP3 (WT and K823R), HA-Ub-K27, and GFP-MARCH8 plasmids were transfected into HEK293T cells for 24 h, followed by CQ (50 μM) treatment for 6 h. Flag was pulled down by IP, and the expressions of HA and Flag tagged proteins were detected by immunoblot analysis. (D) Flag-NLRP3 (WT and K823R), HA-Ub-K27, and Mcy-VANGL2 plasmids were transfected into HEK293T cells for 24 h, and then CQ (50 μM) was added for 6 h. Flag was pulled down by IP, and the expression of HA and Flag tagged proteins were detected by immunoblot analysis. (E) Flag-NLRP3 (WT and K823R) plasmids were transfected into HEK293T cells for 24 h, followed by CHX (50 μg/ml) treatment for indicated times. Immunoblot analysis was used to detect the expression of Flag tagged protein. (F) NLRP3 degradation rate for (E). (G) Flag-NLRP3 (WT and K823R) and GFP-MARCH8 plasmids were transfected into HEK293T cells for 24 h. Immunoblot analysis was used to detect the expression of Flag and GFP tagged proteins. (H) Flag-NLRP3 (WT and K823R) and Myc-VANGL2 plasmids were transfected into HEK293T cells for 24 h. The expression of Flag and Myc tagged proteins were detected by immunoblot analysis. (I) Flag-NLRP3 (WT and K823R) and HA-OPTN plasmids were transfected into HEK293T cells for 24 h, followed by CQ (50 μM) treatment for 6 h. Immunoblot analysis was used to detect the expression of Flag and HA tagged proteins. (J) Flag-NLRP3 (WT and K823R) and HA-VANGL2 plasmids were transfected into HEK293T cells for 24 h, then Flag was pulled down by IP, and finally the expression of Flag and HA tagged proteins were detected by immunoblot analysis. (K) Flag-NLRP3 (WT and K823R), Myc-VANGL2, Casp1, IL-1β, and ASC plasmids were transfected into HEK293T cells for 24 h, and the expression of IL-1β was detected by ELISA. Data are expressed as means ± SD. ***P < 0.001, NS means not significant. The data underlying this figure can be found in S1 Data and S1 Raw Images. CHX, cycloheximide; CQ, chloroquine; WT, wild-type.

Lysine residue forms isopeptide-linked ubiquitin chains and is critical for ubiquitin-mediated posttranslational modification [10,36,38]. We then focused on the lysine site of MARCH8-mediated K27-linked ubiquitination in NLRP3. Bioinformatics analysis predicted that MARCH8 had a binding motif in amino acid sequences 817–827 of NLRP3, which includes only one lysine site K823 for ubiquitination (Fig 6A). Indeed, K27-linked ubiquitination of NLRP3 significantly reduced when mutating K823 to K823R (Fig 6B). Meanwhile, the K27-linked ubiquitination of NLRP3 enhanced by MARCH8 was eliminated after K823 mutation in NLRP3 (Fig 6C). Further experiments showed that VANGL2 failed to promote K27-linked ubiquitination of NLRP3 K823R mutant (Fig 6D), indicating that VANGL2-mediated MARCH8 catalyzed K27-linked ubiquitination in NLRP3 is dependent on the K823 site.

Next, we explored whether K823 is the critical site for VANGL2-mediated autophagic degradation of NLRP3. CHX experiment showed that the NLRP3 degradation rate was significantly slowed down after K823 mutation (Fig 6E and 6F). Moreover, neither MARCH8 (Fig 6G) nor VANGL2 (Fig 6H) could promote the degradation of NLRP3 K823R mutant. In addition, the binding of OPTN to NLRP3 was attenuated after K823 mutation (Fig 6I), and K823 mutation also weakened the interaction between NLRP3 and LC3B (S6A Fig). Although K823 mutation did not affect the interaction between VANGL2 and NLRP3 (Fig 6J), VANGL2 could not restrict the activation of inflammasome signaling that NLRP3 K823R mutant induced (Fig 6K). Taken together, these results suggest that VANGL2 promotes OPTN-mediated selective autophagic degradation of NLRP3 is dependent on the K27-linked ubiquitination of NLRP3 at Lys 823.

To explore the 4 involved protein including VANGL2, NLRP3, MARCH8, and OPTN interact with each other and form a complex, we performed the Coomassie blue staining and *in vitro* IP experiments (S6B Fig). The results revealed that NLRP3 directly binds to VANGL2, MARCH8, and OPTN, respectively (S6C–S6E Fig). Meanwhile, VANGL2 also combined with MARCH8 and OPTN (S6F and S6G Fig). However, MARCH8 and OPTN cannot interact with each other (S6H Fig). Furthermore, we also validated the above results through a three-step Co-IP experiment. Our data showed that when NLRP3, VANGL2, MARCH8, and OPTN are present, these 4 proteins can interact with each other and form a protein complex (S6I Fig). Therefore, these results suggest that VANGL2 promotes the ubiquitination of NLRP3 by recruiting MARCH8, which is subsequently recognized and degraded by OPTN, and that these 4 proteins can form a complex.

### VANGL2 suppresses DSS-induced colitis by inhibiting NLRP3 inflammasome activation

**Fig 7:**
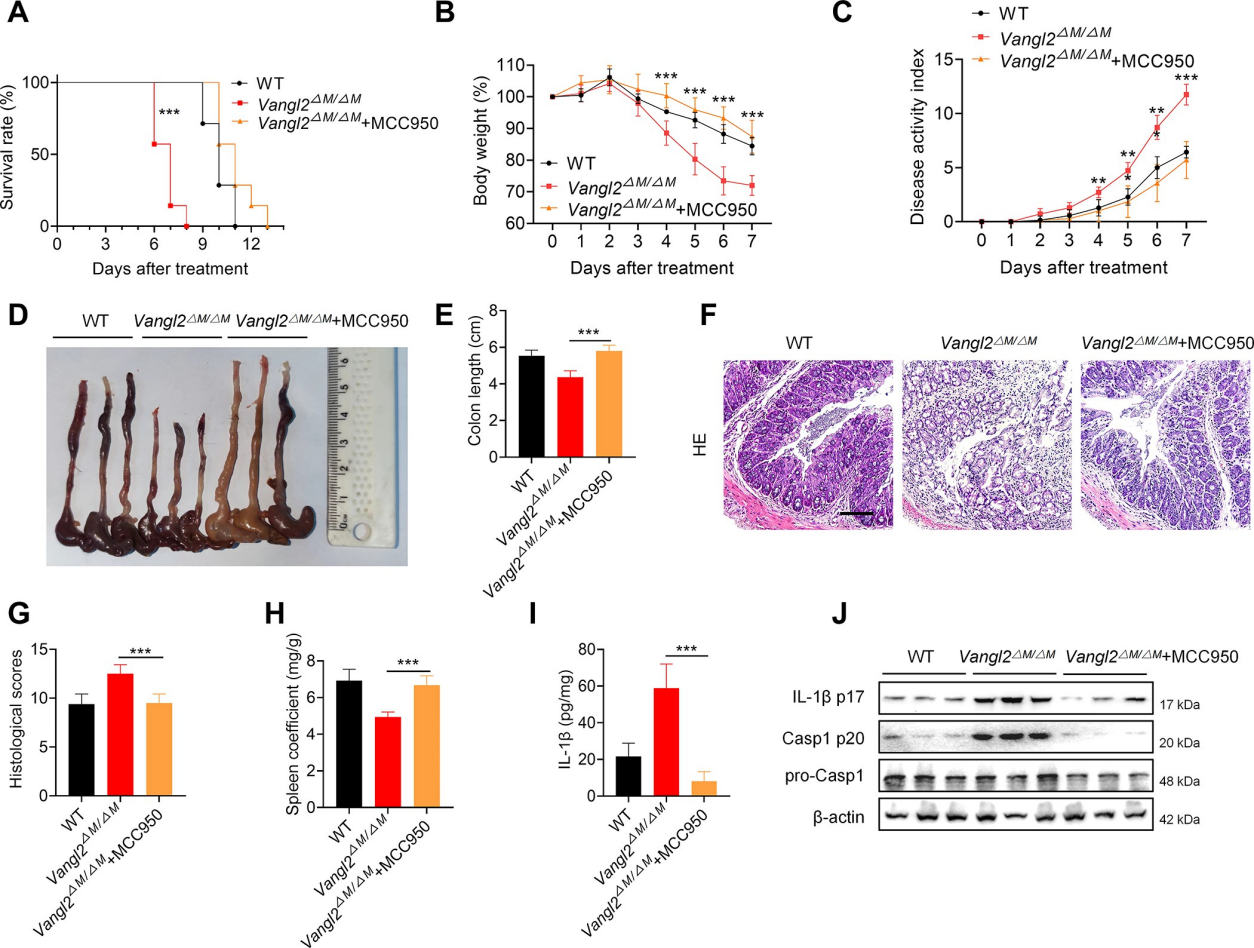
VANGL2 suppresses DSS-induced colitis in mice by inhibiting NLRP3 inflammasome. (A–E) Survival rate (A), body weight change (B), disease activity index (C), intestinal gross tissue (D), and colon length (E) were measured in the WT, *Vangl2*^ΔM/ΔM^, and MCC950 treated *Vangl2*^ΔM/ΔM^ mice after DSS treatment (*n* = 7). (F, G) HE staining was used to detect the histological characteristics of WT, *Vangl2*^ΔM/ΔM^, and MCC950 treated *Vangl2*^ΔM/ΔM^ mice after DSS treatment (*n* = 8). Scale bar = 100 μm. (H) Spleen coefficient of WT, *Vangl2*^ΔM/ΔM^, and MCC950 treated *Vangl2*^ΔM/ΔM^ mice after DSS treatment. (I) ELISA was used to detect the expression of IL-1β in the intestinal supernatant of WT, *Vangl2*^ΔM/ΔM^, and MCC950 treated *Vangl2*^ΔM/ΔM^ mice after DSS treatment (*n* = 10). (J) Immunoblot analysis was used to detect the expression of IL-1β p17, Casp1 p20, and pro-Casp1 in WT, *Vangl2*^ΔM/ΔM^, and MCC950 treated *Vangl2*^ΔM/ΔM^ mice after DSS treatment. Data are expressed as means ± SD. **P < 0.01, ***P < 0.001. The data underlying this figure can be found in S1 Data. DSS, dextran sulfate sodium; VANGL2, Van-Gogh-like 2; WT, wild-type.

To further confirm whether VANGL2 alleviates DSS-induced colitis by affecting NLRP3 inflammasome activation, we inhibited NLRP3 inflammasome by MCC950 during DSS treatment *in vivo* and observed that the survival rate of *Vangl2*^ΔM/ΔM^ mice was lower than that of WT mice, while MCC950 treatment effectively improved the survival rate of *Vangl2*^ΔM/ΔM^ mice and was comparable to WT mice (Fig 7A). Meanwhile, MCC950 treatment effectively alleviated the weight loss, restored the disease activity index, and avoided the shortening of the intestinal tract in *Vangl2*^ΔM/ΔM^ mice (Fig 7B–7E). HE staining results suggested that MCC950 treatment down-regulated the intestinal inflammatory infiltration and histological scores in *Vangl2*^ΔM/ΔM^ mice (Fig 7F and 7G), and Alcian blue staining showed that MCC950 significantly increased the number of goblet cells in *Vangl2*^ΔM/ΔM^ mice (S7A Fig). Moreover, the spleen index of *Vangl2*^ΔM/ΔM^ mice was recovered after MCC950 treatment (Fig 7H). Consistently, MCC950 effectively decreased the release of IL-1β (Fig 7I) and reversed the up-regulation of IL-1β and Casp1 in *Vangl2*^ΔM/ΔM^ mice (Fig 7J). Taken together, these data suggest that VANGL2 alleviates the progression of DSS-induced colitis in mice by blocking the NLRP3 inflammasome activation.

We overexpressed WT MARCH8 or MARCH8 enzymatic mutation (MARCH8-W114A for human, and MARCH8-W110A for mouse) in *MARCH8*-KO cells to observe whether VANGL2 affects the activation of inflammasome activation relies on the E3 enzymatic activity of MARCH8. The data showed that VANGL2 did not inhibit the activation of NLRP3 inflammasome in the presence of enzymatic activity mutant of MARCH8 (S7B Fig). These findings indicate that the regulation of NLRP3 inflammasome activation by VANGL2 is dependent on the enzymatic activity of MARCH8. Furthermore, *March8*-WT and *March8*-W110A chimeric mice were constructed to investigate the effect of VANGL2 on the progression of DSS-induced colitis. The results showed that VANGL2 did not affect NLRP3 inflammasome activation and colitis progression in *March8*-W110A mice compared to *March8*-WT mice (S7C–S7I Fig). Thus, VANGL2 inhibites NLRP3 inflammasome activation and IBD progression via the MARCH8 enzyme activity-dependent pathway.

### VANGL2 suppresses NLRP3 inflammasome activation in human PBMCs

**Fig 8:**
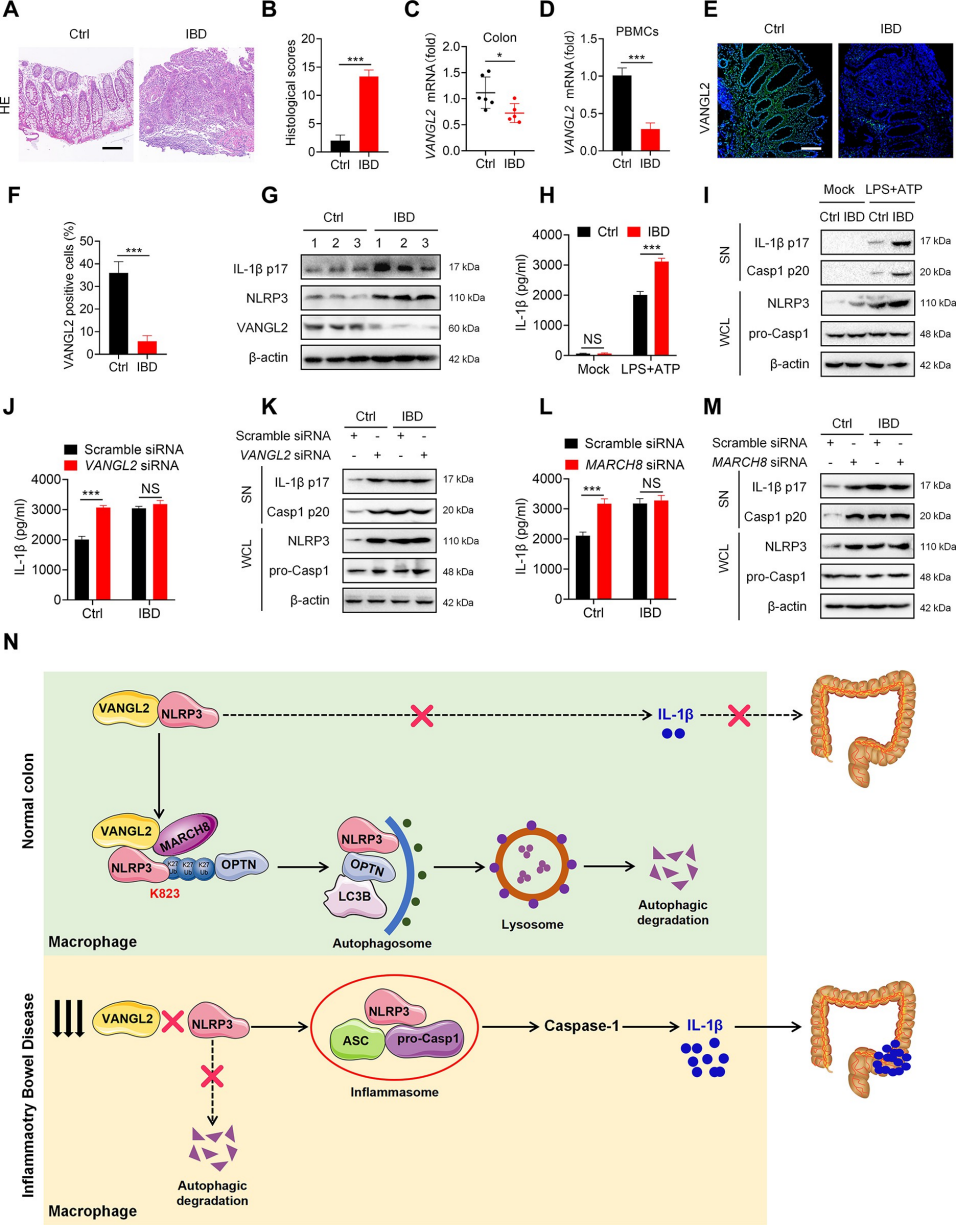
Down-regulation of VANGL2 in IBD patients triggers overt activation of NLRP3 inflammasome. (A) HE staining was used to compare the colonic morphology between health control and IBD patients. Scale bar = 100 μm. (B) Statistical analysis of histological scores for (A). (C) The mRNA expression of *VANGL2* in colon of healthy control and IBD patients was detected by qRT-PCR. (D) qRT-PCR was used to detect the *VANGL2* mRNA expression of PBMCs in healthy controls and IBD patients. (E) The expression of VANGL2 in colon of healthy control and IBD patients was detected by immunofluorescence staining. Scale bar = 100 μm. (F) Statistical analysis of VANGL2 positive cells for (E). (G) Immunoblot analysis was used to detect the expression of NLRP3 inflammasome-associated proteins in the colon of healthy control and IBD patients. (H, I) The PBMCs of health control and IBD patients were treated with LPS (100 ng/ml) for 6 h, followed by ATP (5 mM) for 30 min. Culture supernatants were then collected to measure IL-1β release by ELISA (H). Cell lysates and supernatants were collected for immunoblot analysis (I). (J, K) The PBMCs of health control and IBD patients were transfected with scramble siRNA or *VANGL2* siRNA for 24 h, followed by LPS (100 ng/ml) for 6 h, and then treated with ATP (5 mM) for 30 min. Culture supernatants were then collected to measure IL-1β release by ELISA (J). Cell lysates and supernatants were collected for immunoblot analysis (K). (L, M) The PBMCs of health control and IBD patients were transfected with scramble siRNA or *MARCH8* siRNA for 24 h, followed by LPS (100 ng/ml) for 6 h, and then treated with ATP (5 mM) for 30 min. Culture supernatants were then collected to measure IL-1β release by ELISA (l). Cell lysates and supernatants were collected for immunoblot analysis (M). (N) Schematic diagram of the action of VANGL2 in IBD. Data are expressed as the means ± SD. *P < 0.05, ***P < 0.001, NS means not significant. The data underlying this figure can be found in S1 Data and S1 Raw Images. IBD, inflammatory bowel disease; VANGL2, Van-Gogh-like 2.

Next, to determine the function and relevance of VANGL2 and NLRP3 inflammasome in IBD patients, we detected the expression of VANGL2 and NLRP3 in the colon of IBD patients (Fig 8A and 8B) and observed that *VANGL2* mRNA expression was reduced in colon tissue and PBMCs from IBD patients (Fig 8C and 8D). Immunofluorescence staining and immunoblot analysis also revealed a down-regulation of VANGL2 protein in the colons of patients with IBD (Fig 8E and 8F). Furthermore, the inflammasome proteins IL-1β and NLRP3 showed a negative correlation with VANGL2 expression in the colon (Fig 8G). Taken together, these results suggest that VANGL2 is down-regulated in IBD patients and reverse-correlated with the expression of NLRP3 inflammasome.

Subsequently, we explored the impact of VANGL2 on the activation of NLRP3 inflammasome in human immune cells. We first silenced *VANGL2* in THP-1 cells (human macrophage cell line) and found that silencing of *VANGL2* effectively promoted the secretion of IL-1β under the condition of LPS and ATP stimulation (S8A Fig). Likewise, *MARCH8* knockdown also yielded consistent conclusions (S8B Fig). Next, we purified PBMCs from IBD patients or healthy individuals and observed that the release of IL-1β in PBMCs of IBD patients was significantly higher than that of normal controls under the stimulation of LPS and ATP (Fig 8H). Meanwhile, immunoblot analysis showed significantly enhanced expression of NLRP3, IL-1β, and Casp1 in PBMCs from IBD patients (Fig 8I). Notably, silence of *VANGL2* or *MARCH8* enhanced the expression of IL-1β, Casp1, and NLRP3 in healthy PBMCs, but could not further promote the expression of IL-1β, Casp1, and NLRP3 in IBD PBMCs under the condition of NLRP3 inflammasome activation (Fig 8J–8M). Thus, these data reveal a correlation between the down-regulation of VANGL2 and overt NLRP3 inflammasome activation in IBD patients.

## Discussion

In this study, we show that VANGL2 is down-regulated in human and mouse colitis and negatively correlated with the progression of IBD. Myeloid knockout of *Vangl2* enhances the activation of NLRP3 inflammasome and exacerbates the progression of DSS-induced colitis in mice. Mechanistically, VANGL2 targets NLRP3 and recruits E3 ubiquitin ligase MARCH8 to increase the K27-linked poly-ubiquitination of NLRP3, thereby accelerating OPTN-mediated selective autophagic degradation and ultimately inhibiting NLRP3 inflammasome activation. Moreover, NLRP3 inhibitor MCC950 effectively reversed the exacerbation of colitis triggered by *Vangl2* deficiency. Notably, knockdown of *VANGL2* or *MARCH8* effectively enhanced NLRP3 inflammasome activation in PBMCs from healthy individuals, but not from IBD patients, which strongly suggest a role for VANGL2 in anti-colitis progression *via* the NLRP3 inflammasome pathway.

VANGL2 is a highly conserved four-channel transmembrane protein, which belongs to the core complex responsible for mediating the Wnt/PCP pathway [25,27]. Knockout of VANGL2 in glomerular podocytes has been reported to promote renal inflammatory response, increase MMP9 expression, and damage renal tissue [39]. Additionally, VANGL2 plays an important role in the paracrine Wnt/β-catenin signaling by controlling the behavior of cell filaments [40]. VANGL2 also inhibits osteogenesis and plays a pathogenic role in osteoporosis [25]. Conversely, the loss of VANGL2 in hematopoietic stem cells leads to bone marrow transplantation failure [41]. VANGL2 benefits the self-renewal of satellite cells and promotes muscle regeneration [42]. In addition to its developmental function, our recent studies indicate that VANGL2 can also modulate anti-viral immunity and sepsis progression [18,30], broadening its role in regulating immune responses. Although several studies have linked VANGL2 to IBD [27,29,43], there has been no comprehensive investigation into its role and mechanism in this disease. Here, our study has identified that VANGL2 plays a protective role in the process of IBD and further expanded upon our findings by demonstrating that VANGL2 regulates the autophagic degradation of NLRP3 through distinct mechanisms.

NLRP3 is regulated by various PTMs, including acetylation, phosphorylation, SUMOylation, palmitoylation, and ubiquitination [16,23,44,45]. These modifications result in distinct NLRP3 functions. Palmitoylation induces CMA degradation, thus limiting NLRP3 inflammasome activation [12]. In addition, SUMOylation and acetylation of NLRP3 inhibit NLRP3 inflammasome [11,44]. In contrast, the phosphorylation of the NLRP3 LRR domain actively contributes to inflammasome assembly [9]. Furthermore, ubiquitination and deubiquitination are critical for activating NLRP3 inflammasome [13,16]. E3 ubiquitin ligase MARCH7 induces the K48-linked ubiquitination leading to autophagic degradation of NLRP3, thereby inhibiting NLRP3 inflammasome activation [15]. Conversely, the deubiquitinating enzyme BRCC3 cleaves the K48- and K63-linked ubiquitin chains on NLRP3, stabilizing NLRP3 protein and resulting in NLRP3 inflammasome activation [10,45]. Here, we discovered that VANGL2 recruits E3 ubiquitin enzyme MARCH8 to increase NLRP3 ubiquitination and autophagic degradation, leading to negative regulation of the NLRP3 inflammasome. Although both MARCH7 and MARCH8 can promote the autophagic degradation of NLRP3, they mediate different types of ubiquitin chains, which lead to varied outcomes. Reports suggest that the K63-linked ubiquitin chain is a crucial signal for NLRP3 autophagic degradation [10], whereas the K48-linked ubiquitin chain mediates both proteasome and autophagic degradation of NLRP3 [10,13,15]. In addition, HUWE1 promotes the K27-linked ubiquitination of NLRP3 and activates the NLRP3 inflammasome directly [46]. Conversely, β-Trcp promotes proteasome degradation of NLRP3 by K27-linked ubiquitination, ultimately inhibiting NLRP3 inflammasome activation [47]. Our research indicates that VANGL2 mediates K27-linked ubiquitination of NLRP3, which in turn is involved in the autophagic degradation of NLRP3. Therefore, the K27-linked ubiquitination of NLRP3 mediated by different molecules is capable of producing diverse functions and results. Additionally, the fate of NLRP3 is determined by the ubiquitination that takes place at different lysine sites. For instance, ubiquitination at K21, K22, and K24 of NLRP3 is reported to promote NLRP3 inflammasome activation [10], while ubiquitination at K380, K496, and K689 sites of NLRP3 inhibits NLRP3 inflammasome activation [10]. Here, we discovered that ubiquitination at K823 promotes the autophagic degradation of NLRP3 and inhibits the activation of the NLRP3 inflammasome. Therefore, precise regulation of ubiquitination at various lysine sites in NLRP3 is crucial for affecting the activation of NLRP3 inflammasome.

Although NLRP3 could be localized on the cell membrane [48,49], several studies also suggested that NLRP3 is dynamically shuttled and transported in the cytoplasm [50–53]. In our experimental system, we performed an immunoblot analysis of NLRP3 in the cytoplasm and cell membrane. However, the presence of an excessive amount of cytoplasmic NLRP3 masked a small quantity of NLRP3 expressed in the cell membrane, thereby preventing the detection of NLRP3 expression in the short exposure (SE) of immunoblot analysis experiments. Nevertheless, the expression of cell membrane NLRP3 was successfully discerned in the long exposure (LE) of immunoblot analysis experiments. Meanwhile, only cytoplasmic NLRP3 could be precipitated by VANGL2. Therefore, NLRP3 is present in both the cytoplasm and the cell membrane, and VANGL2 primarily interacts with NLRP3 in the cytoplasm.

Autophagy is a conservative degradation pathway that decomposes and circulates dysfunctional cellular components [19]. Accumulating evidence reveals the link between autophagy and NLRP3 inflammasome signaling [15,20,54]. Various factors, including IRGM, Galectin-9, USP5, CCDC50, and USP22, have reportedly participated in the autophagic degradation of NLRP3 [15,20,54–56]. Selective autophagy is distinguished from non-selective autophagy in that its mechanism must effectively recognize and isolate cargo proteins during autophagy [19]. However, the precise mechanism of selective autophagy in NLRP3 inflammasome activation is not yet well understood. The previous study has identified p62, CCDC50, and NBR1 as the autophagic cargo receptors of NLRP3 [15,20,54–56]. Here, we have discovered that OPTN serves as another cargo receptor and participates in the selective autophagic degradation of NLRP3. Our findings are in line with the existing literature, showcasing an inverse relationship between OPTN and NLRP3 expression [57]. Although it has been reported that OPTN inhibits NLRP3 inflammasome activation through the enhancement of mitochondrial autophagy, this phenomenon is achieved under high glucose stimulation [57]. Since our experimental system is based on LPS stimulation and DSS treatment, it is evident that the mechanism by which OPTN regulates the NLRP3 inflammasome differs between various diseases and cellular environments. Furthermore, confocal microscopy revealed that OPTN co-localized with NLRP3, which was further enhanced in the presence of VANGL2, indicating that VANGL2 forms a complex with OPTN and NLRP3, ultimately accelerating the autophagic degradation of NLRP3. OPTN is reported to have a tendency to bind the K63-linked ubiquitin chain and promote the autophagic degradation of KDM4D [58]. In addition, our recent research has shown that OPTN has the capability to identify the K48-linked ubiquitin chain of TBK1 and trigger the process of TBK1 autophagic degradation [18]. Notably, here we further demonstrated that OPTN can recognize the K27-linked ubiquitin chain of NLRP3. Consequently, our study expands our comprehension of OPTN’s negative regulation of NLRP3 mechanism and reinforces our recognition of cargo receptors required for the selective autophagic degradation of NLRP3.

In recent years, numerous studies have demonstrated that autophagy plays a critical regulatory role in the IBD process [8,20,22]. Autophagy is essential for maintaining the intestinal barrier and mucosal homeostasis [20,22]. Moreover, a low level of autophagy is frequently observed in patients with IBD [20,22]. Several risk genes associated with IBD, including ATG16L1, NOD2, IRGM, and LRRK2, are highly associated with immune cell and intestinal epithelial cell autophagy deficiency [8]. Here, we further discovered that the IBD-associated gene VANGL2 significantly regulates autophagy and inflammation in macrophages, providing a new perspective for further exploring the relationship between autophagy and IBD. Notably, NLRP3 inflammasome plays a crucial role in promoting the progression of IBD [6]. Previous studies have reported that excessive secretion of IL-1β in IBD patients and mouse colitis models [3]. Meanwhile, NLRP3 inflammasome activation and excessive release of IL-1β are significant factors contributing to the exacerbation of IBD symptoms [3]. Our study showed that the NLRP3 inflammasome is activated in both IBD patients and DSS-induced colitis in mice. Furthermore, our study provides a novel explanation for the hyperactivation of NLRP3 inflammasome during IBD: VANGL2 down-regulation hinders the K27-linked ubiquitination of NLRP3, decreases OPTN-mediated autophagic degradation of NLRP3, and results in overactivation of the NLRP3 inflammasome, thus promoting IBD progression.

Due to the important role of intestinal epithelial cells in IBD, we also investigated the function of VANGL2 in intestinal epithelial cells and found that knocking out VANGL2 in intestinal epithelial cells can exacerbate IBD progression. This once again confirms the protective role of VANGL2 in IBD. Meanwhile, *Vangl2*^flox/flox^ *lyz*-cre mice were worse than the *Vangl2*^flox/flox^ *Villin*-cre mice in DSS-induced colitis. However, the mechanism of VANGL2 in myeloid cells and intestinal epithelial cells may be distinct, and further research is needed to explore it.

Taken together, we identified VANGL2 as a negative regulator of NLRP3 inflammasome and involved in regulating the pathogenesis of DSS-induced colitis. Based on our findings, we propose a working model to illustrate how VANGL2 plays a protective role in colitis (Fig 8N). In normal intestinal macrophages, VANGL2 binds to NLRP3 and recruits the E3 ubiquitin ligase MARCH8 to promote the K27-linked ubiquitination of NLRP3 at K823. By recognizing the K27-linked polyubiquitin chain on NLRP3, OPTN delivers NLRP3 to autolysosomes for degradation, thereby maintaining the low expression state of NLRP3. Conversely, the degradation of NLRP3 is relieved due to the decreased expression of VANGL2 in IBD, resulting in excessive activation of the NLRP3 inflammasome and promoting the release of IL-1β, ultimately accelerating the progression of IBD. Our research presents evidence of VANGL2’s crucial role in restraining NLRP3 inflammasome activation, thus advancing our comprehension of the required mechanism for therapeutic intervention in IBD.

## Materials and methods

### Ethics statement

All animal experiments in this study were performed in accordance with the institutional ethical guidelines for animal experiments and were approved by the Southern Medical University Animal Care and Use Committee (Animal ethical license number: SMUL20201010).

### Antibodies

Anti-IL-1β, anti-Casp1, and anti-NLRP3 were obtained from ImmunoWay Biotechnology (Plano, Texas, United States of America). Anti-pro-Casp1 was obtained from ABclonal (Woburn, Massachusetts, USA). Anti-VANGL2 and anti-Actin were obtained from Santa Cruz Biotechnology. Anti-ATG5, anti-BECN1, anti-OPTN, anti-TOLLIP, anti-p62, anti-NDP52, anti-Na(+)/K(+) ATPase 1 (ATP1A1), and anti-LC3B were obtained from Proteintech. Anti-Ub (P4D1), anti-ASC, and anti-GFP were obtained from Cell Signaling Technology. Anti-Flag, anti-HA, anti-GFP, and anti-Myc were purchased from Sigma.

### Mice

*Vangl2*^flox/flox^ (Stock No: 025174) and *Lyz2*-Cre (Stock No: 4781) mice on a C57BL/6 background were obtained from the Jackson Laboratory (Las Vegas, Nevada, USA). *Vangl2*^flox/flox^ mice were crossed with *Lyz2*-Cre mice whose age and sex matched to obtain *Vangl2*^flox/flox^ *Lyz2*-Cre (*Vangl2*^ΔM/ΔM^) mice with Vangl2-specific deficiency in myeloid cells. Co-housed littermate controls with normal VANGL2 expression were used as controls. *Nlrp3*^-/-^ mice were provided by Dr. Zi Li (Guangzhou Medical University). All animal experiments were performed in specific pathogen-free levels using 6- to 8-week-old mice and approved.

As for chimeric mice, the construction of *March8*-WT, *March8*-W110A, or *Vangl2* overexpression lentiviral plasmids and virus packaging were produced by OBiO (Shanghai, China), then infected *March8* KO bone marrow (BM) cells according to the following 4 groups: *March8*-WT, *March8*-WT+*Vangl2*, *March8*-W110A, and *March8*-W110A+*Vangl2*. The lentiviral plasmids vetor used is pcSLenti-EF1-EGFP-P2A-Puro-CMV. The recipient mice aged 6 to 8 weeks (male, WT C57BL/6) were irradiated with 4.5 Gy, then irradiated again with 4 Gy 4 h later, and randomly divided into 4 groups to receive lentivirus-treated BM cells (5 × 10^6^), respectively. Chimeric mice were kept in SPF animal houses and used for the construction of DSS-mediated colitis after 6 to 8 weeks.

### Human samples

All patients with IBD were recruited from the Department of Gastroenterology at Nanfang Hospital, affiliated with Southern Medical University (Guangzhou, China). Positive subjects and patients with other severe illnesses or chronic diseases were excluded from the study. The diagnosis of CD or UC was based on clinical, radiologic, and endoscopic examinations and histologic findings. All patients provided informed consent, and the study was approved by the Ethics Committee of Nanfang Hospital, Southern Medical University (NFEC-2023-437).

### Induction of DSS-mediated colitis

Eight-week-old male mice were induced to develop acute colitis by 2.5% (w/v) of DSS dissolved in drinking water for 7 days or until the end of the experiment. The DSS solution was made freshly every 2 days. To inhibit the NLRP3 inflammasome, mice were injected intraperitoneally (*i*.*p*.) with MCC950 (20 mg/kg) 1 day before DSS treatment at 2-day intervals. The control group was injected intraperitoneally with saline. During DSS treatment, mice were monitored for body weight daily, while diarrhea and rectal bleeding were determined by disease activity index (DAI) score. In brief, stool scores were assigned as follows [20]: 0, well-formed pellets; 1, semi-solid; 2, semi-solid and not adhere to the anus; 3, liquid and adhere to the anus; 4, diarrhea. Bleeding scores were assigned as follows: 0, no blood in stool; 1, light faint; 2, clear visible; 3, gross rectal bleeding. Clinical scores were assigned as follows: 0, no weight loss; 1, weight loss of 1%–5%; 2, 6%–10%; 3, 11%–20%; 4, more than 20%. At the end of the experiment, mice were sacrificed to measure colon length, and colon homogenates were collected to detect cytokines.

### Cells and treatments

Mouse PEMs were acquired from ascites of indicated mice, which were injected intraperitoneally (*i*.*p*.) with 4% Brewer thioglycollate medium (BD Bioscience) for 3 consecutive days before sacrifice. Cold PBS (Bio-Channel, Nanjing, China) was injected into the peritoneum of sacrificed mice. Then, fluid containing peritoneal macrophages was aspirated from the peritoneum after the shake and collected after centrifugation at 800 rpm for 5 min. For mouse BMDMs, mice were sacrificed by cervical dislocation, and bone marrow cells were flushed from mouse tibias and femurs. BM cells were cultured in L-929 conditional medium (70% DMEM, 10% fetal bovine serum, 20% L-929 medium, 100 U/ml penicillin, and 100 μg/ml streptomycin) for 5 days. The PBMCs isolated from IBD patients and age- and sex-matched healthy volunteers (Nanfang Hospital of Southern Medical University) were collected in BD Vacutainer CPT tubes. PBMCs were cultured in RPMI 1640 medium supplemented with 10% fetal bovine serum (HyClone) and 1% L-glutamine. THP-1 cells were cultured in RPMI 1640 medium supplemented with 10% fetal bovine serum (HyClone) and 1% L-glutamine. THP-1 cells were differentiated into macrophages by treatment with 100 ng/ml phorbol-12-myristate-13-acetate (PMA; Sigma) for 12 h and cultured with RPMI 1640. HEK293T cells were cultured in DMEM medium (Corning) supplemented with 10% fetal bovine serum (Vazyme, Nanjing, China), 100 U/ml penicillin, and 100 μg/ml streptomycin (Sigma-Aldrich). HEK293T cells of *p62* KO, *TOLLIP* KO, *NDP52* KO, and *NBR1* KO were obtained from Dr. Jun Cui (Sun Yat-sen University). In some experiments, cells were treated with or without LPS (100 ng/ml, 6 h), ATP (5 mM, 30 min), Nigericin (10 μM, 30 min), MSU (200 μg/ml, 6 h), gDNA (1 μg/ml, 12 h), Poly(dA: dT) (1 μg/ml, 4 h), and MCC950 (1 μM) for the indicated time to test cytokines. Cycloheximide (CHX) (Selleck) (50 μg/ml) was used to block protein synthesis. EBSS (Thermo Fisher Scientific) was used for autophagy inducement. MG132 (Ambeed, Cat#A181909) (10 μM) was used to inhibit proteasome-mediated protein degradation; 3-MA (Selleck) (10 mM) or CQ (50 μM) was used to inhibit autolysosome-or lysosome-mediated protein degradation.

### Transfection of plasmids and siRNA

Plasmids for VANGL2 and NLRP3 and their mutants were cloned into the pcDNA3.1 vector with an indicated tag for transient expression. HEK293T and THP-1 transfection was performed using Lipofectamine 2000 (Invitrogen) according to protocols recommended by the manufacturer. Plasmids for cargo protein were kindly provided by Dr. Jun Cui (Sun Yat-sen University). Chemically synthesized 21-nucleotide siRNA duplexes were obtained from TranSheepBio and transfected using Lipofectamine RNAiMAX (Invitrogen) according to the manufacturer’s instructions.

### Enzyme-linked immunosorbent assay (ELISA)

IL-1β in cell supernatants and mice colon homogenates was measured using an ELISA kit (ABclonal, Woburn, Massachusetts, USA) following the manufacturer’s instructions. Absorbance was detected at 450 nm by the Multiscan FC (Thermo Fisher Scientific).

### Co-immunoprecipitation (Co-IP) and immunoblot analysis

For endogenous Co-IP, whole-cell lysates were prepared after transfection or stimulation, followed by incubation overnight with the appropriate antibodies plus Protein A/G beads (Pierce) as per manufacturer’s instructions. For exogenous Co-IP, whole-cell lysates were only incubated with anti-Flag or anti-Myc agarose beads (Sigma). Immunoprecipitates were then washed 5 times with low-salt lysis buffer (50 mM HEPES, 150 mM NaCl, 1 mM EDTA, 10% glycerol, 1.5 mM MgCl, and 1% Triton X-100), and immunoprecipitates were eluted with 2×SDS Loading Buffer and resolved by SDS-PAGE. Proteins were transferred to a polyvinylidene difluoride membrane (Millipore). The membranes were blocked with 5% (w/v) reagent-grade nonfat milk and further incubated with the antibodies with universal antibody diluent (NCM Biotech, Suzhou, China) as listed in the Antibodies section. EMD Millipore Luminata Western HRP Chemiluminescence Substrate was used for protein detection for all blots.

### Three-step co-immunoprecipitation

HEK293T cells were transfected with Flag-NLRP3, HA-VANGL2, GFP-MARCH8, and Myc-OPTN plasmids for 24 h, and then the cells were lysed. For the first step of immunoprecipitation, the cell lysate was incubated with Flag-beads for 4 h. Then, the Flag-beads were washed 3 times. Next, the Flag-beads were divided into 2, one for western blot, and the other for incubation with Flag peptide for 1.5 h. Subsequently, centrifugation was performed and the supernatant was retained. For the second step of immunoprecipitation, HA-beads were added to the supernatant of the first step for incubation for 4 h. After incubation, the HA-beads were divided into 2, one for western blot, and the other for incubation with HA peptide for 1.5 h. After incubation, centrifugation was performed and the supernatant was retained. For the third step of immunoprecipitation, GFP-beads were added to the supernatant of the second step for incubation for 4 h. Then, the GFP-beads were washed 3 times in lysis buffer. Finally, the immuno-complexes were resolved by SDS-PAGE and analyzed by western blot.

### *In vitro* binding assays

*In vitro* binding assays were performed as described previously [54,59]. HEK293T cells were transfected with Flag-NLRP3. After transfection for 24 h, and then lysed in an NP-40 lysis buffer. Anti-Flag beads were applied to immunoprecipitate Flag-NLRP3. The beads were washed 5 times with a lysis buffer containing 500 mM NaCl and then eluted with the Flag peptide (Sigma-Aldrich). Recombinant Flag tagged proteins (VANGL2, MARCH8, and OPTN) were purified in the same way as NLRP3. Then, Flag-NLRP3 was incubated with recombinant Flag tagged proteins at 37°C for 1 h, followed by Coomassie brilliant blue staining, IP assay, and immunoblot analysis.

### Cellular fractionation

Briefly, cells were collected by trypsin-EDTA and washed 2 times with PBS, after centrifugation at 500 g for 5 min at 4°C, the cytosolic and membrane fractions of the harvested samples were further isolated using Subcellular Protein Fractionation Kit (78840, Thermo Scientific) according to the manufacturer’s protocol. After that, the isolated parts were processed for immunoblot analysis or Co-IP assay as stated, cytosol-located β-actin and membrane protein Na^+^K^+^ATPase were used to demonstrate the efficiency of the cellular fractionation.

### Confocal microscopy

Cells were cultured on Glass Bottom culture dishes (Nest Scientific) and processed as needed. The cells were washed 3 times using PBS, fixed with 4% paraformaldehyde for 15 min, and then permeabilized in 0.2% Triton X-100 for 20 min. After washing with PBS for 3 times, cells were blocked in 5% BSA for 60 min at room temperature and then incubated with primary antibodies at 4°C overnight. The cells were washed and followed by fluorescent dye-conjugated secondary antibodies (Alexa Fluor 488 goat anti-Rabbit IgG H&L (#ab150077, Abcam) and 594 Goat Anti-mouse IgG H&L (#ab150116, Abcam)) for 1 h at room temperature. The nuclei were counterstained with DAPI (Sigma-Aldrich) for 5 min before being subjected to confocal microscopy observation (100 NA oil-immersion objective, FV3000).

### Histological analysis and immunofluorescence

Intestines were removed from mice and fixed in 4% paraformaldehyde (Sigma) for 24 h. Fixed specimens were washed by running water for 30 min and processed for paraffin embedding and section cutting. For histological analysis, the sections were stained with hematoxylin-eosin (HE) and Alcian blue. For immunofluorescence, the sections of intestine were incubated with secondary antibodies conjugated to fluorophores for 1 h at room temperature. The samples were mounted with 4′, 6-diamidino-2-phenylindole (Invitrogen). The number of positive cells was counted by ImagePro 6.0 software (Media Cybernetics).

### RNA isolation and quantitative real-time PCR (qRT-PCR)

The total RNA was extracted using a Trizol reagent according to the manufacturer’s protocols (Invitrogen). Moreover, the complementary cDNA was generated using Starscript II first-stand cDNA synthesis kit (GenStar, Beijing, China, Cat#A214). Realtime PCR was performed on QuantStudio 6 flex (Thermo Fisher Scientific) using 2×RealStar green power mixture (Genstar, Beijing, China, Cat#A308) with primers. The sequences of primers are listed in S1 Table.

### Statistical analysis

Data were independently repeated at least 3 times or experiments were performed in triplicate. Data represent one of 3 biological replicates, with at least 3 technical replicates each. The results are shown as the means ± standard deviation (SD). Statistical analyses were performed using the SPSS version 20.0 software, and graphs were generated with GraphPad Prism version 8.0 (GraphPad Software, La Jolla, California, USA). Differences between 2 groups were analyzed using Student’s *t* tests. For comparison among more than 2 groups, a one-way analysis of variance (ANOVA) followed by Tukey’s or Dunnett’s multiple comparison test was used. For comparisons among multiple groups and different time points, a two-way analysis of variance followed by Sidak’s or Dunnett’s multiple comparisons test was used. Survival data from in vivo experiments were analyzed by a log-rank test. *P* < 0.05 was considered as a statistically significant difference.

## Supporting information

S1 Fig VANGL2 expression is down-regulated during IBD progression. (A) Bioinformatics analysis of *VANGL2* mRNA levels in human CD and UC. (B) Immunoblot analysis was used to detect the protein expression of VANGL2 in colon of DSS-treated mice at indicated times. (C) PCR assay for gene knockout identification in WT and *Vangl2*^ΔM/ΔM^ mice. (D) The knockout efficiency of *Vangl2* in myeloid cells was determined by immunoblot analysis. (E) The mRNA levels of miR-335 in IBD patients were detected using qPCR. (F) The mRNA levels of miR-335 in DSS-induced colitis were detected using qPCR. (G) BMDMs were pretreated with LPS for 4 h, followed by miR-335 mimic transfection, and finally VANGL2 expression was measured by immunoblot analysis. (H) Detection of *VANGL2* mRNA expression in active and inactive IBD using qPCR. Data are shown as means ± SD. ***P < 0.001.
(PDF)

S2 Fig Silencing *Vangl2* promotes the activation of NLRP3 inflammasome in macrophages. (A) PEMs were silenced with scramble siRNA or *Vangl2* siRNA for 24 h, and the expression of VANGL2 was detected by immunoblot analysis. (B) PEMs were transfected with scramble siRNA or *Vangl2* siRNA for 24 h, followed by the treatment of LPS (100 ng/ml) for 6 h, and then inflammasome-related agonists were added for indicated times. Finally, the expression of IL-1β was detected by ELISA. (C) BMDMs were transfected with scramble siRNA or *Vangl2* siRNA for 24 h, followed by LPS (100 ng/ml) treatment for 6 h, and then added the inflammasome-related activators for indicated times. Finally, ELISA was used to detect the expression of the IL-1β. (D) PEMs were transfected by scramble siRNA or *Vangl2* siRNA for 24 h, followed by LPS (100 ng/ml) treatment for 6 h, and then added NLRP3 inflammasome agonists. Finally, the expression of inflammasome-related proteins were detected by immunoblot analysis. (E) Immunoblot analysis was used to detect the expression of p-p65, p65, p-IκBα, and IκBα in the colon of WT and *Vangl2*^ΔM/ΔM^ mice with DSS-induced colitis. (F, G) ELISA was used to detect the expression of IL-6 (F) and TNF-α (G) in the colon of WT and *Vangl2*^ΔM/ΔM^ mice with DSS-induced colitis. Data are shown as means ± SD. *P < 0.05, **P < 0.01, NS means not significant.
(PDF)

S3 Fig The interacting domains between VANGL2 and NLRP3. (A) HA-NLRP3 and Flag-VANGL2 plasmids were transfected into HEK293T cells for 24 h, and Flag was pulled down by IP assay, and the expression of HA and Flag tagged proteins were detected by immunoblot analysis. (B) PEMs were pretreated with LPS (100 ng/ml) for 6 h, followed by Co-IP to pull down VANGL2, and the expressions of NLRP3 and VANGL2 were detected by immunoblot analysis. (C) GFP-VANGL2 and Flag-NLRP3 plasmids were transfected into HEK293T cells for 24 h, and then the co-localization of VANGL2 and NLRP3 was detected by immunofluorescence staining. Scale bar = 5 μm. (D) HA-NLRP3 and Flag-VANGL2 plasmids were transfected into HEK293T cells for 24 h, and then membrane proteins and cytoplasmic proteins were isolated by cellular fractionation, followed by Co-IP to pull down Flag. The expression of HA-NLRP3 and Flag-VANGL2 were detected by immunoblot analysis. SE, short exposure; LE, long exposure. (E) Truncations of Myc-VANGL2 and Flag-NLRP3 plasmids were transfected into HEK293T cells for 24 h, and the binding of VANGL2 to NLRP3 was detected by Co-IP and immunoblot analysis.
(PDF)

S4 Fig VANGL2 recruits NLRP3 to autophagy receptor OPTN for selective autophagic degradation. (A) Flag-ASC and HA-VANGL2 plasmids were transfected into HEK293T cells for 24 h, and the expression of Flag and HA-tagged proteins were detected by immunoblot analysis. (B) Flag-Casp1 and HA-VANGL2 plasmids were transfected into HEK293T cells for 24 h, and the expression of Flag and HA-tagged proteins were detected by immunoblot analysis. (C) LPS-primed PEMs (WT and *Vangl2*^ΔM/ΔM^) were cultured in EBSS for 0–3 h. Immunoblot analysis was used to detect the expression of NLRP3 and VANGL2. (D) Flag-NLRP3 and HA-VANGL2 plasmids were transfected into WT and *p62* KO HEK293T cells, and the expression of Flag-NLRP3, HA-VANGL2, and p62 were detected by immunoblot analysis. (E) Flag-NLRP3 and HA-VANGL2 plasmids were transfected into WT and *TOLLIP* KO HEK293T cells, and the expression of Flag-NLRP3, HA-VANGL2, and TOLLIP were detected by immunoblot analysis. (F) Flag-NLRP3 and HA-VANGL2 plasmids were transfected into WT and *NDP52* KO HEK293T cells, and the expression of Flag-NLRP3, HA-VANGL2, and NDP52 were detected by immunoblot analysis. (G) Flag-NLRP3 and HA-VANGL2 plasmids were transfected into WT and *NBR1* KO HEK293T cells, and the expression of Flag-NLRP3, HA-VANGL2, and NBR1 were detected by immunoblot analysis. (H) HEK293T cells were silenced with scramble siRNA and *VANGL2* siRNA for 24 h, and then transferred with HA-NLRP3 and Flag-OPTN plasmids for 24 h, followed by treatment with CQ (50 μM) for 6 h. The expression of HA and Flag tagged proteins were detected by Co-IP and immunoblot analysis. (I) WT and *Vangl2*^ΔM/ΔM^ PEMs were pretreated with LPS (100 ng/ml) and CQ (50 μM) for 6 h, and then NLRP3 was pulled down by IP. The expression of LC3B and NLRP3 were detected by immunoblot analysis. (J) Flag-NLRP3, GFP-LC3B, and HA-VANGL2 plasmids were transfected into WT and *OPTN* KO HEK293T cells for 24 h, and then treated with CQ (50 μM) for 6 h. The expression of GFP, Flag, and HA tagged proteins were detected by Co-IP and immunoblot analysis.
(PDF)

S5 Fig VANGL2 recruits MARCH8 to promote the K27-linked ubiquitination of NLRP3. (A) PEMs were silenced with *Vangl2* siRNA for 24 h, followed by LPS (100 ng/ml) and CQ (50 μM) treatment for 6 h, and NLRP3 was pulled down by IP. Immunoblot analysis was used to detect the expression of Ub and NLRP3. (B) HKE293T cells were silenced with *VANGL2* siRNA for 24 h, then transfected with HA-Ub-K27 and Flag-NLRP3 plasmids for 24 h, followed by CQ (50 μM) treatment for 6 h. Flag was pulled down by IP, and the expression of HA and Flag tagged proteins were detected by immunoblot analysis. (C–E) HKE293T cells were silenced with *MARCH1* siRNA (C), *MARCH3* siRNA (D), or *MARCH11* siRNA (E) for 24 h, and then transfected with Flag-NLRP3 and Myc-VANGL2 plasmids for 24 h. Subsequently, the expression of Flag and Myc-tagged proteins were detected by immunoblot analysis. (F) HEK293T cells were silenced with *MARCH7* siRNA for 24 h, followed by transfection with HA-Ub-K27, Flag-NLRP3, and Myc-VANGL2 plasmids for 24 h. Flag was then pulled down through IP, and the expression of HA, Flag, and Myc tagged proteins were detected by immunoblot analysis. (G) HEK293T cells were silenced with *MARCH7* siRNA for 24 h, followed by transfection with Flag-NLRP3 and Myc-VANGL2 plasmids for 24 h. The expression of Flag, Myc, and MARCH7 proteins were detected by immunoblot analysis. (H) PEMs were silenced with *March8* siRNA for 24 h, followed by LPS (100 ng/ml) treatment for 6 h, and finally the expression of NLRP3 was detected by immunoblot analysis. (I) PEMs were silenced with *March8* siRNA for 24 h, followed by LPS (100 ng/ml) treatment for 6 h and ATP treatment for 30 min. The expression of IL-1β was detected by ELISA. Data are expressed as means ± SD. ***P < 0.001.
(PDF)

S6 Fig VANGL2, NLRP3, MARCH8, and OPTN interact and form a complex. (A) Flag-NLRP3 (WT and K823R) and GFP-LC3B plasmids were transfected into HEK293T cells for 24 h and then treated with CQ (50 μM) for 6 h. Flag was pulled down by IP, and the expression of GFP and Flag tagged proteins were detected by immunoblot analysis. (B) Purified VANGL2, NLRP3, MARCH8, and OPTN were detected by Coomassie brilliant blue staining. (C–H) The purified proteins were mixed in a co-IP buffer to perform an in vitro affinity-isolation assay, and the results were analyzed via immunoblot assay. (I) HEK293T cells were transfected with Flag-NLRP3, HA-VANGL2, GFP-MARCH8, and Myc-OPTN. A three-step co-immunoprecipitation assay was performed with the cell lysates. Flag immunoprecipitates, HA immunoprecipitates, and GFP immunoprecipitates were analyzed by immunoblotting.
(PDF)

S7 Fig VANGL2 inhibits NLRP3 inflammasome activation and DSS-induced colitis progression through the MARCH8 E3 enzyme activity-dependent pathway. (A) Alcian blue staining was used to detect the intestinal characteristics of WT mice, *Vangl2*^ΔM/ΔM^ mice, and MCC950 treated *Vangl2*^ΔM/ΔM^ mice after DSS treatment. Scale bar = 100 μm. (B) Inflammasome-related plasmids (NLRP3, ASC, pro-Casp1, and IL-1β), HA-VANGL2, and GFP-MARCH8 (WT, W114A) plasmids were transfected into HEK293T cells (*MARCH8* KO) for 24 h, and the expression of IL-1β was detected by ELISA. (C–I) The construction of March8-WT, March8-W110A, or Vangl2 overexpression lentiviral plasmids and virus packaging were used to infect *March8* KO bone marrow (BM) cells according to the following 4 groups: March8-WT, March8-WT+Vangl2, March8-W110A, March8-W110A+Vangl2. Recipient mice aged 6–8 weeks were irradiated with 4.5 Gy, then irradiated again with 4 Gy 4 h later, and randomly divided into 4 groups to receive lentivirus-treated BM cells, respectively. Chimeric mice were kept in SPF condition and used for the construction of DSS-mediated colitis after 6–8 weeks. The survival rate (C), weight change (D), disease activity index (E), colon length (F and G), HE staining of colon (H), and colon IL-1β (I) were measured. Data are expressed as means ± SD. *P < 0.05, ***P < 0.01, ***P < 0.001, NS means not significant.
(PDF)

S8 Fig VANGL2 and MARCH8 inhibit the activation of NLRP3 inflammasome in THP-1 cells. (A) THP-1 cells were transfected with scramble siRNA or *VANGL2* siRNA for 24 h, followed by LPS (100 ng/ml) for 6 h and ATP (5 mM) for 30 min. Culture supernatants were collected to measure IL-1β release by ELISA. (B) THP-1 cells were transfected with scramble siRNA or *MARCH8* siRNA for 24 h, followed by LPS (100 ng/ml) for 6 h and ATP (5 mM) for 30 min. Culture supernatants were collected to measure IL-1β release by ELISA. ***P < 0.001, NS means not significant.
(PDF)

S1 Table Primers and siRNA sequences. (DOCX)

S1 Data Underlying data for the tables. (XLSX)

S1 Raw Images Raw images for all western blots. (PDF)

## Abbreviations

BM: bone marrow
BMDM: bone marrow-derived macrophage
CD: Crohn’s disease
CHX: cycloheximide
CQ: chloroquine
DAI: disease activity index
DSS: dextran sulfate sodium
EBSS: Earle’s balanced salt solution
ELISA: enzyme-linked immunosorbent assay
HE: hematoxylin-eosin
IBD: inflammatory bowel disease
LE: long exposure
LRR: leucine-rich repeat
PCP: planar cellular polarity
PEM: peritoneal macrophage
PTM: posttranslational modification
SD: standard deviation
SE: short exposure
UC: ulcerative colitis
VANGL2: Van-Gogh-like 2
WT: wild-type
