# Supplementary material for: VANGL2 alleviates inflammatory bowel disease by recruiting the ubiquitin ligase MARCH8 to limit NLRP3 inflammasome activation through OPTN-mediated selective autophagy
Source: PLoS Biol. 2025 Feb 3;23(2):e3002961. doi: 10.1371/journal.pbio.3002961 (PMC11790156; doi:10.1371/journal.pbio.3002961)
Supplement: S1 Fig — (A) Bioinformatics analysis of VANGL2 mRNA levels in human CD and UC. (B) Immunoblot analysis was used to detect the protein expression of VANGL2 in colon of DSS-treated mice at indicated times. (C) PCR assay for gene knockout identification in WT and Vangl2ΔM/ΔM mice. (D) The knockout efficiency of Vangl2 in myeloid cells was determined by immunoblot analysis. (E) The mRNA levels of miR-335 in IBD patients were detected using qPCR. (F) The mRNA levels of miR-335 in DSS-induced colitis were detected using qPCR. (G) BMDMs were pretreated with LPS for 4 h, followed by miR-335 mimic transfection, and finally VANGL2 expression was measured by immunoblot analysis. (H) Detection of VANGL2 mRNA expression in active and inactive IBD using qPCR. Data are shown as means ± SD. ***P < 0.001. (PDF) [file pbio.3002961.s001.pdf]

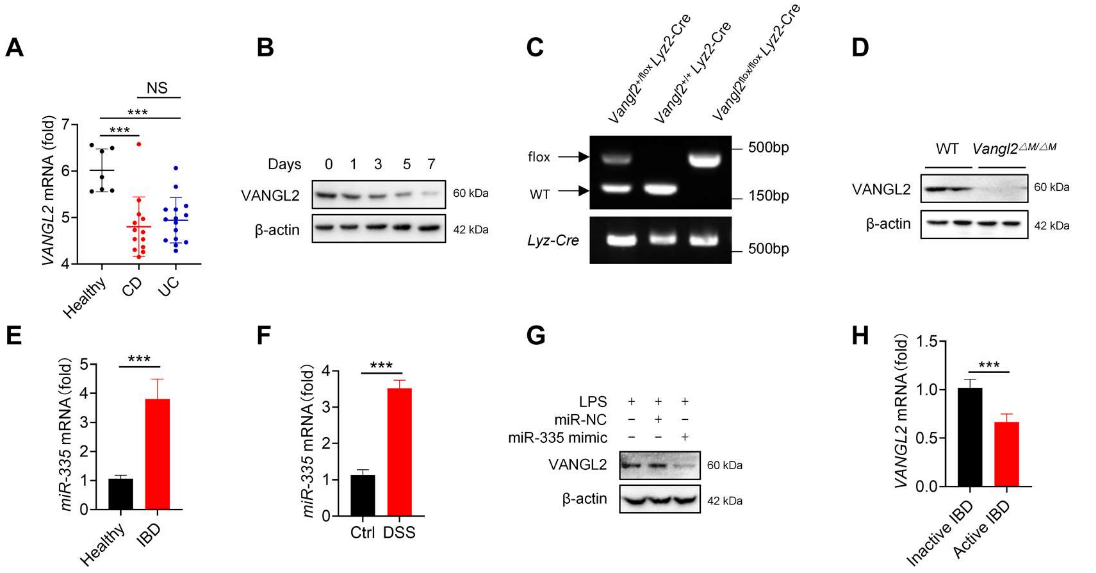

S1 Fig. VANGL2 expression is downregulated during IBD progression.

(A) Bioinformatics analysis of *VANGL2* mRNA levels in human CD and UC. (B) Immunoblot analysis was used to detect the protein expression of VANGL2 in colon of DSS-treated mice at indicated times. (C) PCR assay for gene knockout identification in WT and *Vangl2*$^{\Delta M/\Delta M}$ mice. (D) The knockout efficiency of *Vangl2* in myeloid cells was determined by immunoblot analysis. (E) The mRNA levels of miR-335 in IBD patients were detected using qPCR. (F) The mRNA levels of miR-335 in DSS-induced colitis were detected using qPCR. (G) BMDMs were pretreated with LPS for 4 h, followed by miR-335 mimic transfection, and finally VANGL2 expression was measured by immunoblot analysis. (H) Detection of VANGL2 mRNA expression in active and inactive IBD using qPCR. Data are shown as means ± SD. $^{***}P < 0.001$. The data underlying this Figure can be found in S1 Data and S1 Raw Images.
