# Supplementary material for: VANGL2 alleviates inflammatory bowel disease by recruiting the ubiquitin ligase MARCH8 to limit NLRP3 inflammasome activation through OPTN-mediated selective autophagy
Source: PLoS Biol. 2025 Feb 3;23(2):e3002961. doi: 10.1371/journal.pbio.3002961 (PMC11790156; doi:10.1371/journal.pbio.3002961)
Supplement: S2 Fig — (A) PEMs were silenced with scramble siRNA or Vangl2 siRNA for 24 h, and the expression of VANGL2 was detected by immunoblot analysis. (B) PEMs were transfected with scramble siRNA or Vangl2 siRNA for 24 h, followed by the treatment of LPS (100 ng/ml) for 6 h, and then inflammasome-related agonists were added for indicated times. Finally, the expression of IL-1β was detected by ELISA. (C) BMDMs were transfected with scramble siRNA or Vangl2 siRNA for 24 h, followed by LPS (100 ng/ml) treatment for 6 h, and then added the inflammasome-related activators for indicated times. Finally, ELISA was used to detect the expression of the IL-1β. (D) PEMs were transfected by scramble siRNA or Vangl2 siRNA for 24 h, followed by LPS (100 ng/ml) treatment for 6 h, and then added NLRP3 inflammasome agonists. Finally, the expression of inflammasome-related proteins were detected by immunoblot analysis. (E) Immunoblot analysis was used to detect the expression of p-p65, p65, p-IκBα, and IκBα in the colon of WT and Vangl2ΔM/ΔM mice with DSS-induced colitis. (F, G) ELISA was used to detect the expression of IL-6 (F) and TNF-α (G) in the colon of WT and Vangl2ΔM/ΔM mice with DSS-induced colitis. Data are shown as means ± SD. *P < 0.05, **P < 0.01, NS means not significant. (PDF) [file pbio.3002961.s002.pdf]

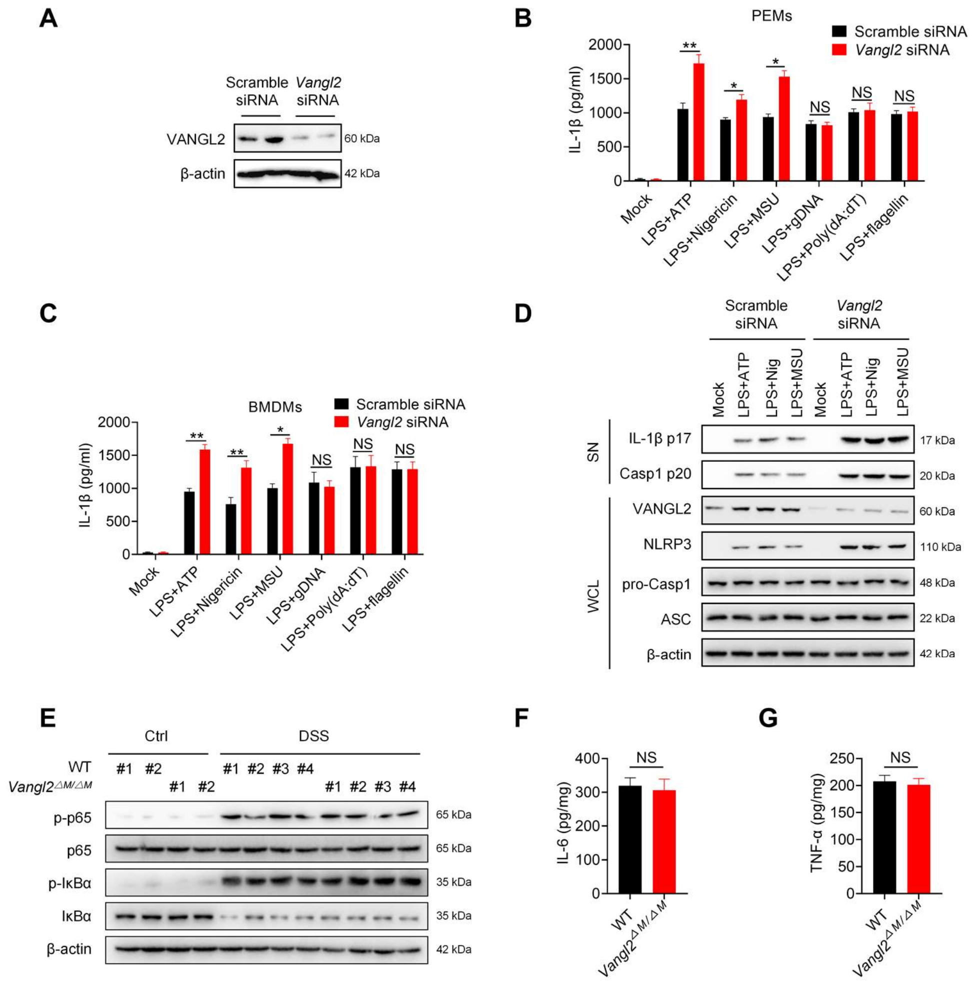

S2 Fig. Silencing *Vangl2* promotes the activation of NLRP3 inflammasome in macrophages.

(A) PEMs were silenced with scramble siRNA or *Vangl2* siRNA for 24 h, and the expression of VANGL2 was detected by immunoblot analysis. (B) PEMs were transfected with scramble siRNA or *Vangl2* siRNA for 24 h, followed by the treatment of LPS (100 ng/mL) for 6 h, and then inflammasome-related agonists were added for indicated times. Finally, the expression of IL-1β was detected by ELISA. (C) BMDMs were transfected with scramble siRNA or *Vangl2* siRNA for 24 h, followed by LPS (100 ng/mL) treatment for 6 h, and then added the inflammasome-related activators for indicated times. Finally, ELISA was used to detect the expression of the IL-1β. (D) PEMs were transfected by scramble siRNA or *Vangl2* siRNA for 24 h, followed by LPS (100 ng/mL) treatment for 6 h, and then added NLRP3 inflammasome agonists. Finally, the expression of inflammasome-related proteins were detected by

immunoblot analysis. (E) Immunoblot analysis was used to detect the expression of p-p65, p65, p-IκBα, and IκBα in the colon of WT and *Vangl2*$^{\Delta M/\Delta M}$ mice with DSS-induced colitis. (F, G) ELISA was used to detect the expression of IL-6 (F) and TNF-α (G) in the colon of WT and *Vangl2*$^{\Delta M/\Delta M}$ mice with DSS-induced colitis. Data are shown as means ± SD. $^{*}P<0.05$, $^{**}P<0.01$, NS means not significant. The data underlying this Figure can be found in S1 Data and S1 Raw Images.
