# Supplementary material for: VANGL2 alleviates inflammatory bowel disease by recruiting the ubiquitin ligase MARCH8 to limit NLRP3 inflammasome activation through OPTN-mediated selective autophagy
Source: PLoS Biol. 2025 Feb 3;23(2):e3002961. doi: 10.1371/journal.pbio.3002961 (PMC11790156; doi:10.1371/journal.pbio.3002961)
Supplement: S3 Fig — (A) HA-NLRP3 and Flag-VANGL2 plasmids were transfected into HEK293T cells for 24 h, and Flag was pulled down by IP assay, and the expression of HA and Flag tagged proteins were detected by immunoblot analysis. (B) PEMs were pretreated with LPS (100 ng/ml) for 6 h, followed by Co-IP to pull down VANGL2, and the expressions of NLRP3 and VANGL2 were detected by immunoblot analysis. (C) GFP-VANGL2 and Flag-NLRP3 plasmids were transfected into HEK293T cells for 24 h, and then the co-localization of VANGL2 and NLRP3 was detected by immunofluorescence staining. Scale bar = 5 μm. (D) HA-NLRP3 and Flag-VANGL2 plasmids were transfected into HEK293T cells for 24 h, and then membrane proteins and cytoplasmic proteins were isolated by cellular fractionation, followed by Co-IP to pull down Flag. The expression of HA-NLRP3 and Flag-VANGL2 were detected by immunoblot analysis. SE, short exposure; LE, long exposure. (E) Truncations of Myc-VANGL2 and Flag-NLRP3 plasmids were transfected into HEK293T cells for 24 h, and the binding of VANGL2 to NLRP3 was detected by Co-IP and immunoblot analysis. (PDF) [file pbio.3002961.s003.pdf]

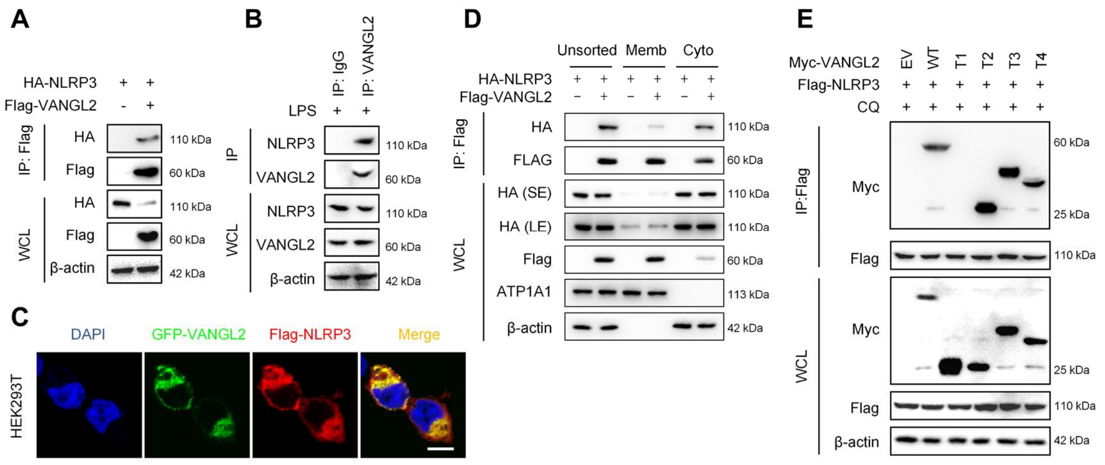

S3 Fig. The interacting domains between VANGL2 and NLRP3.

(A) HA-NLRP3 and Flag-VANGL2 plasmids were transfected into HEK293T cells for 24 h, and Flag was pulled down by IP assay, and the expression of HA and Flag tagged proteins were detected by immunoblot analysis. (B) PEMs were pretreated with LPS (100 ng/mL) for 6 h, followed by Co-IP to pull down VANGL2, and the expressions of NLRP3 and VANGL2 were detected by immunoblot analysis. (C) GFP-VANGL2 and Flag-NLRP3 plasmids were transfected into HEK293T cells for 24 h, and then the co-localization of VANGL2 and NLRP3 was detected by immunofluorescence staining. Scale bar=5 μm. (D) HA-NLRP3 and Flag-VANGL2 plasmids were transfected into HEK293T cells for 24 h, and then membrane proteins and cytoplasmic proteins were isolated by cellular fractionation, followed by Co-IP to pull down Flag. The expression of HA-NLRP3 and Flag-VANGL2 were detected by immunoblot analysis. SE, short exposure; LE, long exposure. (E) Truncations of Myc-VANGL2 and Flag-NLRP3 plasmids were transfected into HEK293T cells for 24 h, and the binding of VANGL2 to NLRP3 was detected by Co-IP and immunoblot analysis. The data underlying this Figure can be found in S1 Raw Images.
