# Supplementary material for: VANGL2 alleviates inflammatory bowel disease by recruiting the ubiquitin ligase MARCH8 to limit NLRP3 inflammasome activation through OPTN-mediated selective autophagy
Source: PLoS Biol. 2025 Feb 3;23(2):e3002961. doi: 10.1371/journal.pbio.3002961 (PMC11790156; doi:10.1371/journal.pbio.3002961)
Supplement: S4 Fig — (A) Flag-ASC and HA-VANGL2 plasmids were transfected into HEK293T cells for 24 h, and the expression of Flag and HA-tagged proteins were detected by immunoblot analysis. (B) Flag-Casp1 and HA-VANGL2 plasmids were transfected into HEK293T cells for 24 h, and the expression of Flag and HA-tagged proteins were detected by immunoblot analysis. (C) LPS-primed PEMs (WT and Vangl2ΔM/ΔM) were cultured in EBSS for 0–3 h. Immunoblot analysis was used to detect the expression of NLRP3 and VANGL2. (D) Flag-NLRP3 and HA-VANGL2 plasmids were transfected into WT and p62 KO HEK293T cells, and the expression of Flag-NLRP3, HA-VANGL2, and p62 were detected by immunoblot analysis. (E) Flag-NLRP3 and HA-VANGL2 plasmids were transfected into WT and TOLLIP KO HEK293T cells, and the expression of Flag-NLRP3, HA-VANGL2, and TOLLIP were detected by immunoblot analysis. (F) Flag-NLRP3 and HA-VANGL2 plasmids were transfected into WT and NDP52 KO HEK293T cells, and the expression of Flag-NLRP3, HA-VANGL2, and NDP52 were detected by immunoblot analysis. (G) Flag-NLRP3 and HA-VANGL2 plasmids were transfected into WT and NBR1 KO HEK293T cells, and the expression of Flag-NLRP3, HA-VANGL2, and NBR1 were detected by immunoblot analysis. (H) HEK293T cells were silenced with scramble siRNA and VANGL2 siRNA for 24 h, and then transferred with HA-NLRP3 and Flag-OPTN plasmids for 24 h, followed by treatment with CQ (50 μM) for 6 h. The expression of HA and Flag tagged proteins were detected by Co-IP and immunoblot analysis. (I) WT and Vangl2ΔM/ΔM PEMs were pretreated with LPS (100 ng/ml) and CQ (50 μM) for 6 h, and then NLRP3 was pulled down by IP. The expression of LC3B and NLRP3 were detected by immunoblot analysis. (J) Flag-NLRP3, GFP-LC3B, and HA-VANGL2 plasmids were transfected into WT and OPTN KO HEK293T cells for 24 h, and then treated with CQ (50 μM) for 6 h. The expression of GFP, Flag, and HA tagged proteins were detected by Co-IP and immunoblot analysis. (PDF) [file pbio.3002961.s004.pdf]

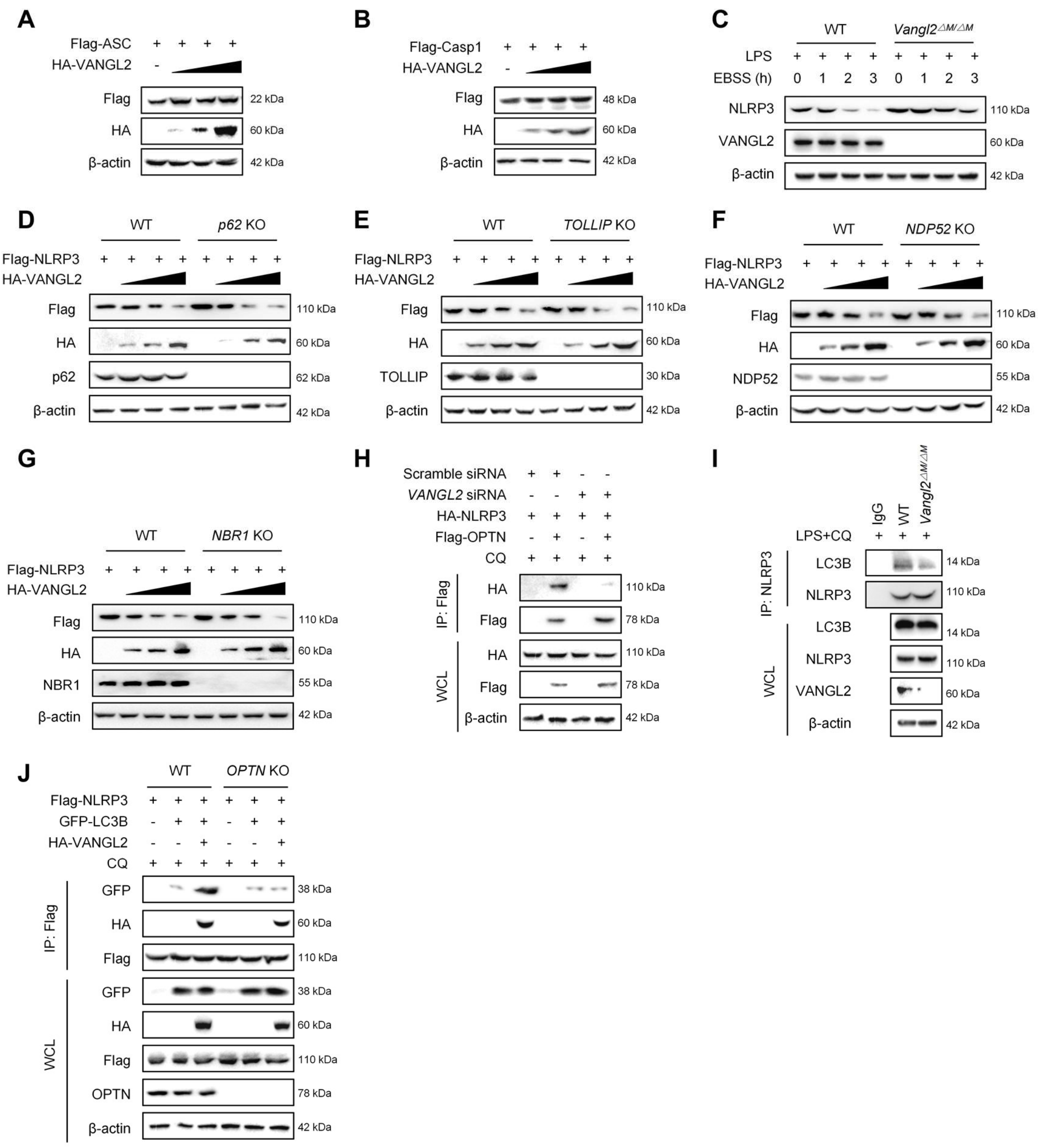

S4 Fig. VANGL2 recruits NLRP3 to autophagy receptor OPTN for selective autophagic degradation.

(A) Flag-ASC and HA-VANGL2 plasmids were transfected into HEK293T cells for 24 h, and the expression of Flag and HA-tagged proteins were detected by immunoblot analysis. (B) Flag-Casp1 and HA-VANGL2 plasmids were transfected into HEK293T cells for 24 h, and the expression of Flag and HA-tagged proteins were detected by immunoblot analysis. (C) LPS-primed PEMs (WT and *Vangl2*$^{\Delta M/\Delta M}$) were cultured in EBSS for 0-3 h. Immunoblot analysis was used to detect the expression of NLRP3 and VANGL2. (D) Flag-NLRP3 and HA-VANGL2 plasmids were transfected into WT

and *p62* KO HEK293T cells, and the expression of Flag-NLRP3, HA-VANGL2, and p62 were detected by immunoblot analysis. (E) Flag-NLRP3 and HA-VANGL2 plasmids were transfected into WT and *TOLLIP* KO HEK293T cells, and the expression of Flag-NLRP3, HA-VANGL2, and TOLLIP were detected by immunoblot analysis. (F) Flag-NLRP3 and HA-VANGL2 plasmids were transfected into WT and *NDP52* KO HEK293T cells, and the expression of Flag-NLRP3, HA-VANGL2, and NDP52 were detected by immunoblot analysis. (G) Flag-NLRP3 and HA-VANGL2 plasmids were transfected into WT and *NBR1* KO HEK293T cells, and the expression of Flag-NLRP3, HA-VANGL2, and NBR1 were detected by immunoblot analysis. (H) HEK293T cells were silenced with scramble siRNA and *VANGL2* siRNA for 24 h, and then transferred with HA-NLRP3 and Flag-OPTN plasmids for 24 h, followed by treatment with CQ (50 μM) for 6 h. The expression of HA and Flag tagged proteins were detected by Co-IP and immunoblot analysis. (I) WT and *Vangl2*$^{\Delta M/\Delta M}$ PEMs were pretreated with LPS (100 ng/mL) and CQ (50 μM) for 6 h, and then NLRP3 was pulled down by IP. The expression of LC3B and NLRP3 were detected by immunoblot analysis. (J) Flag-NLRP3, GFP-LC3B, and HA-VANGL2 plasmids were transfected into WT and *OPTN* KO HEK293T cells for 24 h, and then treated with CQ (50 μM) for 6 h. The expression of GFP, Flag, and HA tagged proteins were detected by Co-IP and immunoblot analysis. The data underlying this Figure can be found in S1 Raw Images.
