# Supplementary material for: VANGL2 alleviates inflammatory bowel disease by recruiting the ubiquitin ligase MARCH8 to limit NLRP3 inflammasome activation through OPTN-mediated selective autophagy
Source: PLoS Biol. 2025 Feb 3;23(2):e3002961. doi: 10.1371/journal.pbio.3002961 (PMC11790156; doi:10.1371/journal.pbio.3002961)
Supplement: S5 Fig — (A) PEMs were silenced with Vangl2 siRNA for 24 h, followed by LPS (100 ng/ml) and CQ (50 μM) treatment for 6 h, and NLRP3 was pulled down by IP. Immunoblot analysis was used to detect the expression of Ub and NLRP3. (B) HKE293T cells were silenced with VANGL2 siRNA for 24 h, then transfected with HA-Ub-K27 and Flag-NLRP3 plasmids for 24 h, followed by CQ (50 μM) treatment for 6 h. Flag was pulled down by IP, and the expression of HA and Flag tagged proteins were detected by immunoblot analysis. (C–E) HKE293T cells were silenced with MARCH1 siRNA (C), MARCH3 siRNA (D), or MARCH11 siRNA (E) for 24 h, and then transfected with Flag-NLRP3 and Myc-VANGL2 plasmids for 24 h. Subsequently, the expression of Flag and Myc-tagged proteins were detected by immunoblot analysis. (F) HEK293T cells were silenced with MARCH7 siRNA for 24 h, followed by transfection with HA-Ub-K27, Flag-NLRP3, and Myc-VANGL2 plasmids for 24 h. Flag was then pulled down through IP, and the expression of HA, Flag, and Myc tagged proteins were detected by immunoblot analysis. (G) HEK293T cells were silenced with MARCH7 siRNA for 24 h, followed by transfection with Flag-NLRP3 and Myc-VANGL2 plasmids for 24 h. The expression of Flag, Myc, and MARCH7 proteins were detected by immunoblot analysis. (H) PEMs were silenced with March8 siRNA for 24 h, followed by LPS (100 ng/ml) treatment for 6 h, and finally the expression of NLRP3 was detected by immunoblot analysis. (I) PEMs were silenced with March8 siRNA for 24 h, followed by LPS (100 ng/ml) treatment for 6 h and ATP treatment for 30 min. The expression of IL-1β was detected by ELISA. Data are expressed as means ± SD. ***P < 0.001. (PDF) [file pbio.3002961.s005.pdf]

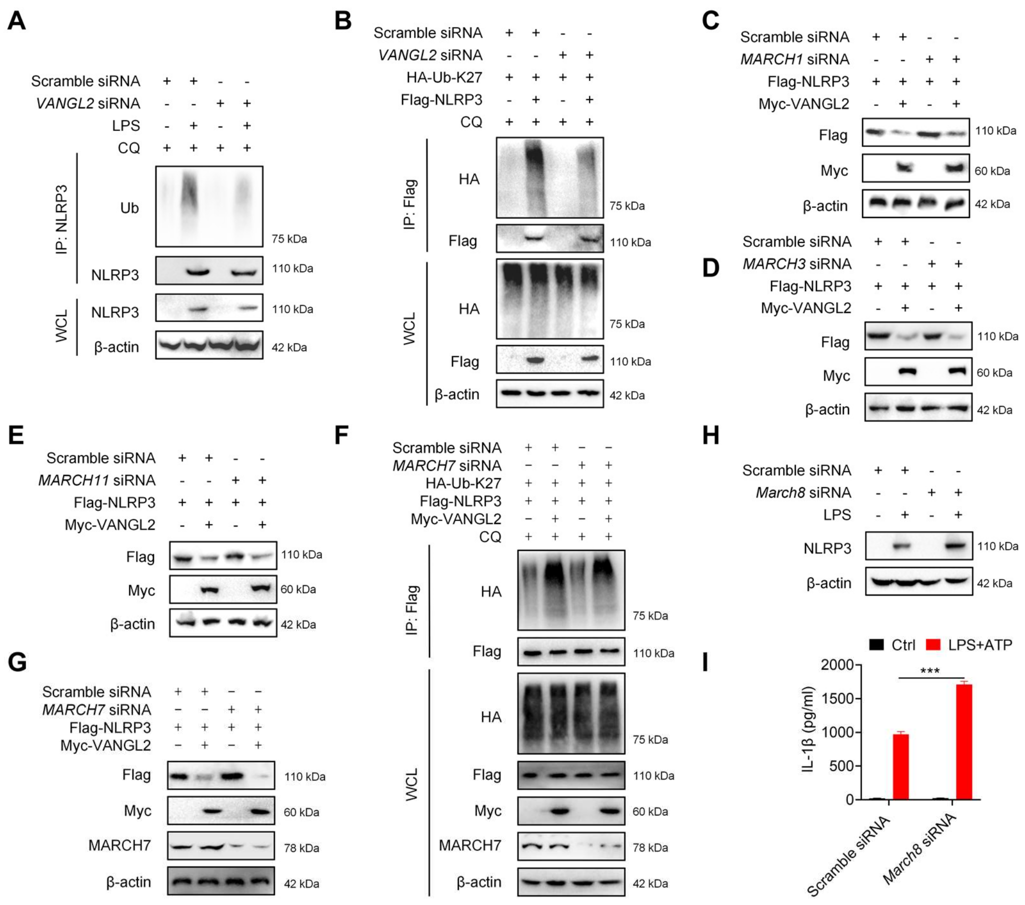

S5 Fig. VANGL2 recruits MARCH8 to promote the K27-linked ubiquitination of NLRP3.

(A) PEMs were silenced with *Vangl2* siRNA for 24 h, followed by LPS (100 ng/mL) and CQ (50 μM) treatment for 6 h, and NLRP3 was pulled down by IP. Immunoblot analysis was used to detect the expression of Ub and NLRP3. (B) HKE293T cells were silenced with *VANGL2* siRNA for 24 h, then transfected with HA-Ub-K27 and Flag-NLRP3 plasmids for 24 h, followed by CQ (50 μM) treatment for 6 h. Flag was pulled down by IP, and the expression of HA and Flag tagged proteins were detected by immunoblot analysis. (C-E) HKE293T cells were silenced with *MARCH1* siRNA (C), *MARCH3* siRNA (D), or *MARCH11* siRNA (E) for 24 h, and then transfected with Flag-NLRP3 and Myc-VANGL2 plasmids for 24 h. Subsequently, the expression of Flag and Myc-tagged proteins were detected by immunoblot analysis. (F) HEK293T cells were silenced with *MARCH7* siRNA for 24 h, followed by transfection with HA-Ub-K27, Flag-NLRP3, and Myc-VANGL2 plasmids for 24 h. Flag was then pulled

down through IP, and the expression of HA, Flag, and Myc tagged proteins were detected by immunoblot analysis. (G) HEK293T cells were silenced with *MARCH7* siRNA for 24 h, followed by transfection with Flag-NLRP3 and Myc-VANGL2 plasmids for 24 h. The expression of Flag, Myc, and MARCH7 proteins were detected by immunoblot analysis. (H) PEMs were silenced with *March8* siRNA for 24 h, followed by LPS (100 ng/mL) treatment for 6 h, and finally the expression of NLRP3 was detected by immunoblot analysis. (I) PEMs were silenced with *March8* siRNA for 24 h, followed by LPS (100 ng/mL) treatment for 6 h and ATP treatment for 30 min. The expression of IL-1β was detected by ELISA. Data are expressed as means ± SD. $^{***}P < 0.001$. The data underlying this Figure can be found in S1 Data and S1 Raw Images.
