# Supplementary material for: VANGL2 alleviates inflammatory bowel disease by recruiting the ubiquitin ligase MARCH8 to limit NLRP3 inflammasome activation through OPTN-mediated selective autophagy
Source: PLoS Biol. 2025 Feb 3;23(2):e3002961. doi: 10.1371/journal.pbio.3002961 (PMC11790156; doi:10.1371/journal.pbio.3002961)
Supplement: S6 Fig — (A) Flag-NLRP3 (WT and K823R) and GFP-LC3B plasmids were transfected into HEK293T cells for 24 h and then treated with CQ (50 μM) for 6 h. Flag was pulled down by IP, and the expression of GFP and Flag tagged proteins were detected by immunoblot analysis. (B) Purified VANGL2, NLRP3, MARCH8, and OPTN were detected by Coomassie brilliant blue staining. (C–H) The purified proteins were mixed in a co-IP buffer to perform an in vitro affinity-isolation assay, and the results were analyzed via immunoblot assay. (I) HEK293T cells were transfected with Flag-NLRP3, HA-VANGL2, GFP-MARCH8, and Myc-OPTN. A three-step co-immunoprecipitation assay was performed with the cell lysates. Flag immunoprecipitates, HA immunoprecipitates, and GFP immunoprecipitates were analyzed by immunoblotting. (PDF) [file pbio.3002961.s006.pdf]

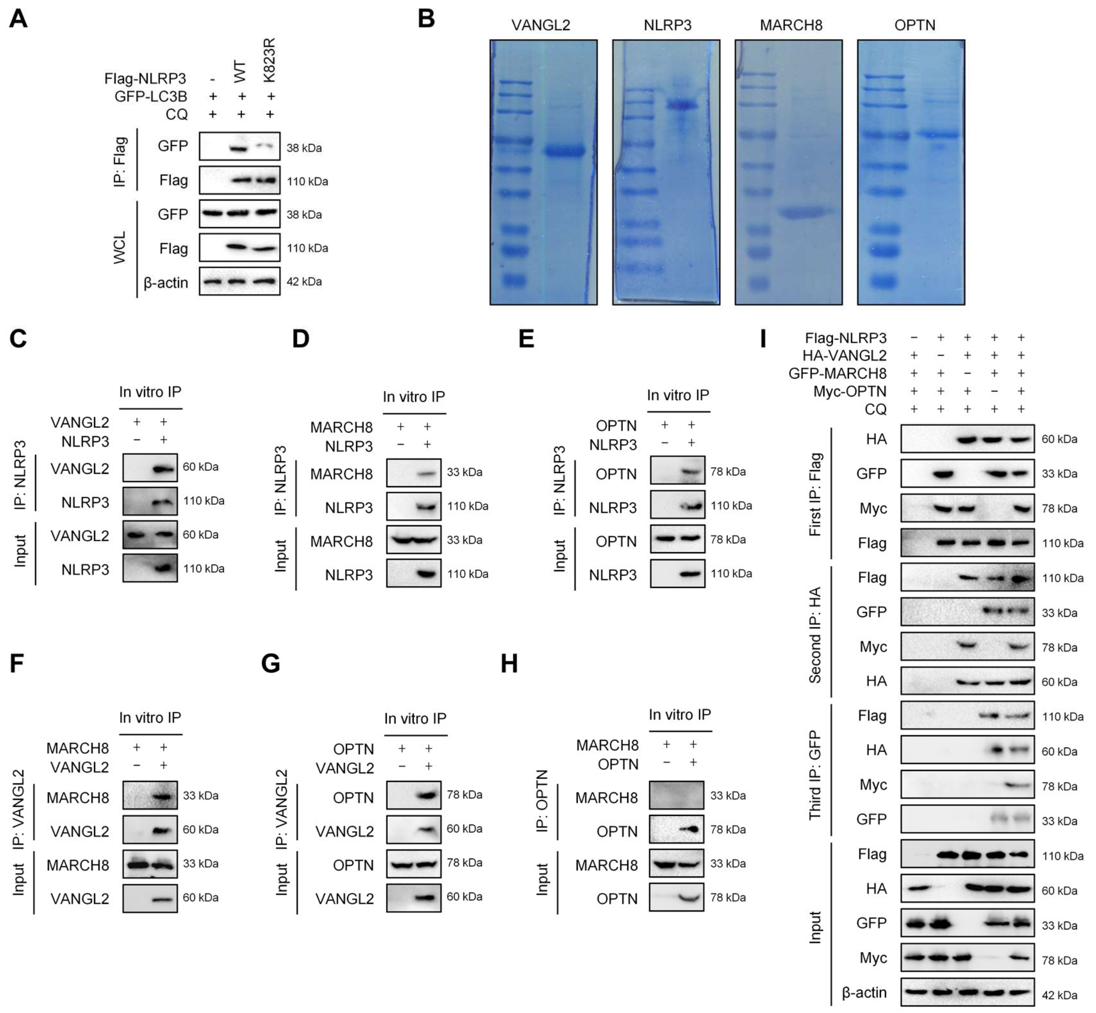

S6 Fig. VANGL2, NLRP3, MARCH8, and OPTN interact and form a complex.

(A) Flag-NLRP3 (WT and K823R) and GFP-LC3B plasmids were transfected into HEK293T cells for 24 h and then treated with CQ (50 μM) for 6 h. Flag was pulled down by IP, and the expression of GFP and Flag tagged proteins were detected by immunoblot analysis. (B) Purified VANGL2, NLRP3, MARCH8, and OPTN were detected by Coomassie Brilliant Blue staining. (C-H) The purified proteins were mixed in a co-IP buffer to perform an *in vitro* affinity-isolation assay, and the results were analyzed via immunoblot assay. (I) HEK293T cells were transfected with Flag-NLRP3, HA-VANGL2, GFP-MARCH8, and Myc-OPTN. A three-step co-immunoprecipitation assay was performed with the cell lysates. Flag immunoprecipitates, HA immunoprecipitates, and GFP immunoprecipitates were analyzed by immunoblotting. The data underlying this Figure can be found in S1 Raw Images.
