# Supplementary material for: VANGL2 alleviates inflammatory bowel disease by recruiting the ubiquitin ligase MARCH8 to limit NLRP3 inflammasome activation through OPTN-mediated selective autophagy
Source: PLoS Biol. 2025 Feb 3;23(2):e3002961. doi: 10.1371/journal.pbio.3002961 (PMC11790156; doi:10.1371/journal.pbio.3002961)
Supplement: S7 Fig — (A) Alcian blue staining was used to detect the intestinal characteristics of WT mice, Vangl2ΔM/ΔM mice, and MCC950 treated Vangl2ΔM/ΔM mice after DSS treatment. Scale bar = 100 μm. (B) Inflammasome-related plasmids (NLRP3, ASC, pro-Casp1, and IL-1β), HA-VANGL2, and GFP-MARCH8 (WT, W114A) plasmids were transfected into HEK293T cells (MARCH8 KO) for 24 h, and the expression of IL-1β was detected by ELISA. (C–I) The construction of March8-WT, March8-W110A, or Vangl2 overexpression lentiviral plasmids and virus packaging were used to infect March8 KO bone marrow (BM) cells according to the following 4 groups: March8-WT, March8-WT+Vangl2, March8-W110A, March8-W110A+Vangl2. Recipient mice aged 6–8 weeks were irradiated with 4.5 Gy, then irradiated again with 4 Gy 4 h later, and randomly divided into 4 groups to receive lentivirus-treated BM cells, respectively. Chimeric mice were kept in SPF condition and used for the construction of DSS-mediated colitis after 6–8 weeks. The survival rate (C), weight change (D), disease activity index (E), colon length (F and G), HE staining of colon (H), and colon IL-1β (I) were measured. Data are expressed as means ± SD. *P < 0.05, ***P < 0.01, ***P < 0.001, NS means not significant. (PDF) [file pbio.3002961.s007.pdf]

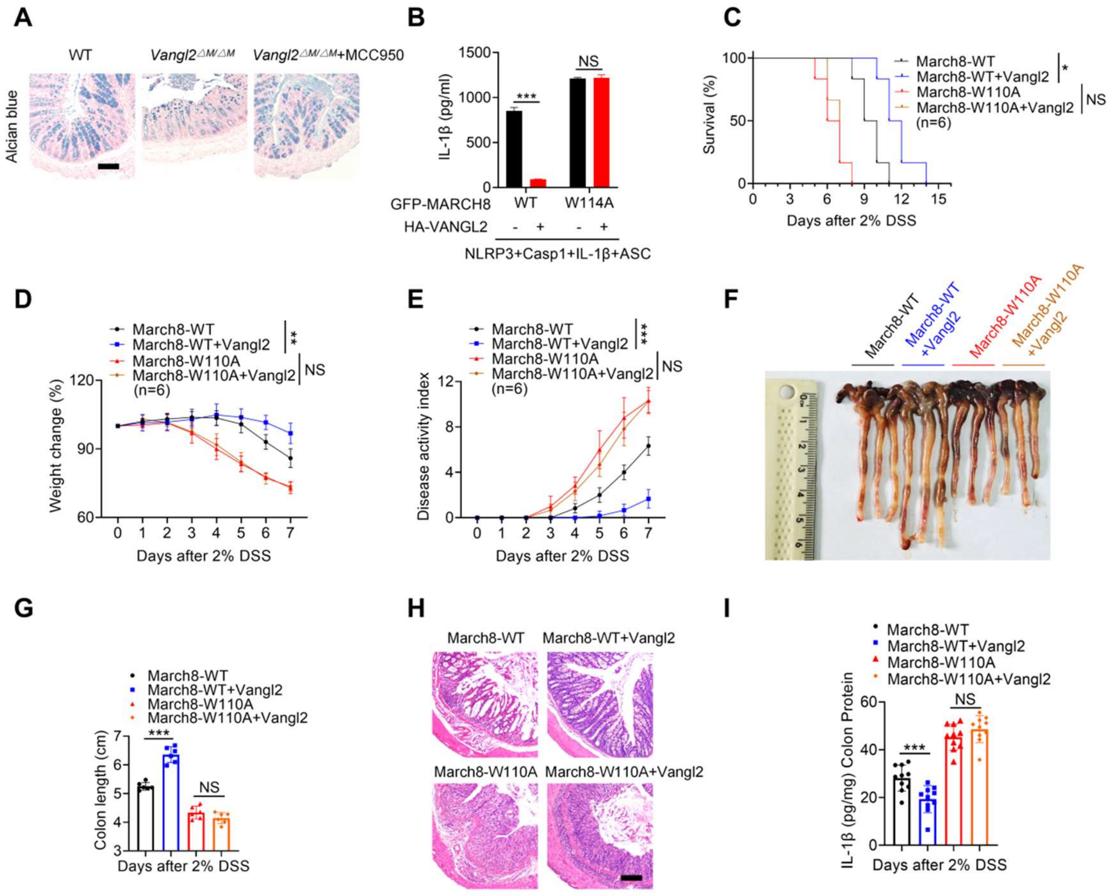

S7 Fig. VANGL2 inhibits NLRP3 inflammasome activation and DSS-induced colitis progression through the MARCH8 E3 enzyme activity dependent pathway.

(A) Alcian blue staining was used to detect the intestinal characteristics of WT mice, *Vangl2*$^{\Delta M/\Delta M}$ mice, and MCC950 treated *Vangl2*$^{\Delta M/\Delta M}$ mice after DSS treatment. Scale bar=100 μm. (B) Inflammasome-related plasmids (NLRP3, ASC, pro-Casp1, and IL-1β), HA-VANGL2, and GFP-MARCH8 (WT, W114A) plasmids were transfected into HEK293T cells (*MARCH8* KO) for 24 h, and the expression of IL-1β was detected by ELISA. (C-I) The construction of March8-WT, March8-W110A or Vangl2 overexpression lentiviral plasmids and virus packaging were used to infect March8 KO bone marrow (BM) cells according to the following four groups: March8-WT, March8-WT+Vangl2, March8-W110A, March8-W110A+Vangl2. Recipient mice aged 6-8 weeks were irradiated with 4.5 Gy，then irradiated again with 4 Gy 4 h later, and randomly divided into four groups to receive lentivirus-treated BM cells, respectively.

Chimeric mice were kept in SPF condition and used for the construction of DSS-mediated colitis after 6-8 weeks. The survival rate (C), weight change (D), disease activity index (E), colon length (F and G), HE staining of colon (H), and colon IL-1β (I) were measured. Data are expressed as means ± SD. $^{*}P < 0.05$, $^{***}P < 0.01$, $^{***}P < 0.001$, NS means not significant. The data underlying this Figure can be found in S1 Data.
