# Supplementary material for: VANGL2 alleviates inflammatory bowel disease by recruiting the ubiquitin ligase MARCH8 to limit NLRP3 inflammasome activation through OPTN-mediated selective autophagy
Source: PLoS Biol. 2025 Feb 3;23(2):e3002961. doi: 10.1371/journal.pbio.3002961 (PMC11790156; doi:10.1371/journal.pbio.3002961)
Supplement: S8 Fig — (A) THP-1 cells were transfected with scramble siRNA or VANGL2 siRNA for 24 h, followed by LPS (100 ng/ml) for 6 h and ATP (5 mM) for 30 min. Culture supernatants were collected to measure IL-1β release by ELISA. (B) THP-1 cells were transfected with scramble siRNA or MARCH8 siRNA for 24 h, followed by LPS (100 ng/ml) for 6 h and ATP (5 mM) for 30 min. Culture supernatants were collected to measure IL-1β release by ELISA. ***P < 0.001, NS means not significant. (PDF) [file pbio.3002961.s008.pdf]

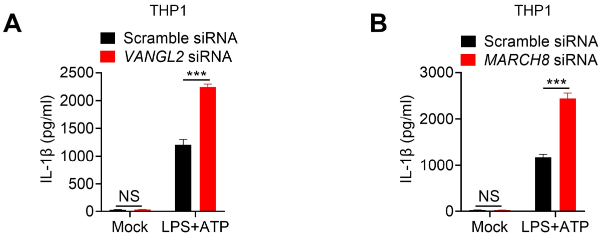

S8 Fig. VANGL2 and MARCH8 inhibit the activation of NLRP3 inflammasome in THP-1 cells.

(A) THP-1 cells were transfected with scramble siRNA or *VANGL2* siRNA for 24 h, followed by LPS (100 ng/mL) for 6 h and ATP (5 mM) for 30 min. Culture supernatants were collected to measure IL-1β release by ELISA. (B) THP-1 cells were transfected with scramble siRNA or *MARCH8* siRNA for 24 h, followed by LPS (100 ng/mL) for 6 h and ATP (5 mM) for 30 min. Culture supernatants were collected to measure IL-1β release by ELISA. $^{***}P < 0.001$, NS means not significant. The data underlying this Figure can be found in S1 Data.
