# Supplementary material for: VANGL2 alleviates inflammatory bowel disease by recruiting the ubiquitin ligase MARCH8 to limit NLRP3 inflammasome activation through OPTN-mediated selective autophagy
Source: PLoS Biol. 2025 Feb 3;23(2):e3002961. doi: 10.1371/journal.pbio.3002961 (PMC11790156; doi:10.1371/journal.pbio.3002961)
Supplement: S1 Table — (DOCX) [file pbio.3002961.s009.docx]

**Table 1. Primers and siRNA sequences used in this study.**

| **Primer sequences for qPCR in human** | |
| --- | --- |
| **Names** | **Sequences (5’ → 3’)** |
| *VANGL2*-F | AATCCCGAAAAGAAGGCTGT |
| *VANGL2*-R | CCCTATTCCCCACAACACAC |
| *NLRP3*-F | TGGATGGGTTTGCTGGGAT |
| *NLRP3*-R | CTGCGTGTAGCGACTGTTGAG |
| *GAPDH*-F | GCTGGTCATCAACGGGAAA |
| *GAPDH*-R | ACGCCAGTAGACTCCACGACA |
| **Primer sequences for qPCR in mouse** | |
| *Vangl2*-F | TGAGGGCCTCTTCATCTCC |
| *Vangl2*-R | GCCCGTGGAGTTAATTGGT |
| *Gapdh*-F | AAGGTCATCCCAGAGCTGAA |
| *Gapdh*-R | CTGCTTCACCACCTTCTTGA |
| **siRNA sequences** | |
| *VANGL2* siRNA (Human) | GTCACAATGAGTACTACTA |
| *MARCH1* siRNA (Human) | TGGATAAAGAGCTCAGATACACG |
| *MARCH3* siRNA (Human) | GCCATTAGCAGAGTAATTGCTGT |
| *MARCH8* siRNA (Human) | TCGTCAGTTTGACCAAGTATTCC |
| *MARCH11* siRNA (Human) | TGCTATGGAATGTATGGTTTTAT |
| *Vangl2* siRNA (Mouse) | UCCCAAGUCACACAAGUUUTT |
| *March8* siRNA (Mouse) | TACAATAGAGTGATCTATGTTCA |
