# Supplementary material for: VANGL2 alleviates inflammatory bowel disease by recruiting the ubiquitin ligase MARCH8 to limit NLRP3 inflammasome activation through OPTN-mediated selective autophagy
Source: PLoS Biol. 2025 Feb 3;23(2):e3002961. doi: 10.1371/journal.pbio.3002961 (PMC11790156; doi:10.1371/journal.pbio.3002961)
Supplement: S1 Raw Images — (PDF) [file pbio.3002961.s011.pdf]

Figure 1B

VANGL2

60 kDa

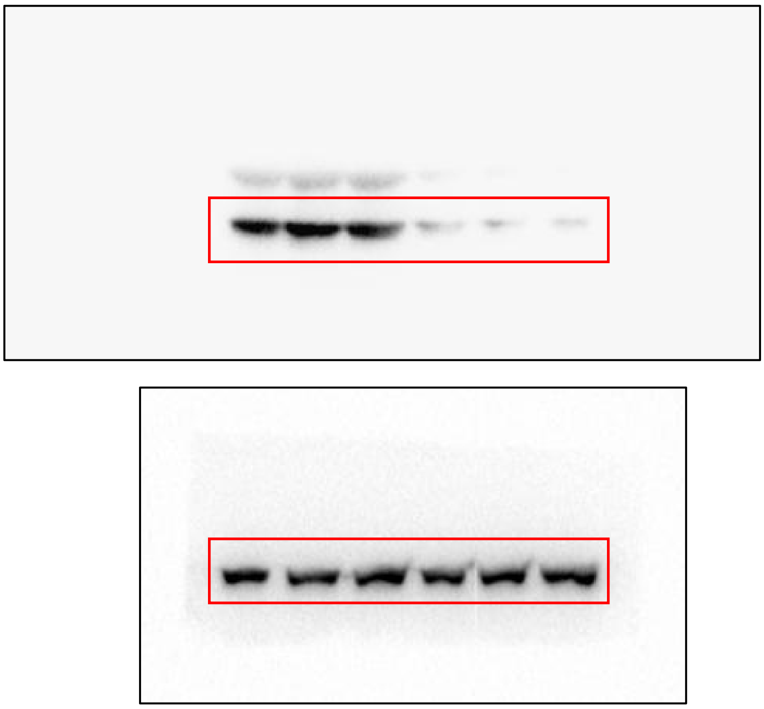

β-actin

42 kDa

Figure S1B

VANGL2

60 kDa

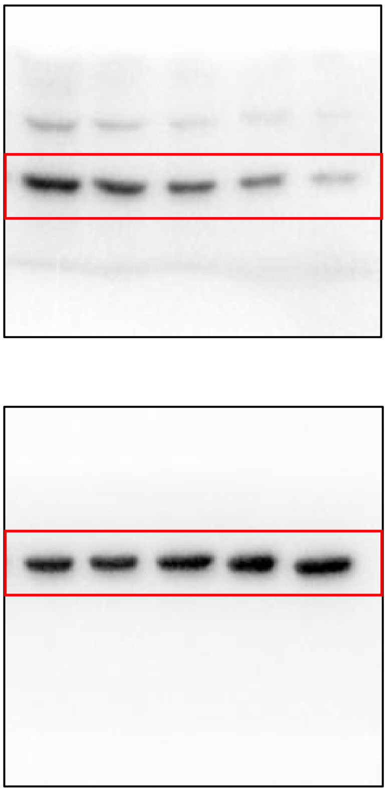

β-actin

42 kDa

Figure S1C

flox

WT

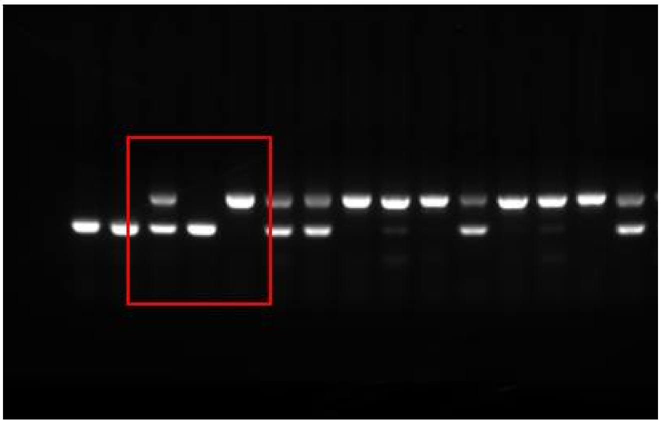

500bp

150bp

Lyz-Cre

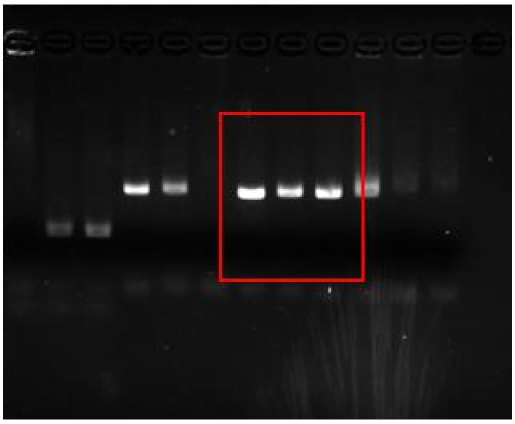

500bp

Figure S1D

VANGL2

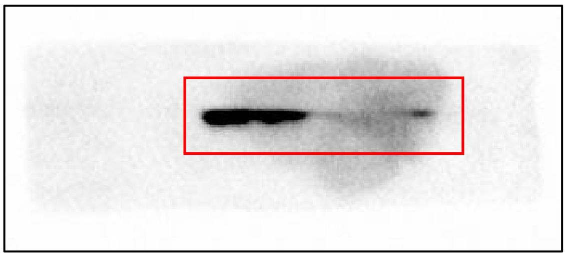

60 kDa

β-actin

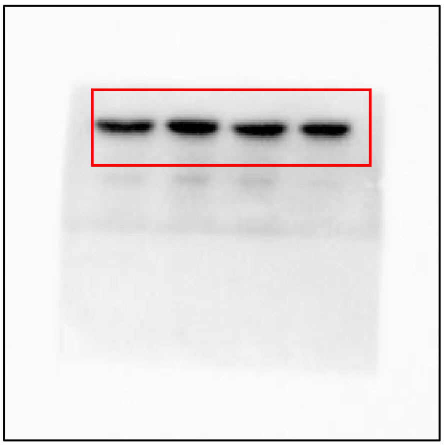

42 kDa

Figure S1G

VANGL2

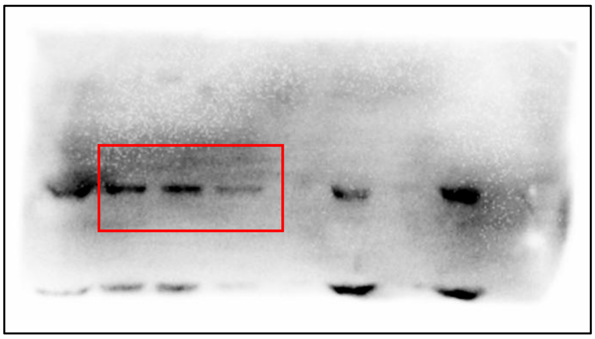

60 kDa

β-actin

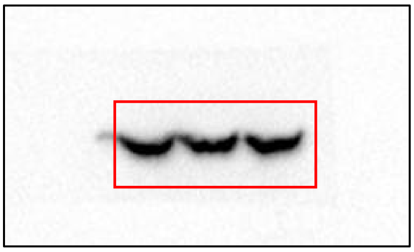

42 kDa

Figure 2D

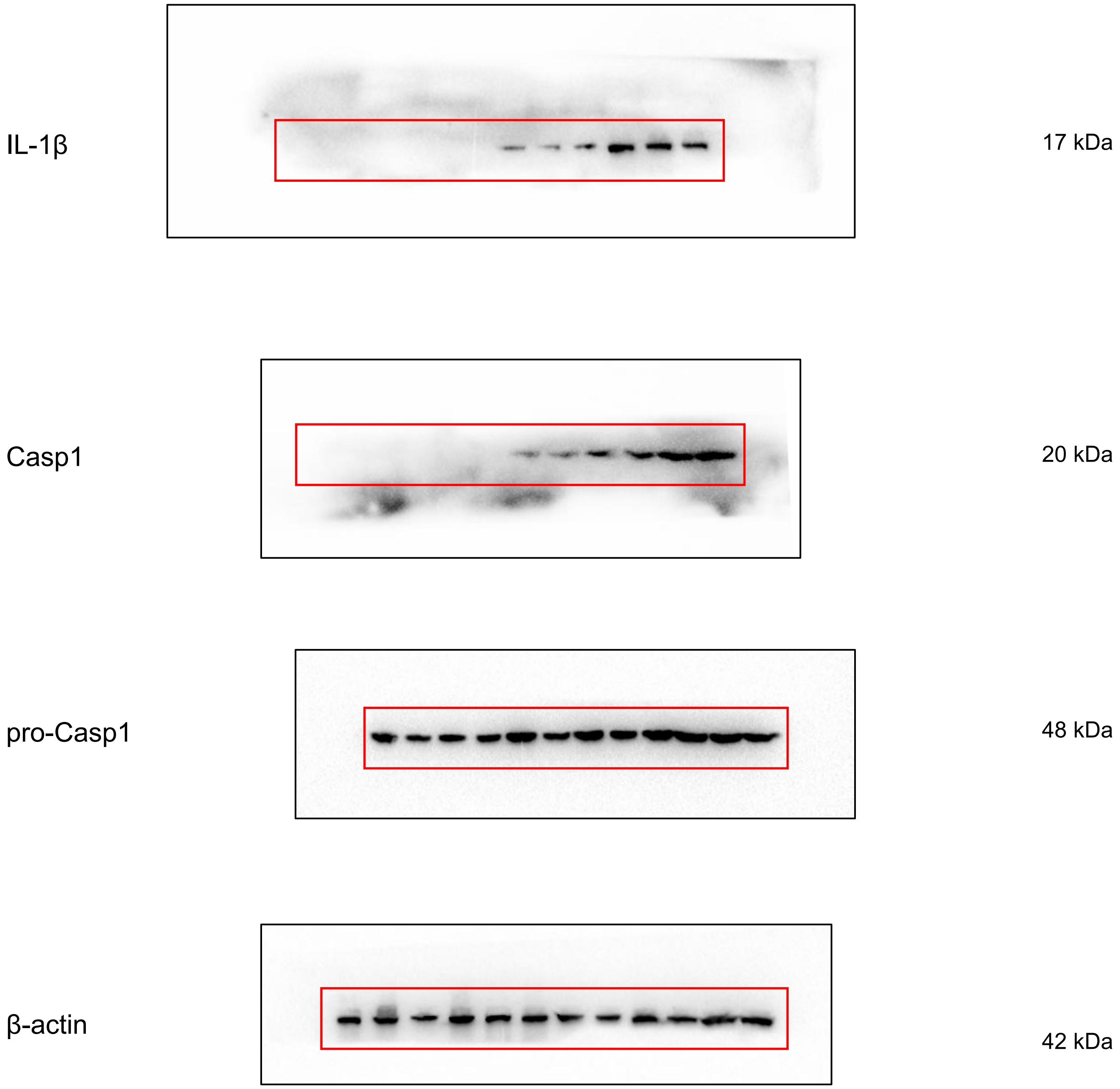

Figure 2G

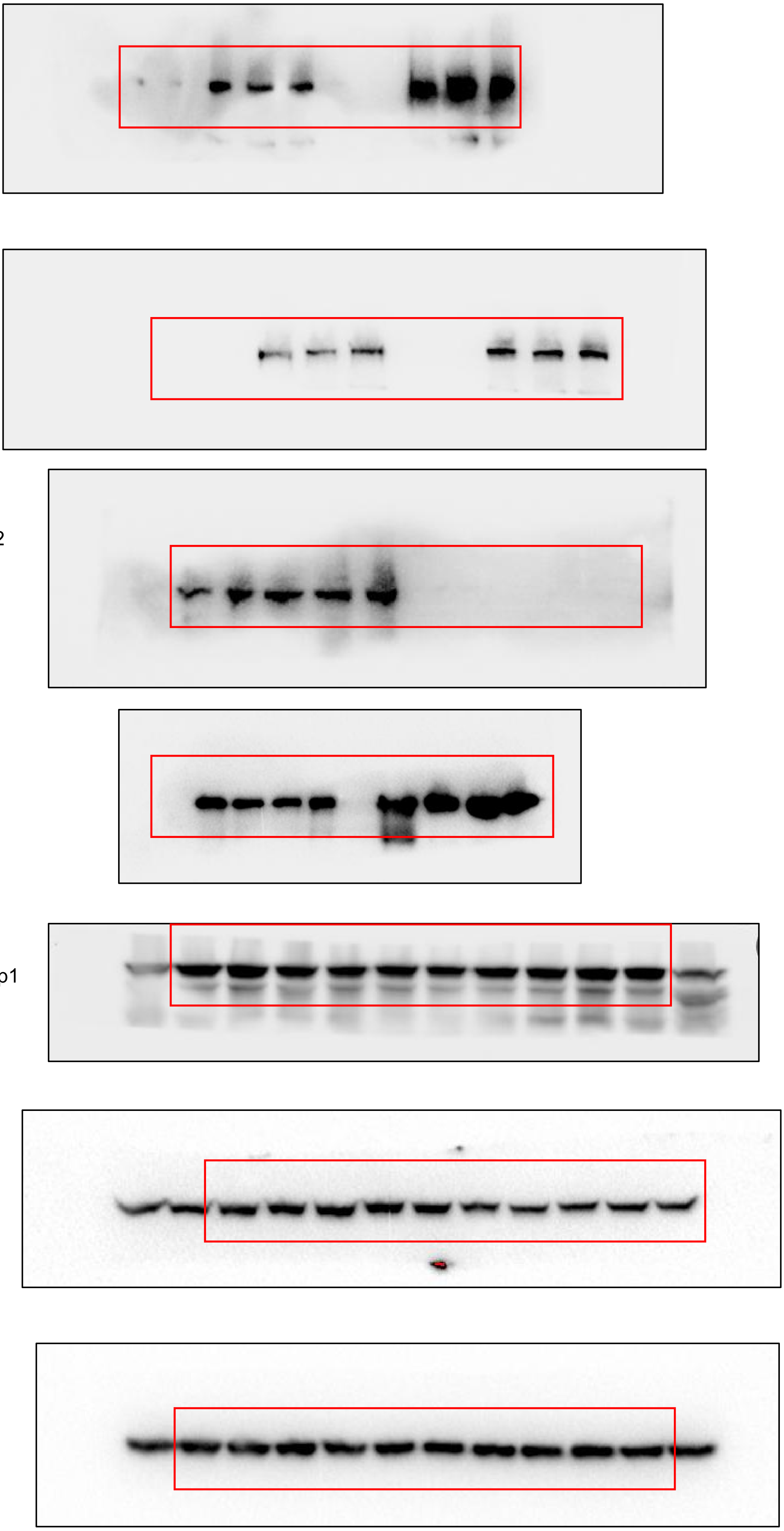

SN: IL-1β — 17 kDa

SN: Casp1 — 20 kDa

WCL: VANGL2 — 60 kDa

WCL: NLRP3 — 110 kDa

WCL: pro-Casp1 — 48 kDa

WCL: ASC — 22 kDa

WCL: β-actin — 42 kDa

Figure 2I

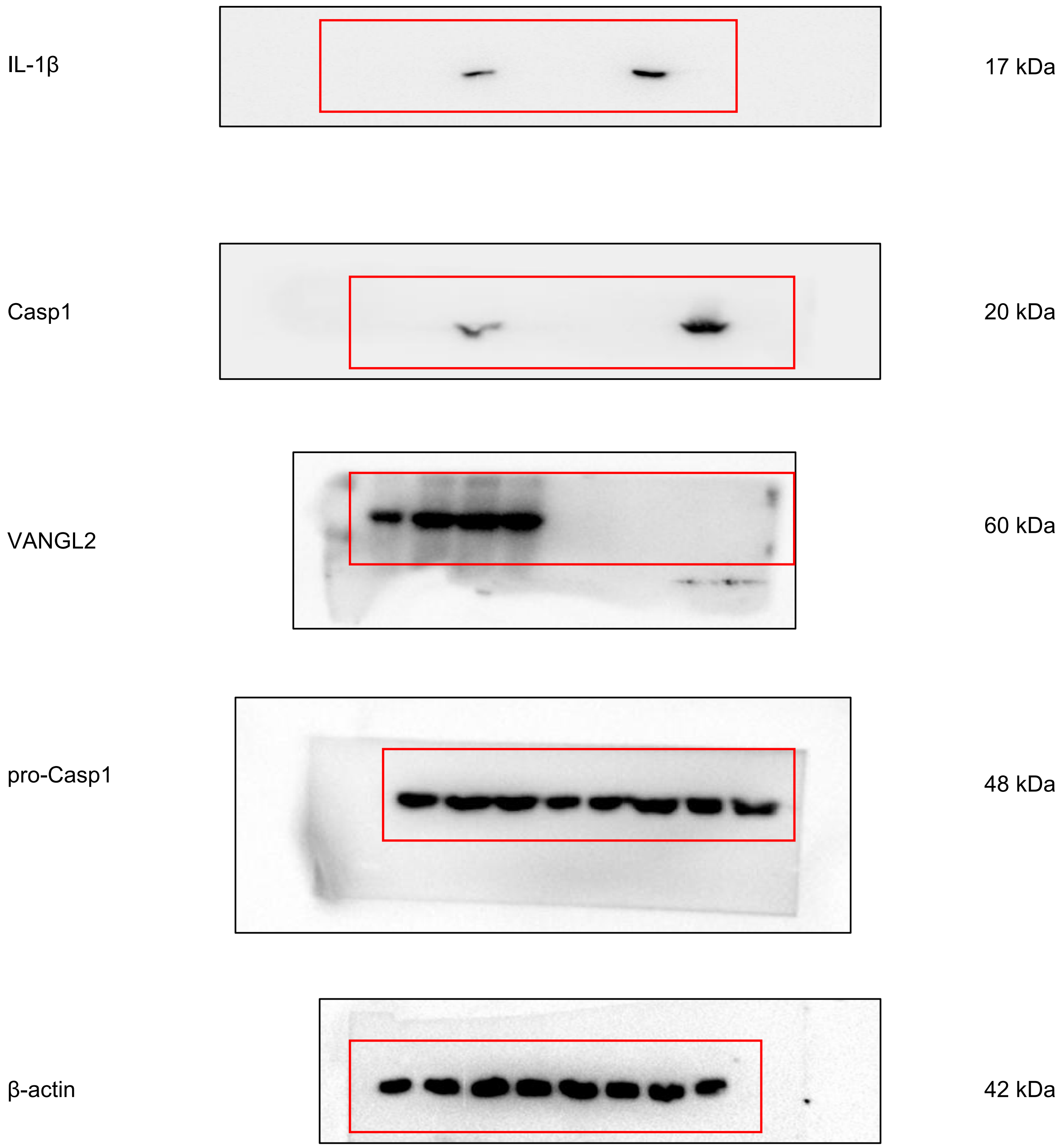
IL-1β
17 kDa
Casp1
20 kDa
VANGL2
60 kDa
pro-Casp1
48 kDa
β-actin
42 kDa

Figure 2K

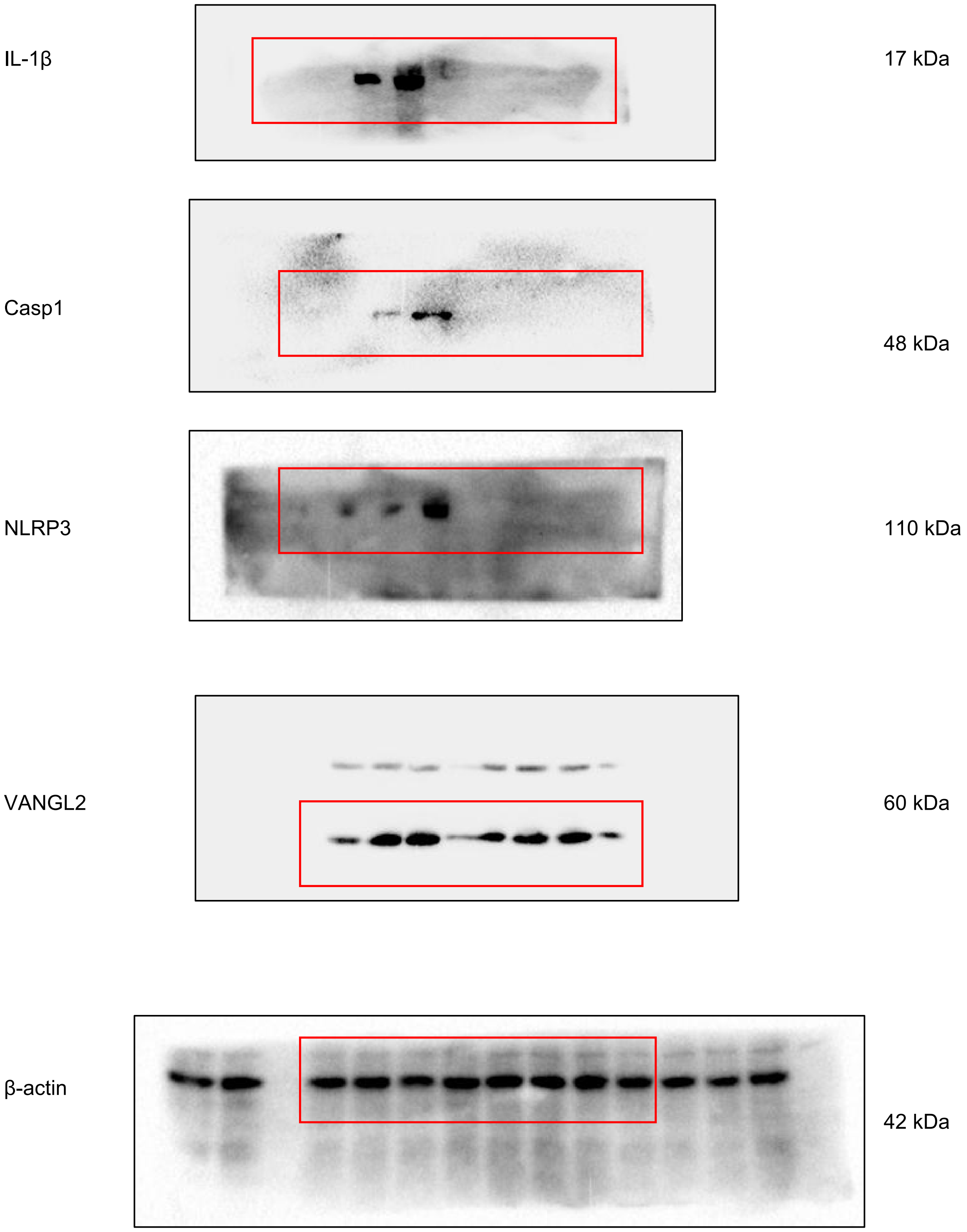
IL-1β
17 kDa
Casp1
48 kDa
NLRP3
110 kDa
VANGL2
60 kDa
β-actin
42 kDa

Figure S2D

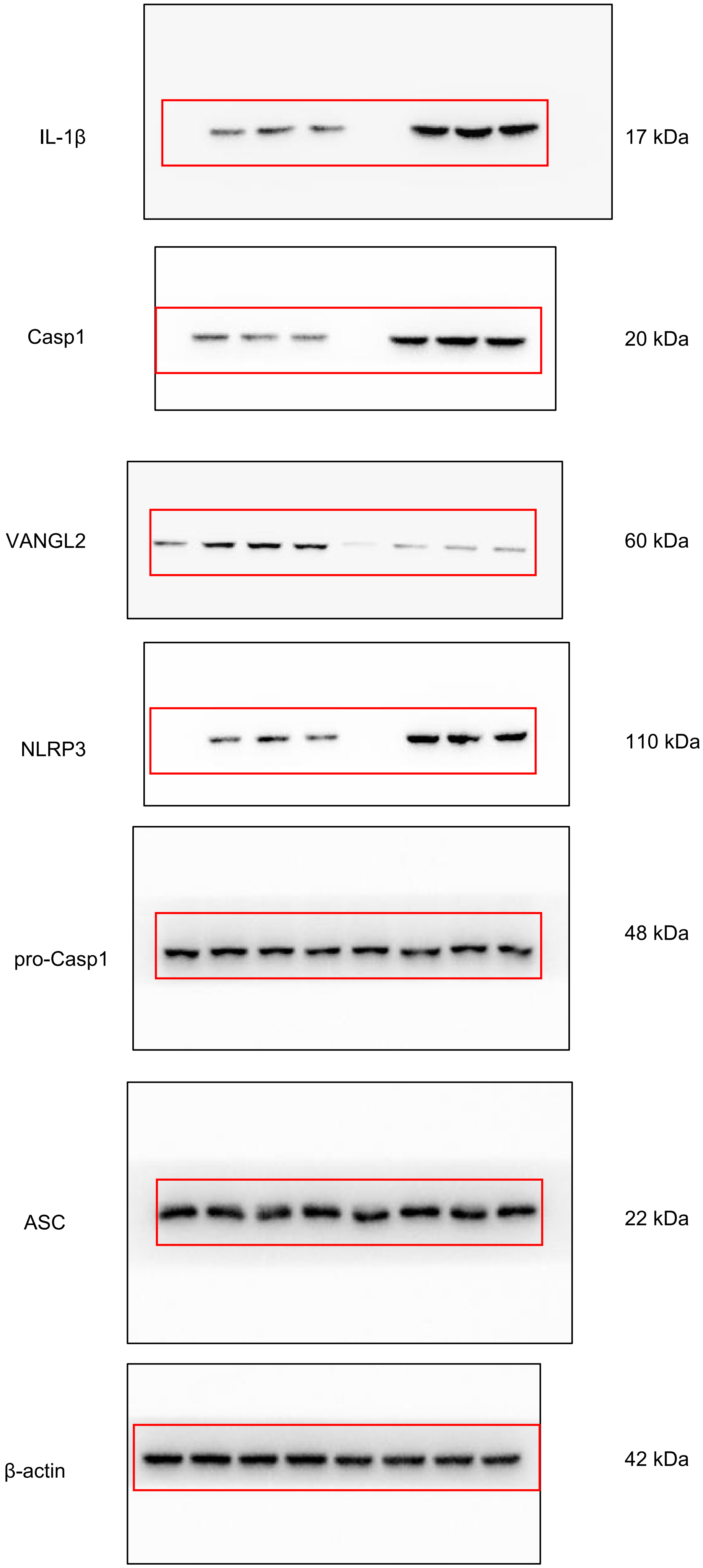
IL-1β
17 kDa
Casp1
20 kDa
VANGL2
60 kDa
NLRP3
110 kDa
pro-Casp1
48 kDa
ASC
22 kDa
β-actin
42 kDa

Figure S2A

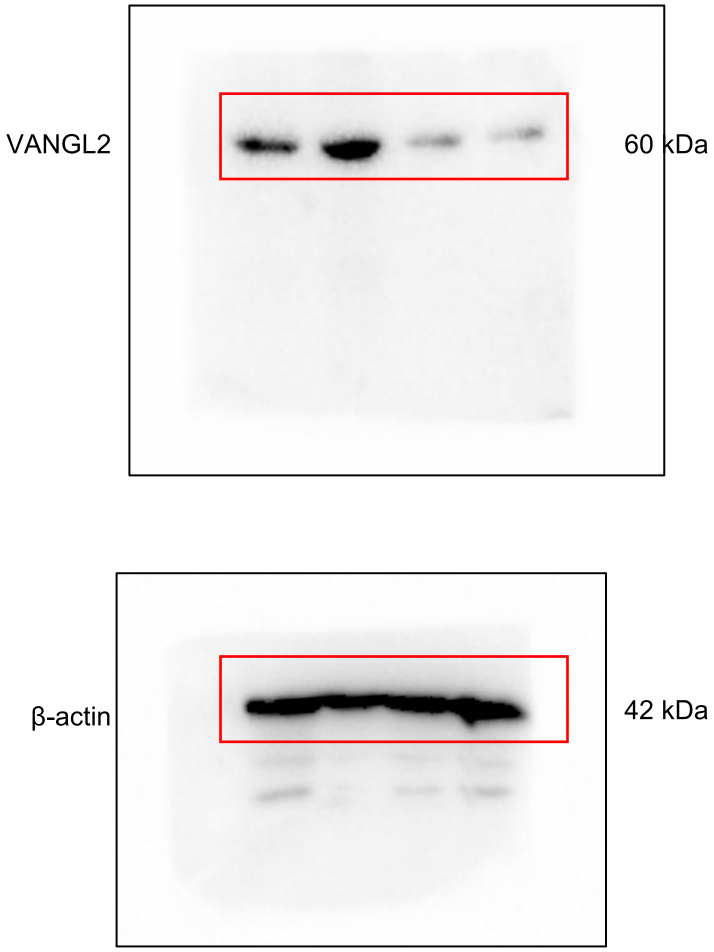

Figure S2E

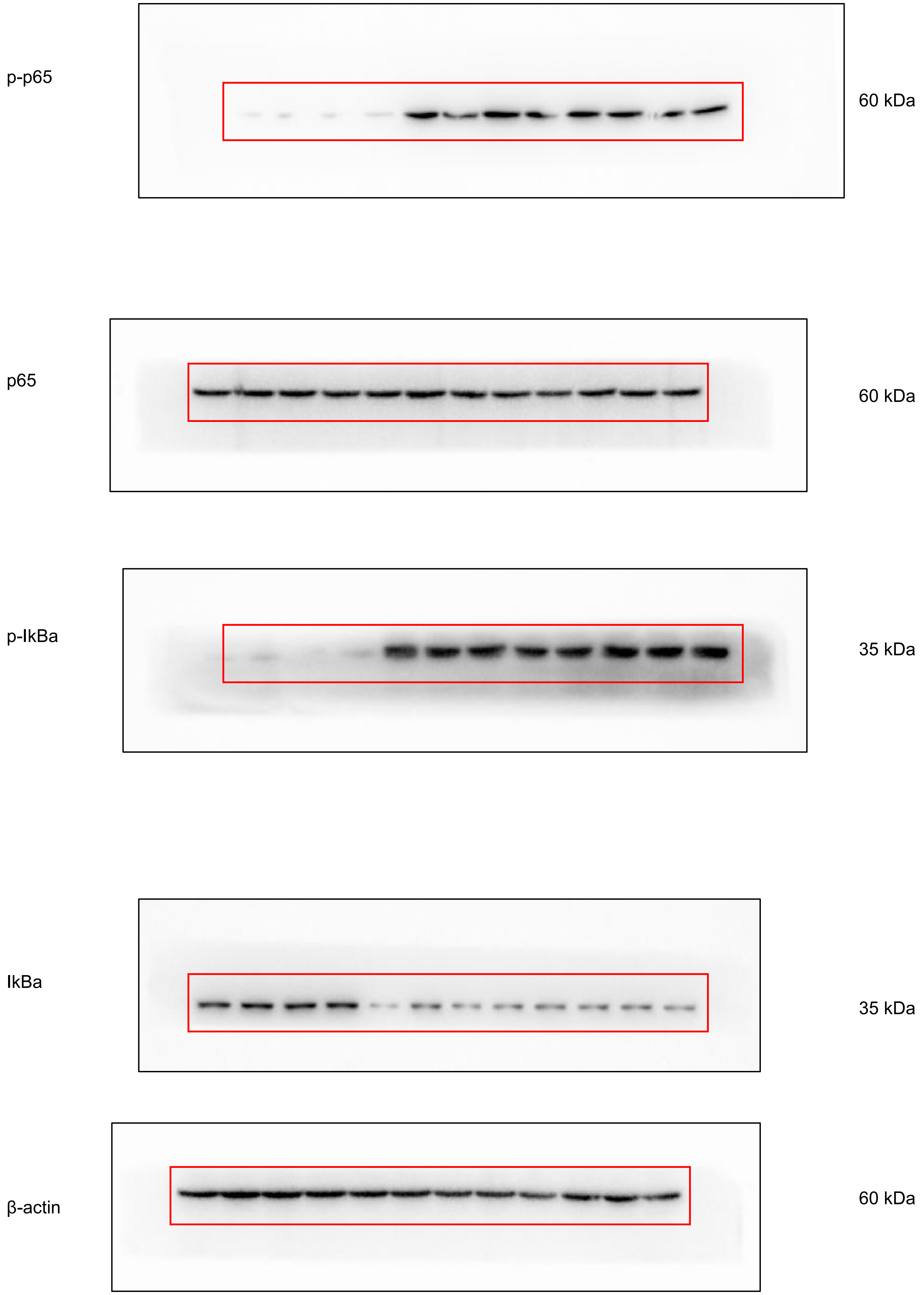

Figure 3A

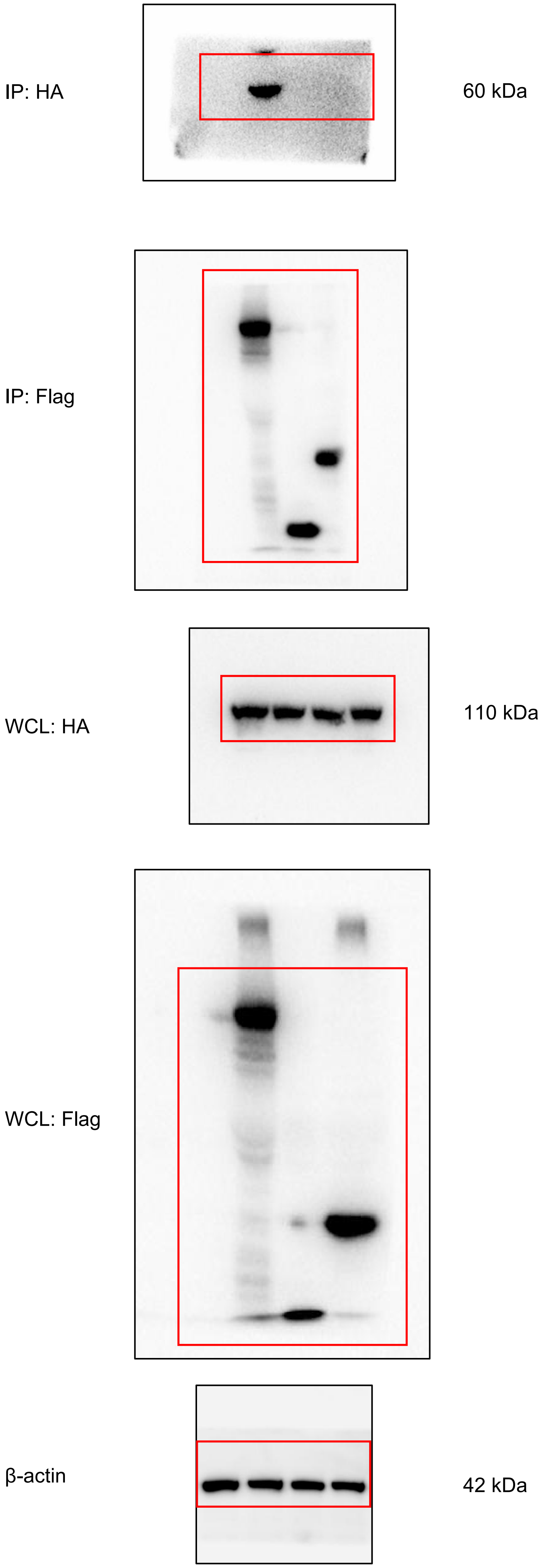

Figure 3B

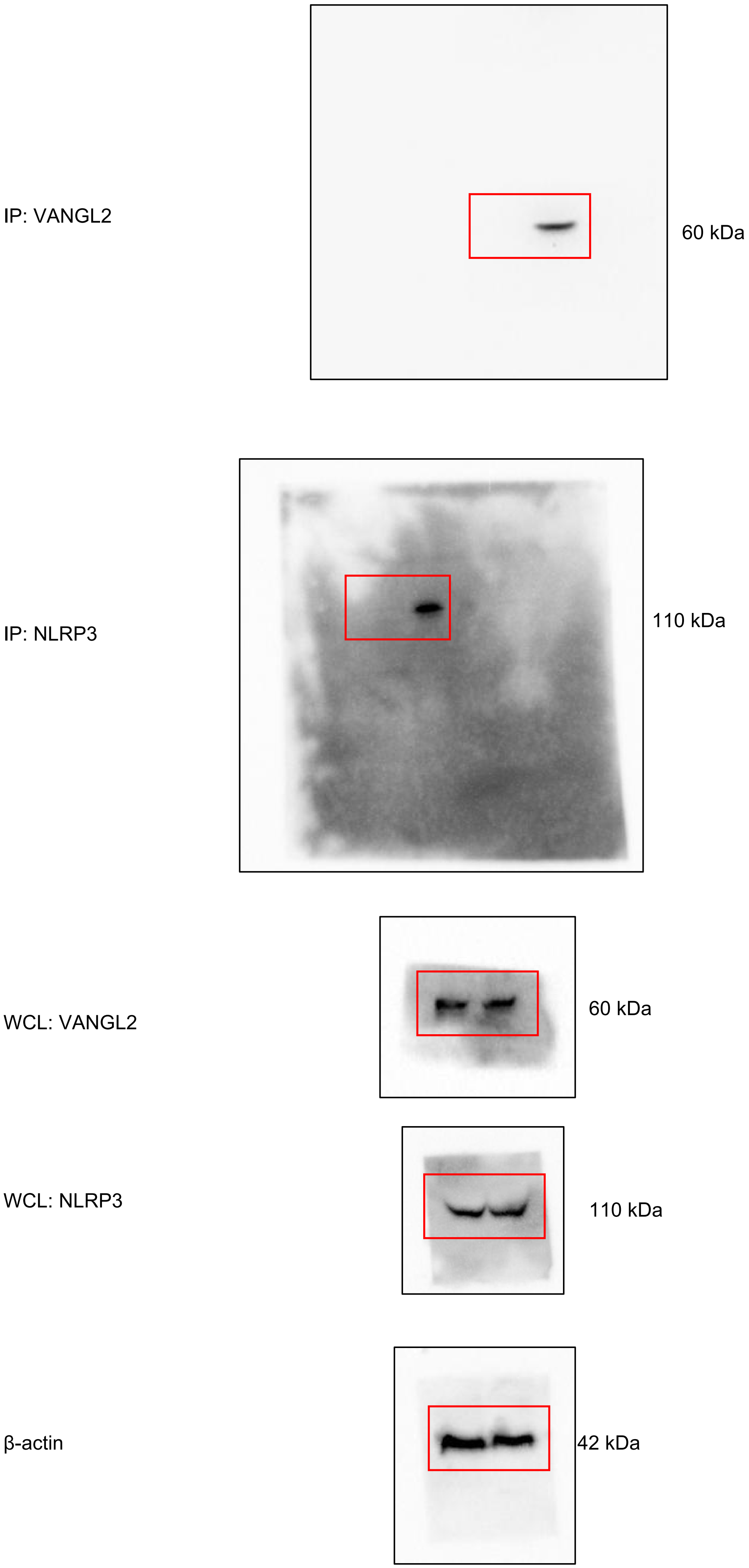
IP: VANGL2
60 kDa
IP: NLRP3
110 kDa
WCL: VANGL2
60 kDa
WCL: NLRP3
110 kDa
β-actin
42 kDa

Figure 3C

IP: NLRP3

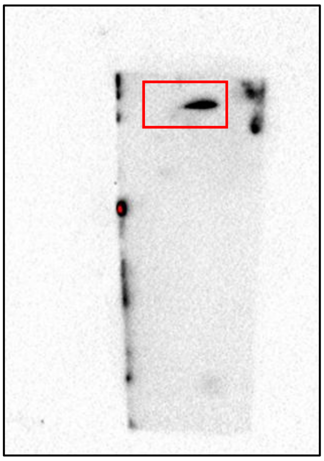

110 kDa

IP: VANLG2

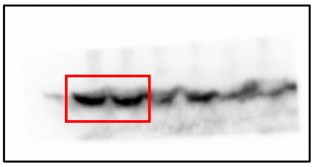

60 kDa

WCL: NLRP3

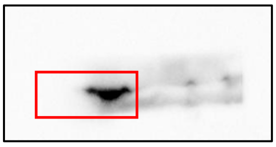

110 kDa

WCL: VANLG2

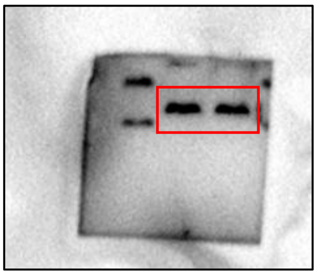

60 kDa

Figure 3E

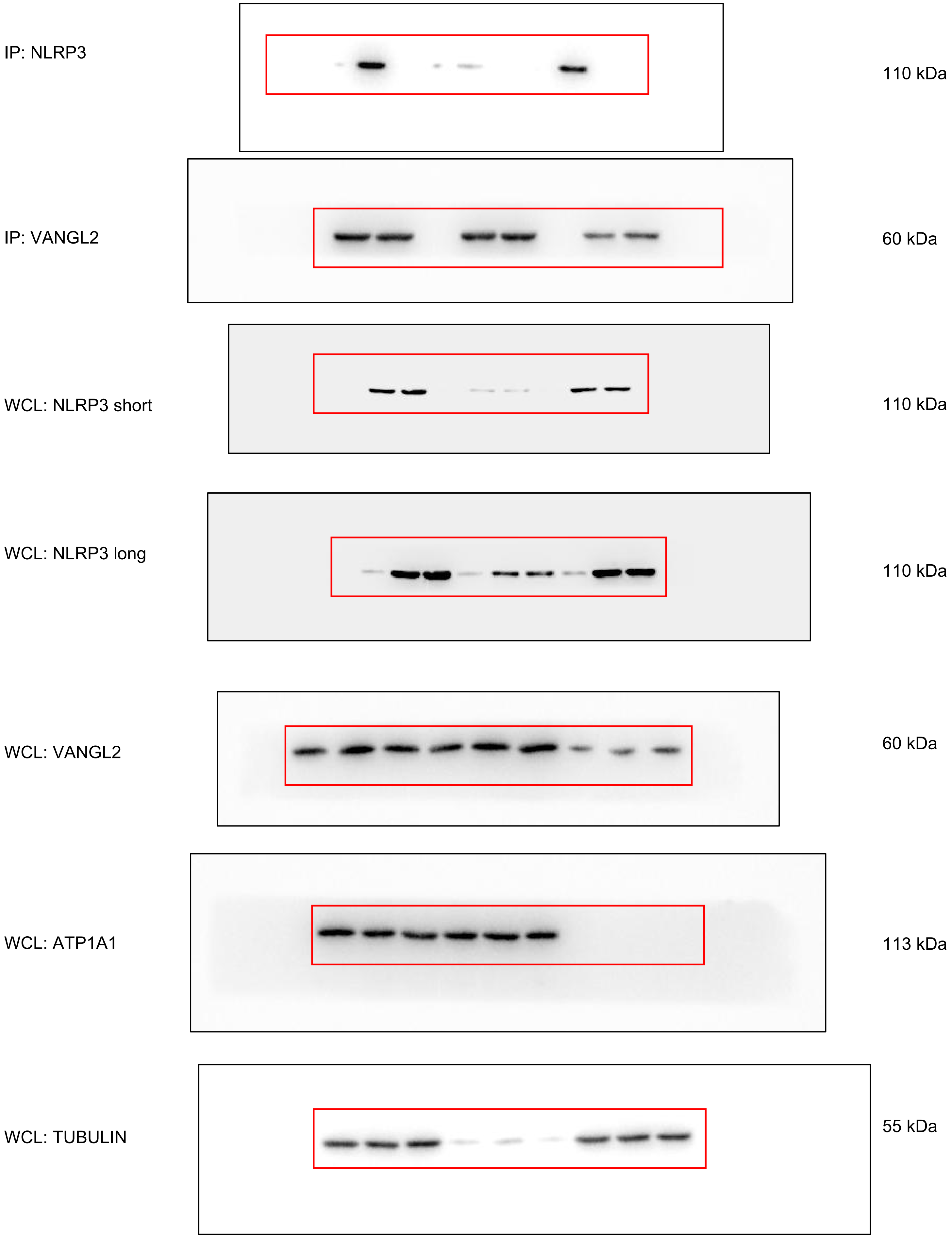
IP: NLRP3
110 kDa
IP: VANGL2
60 kDa
WCL: NLRP3 short
110 kDa
WCL: NLRP3 long
110 kDa
WCL: VANGL2
60 kDa
WCL: ATP1A1
113 kDa
WCL: TUBULIN
55 kDa

Figure 3G

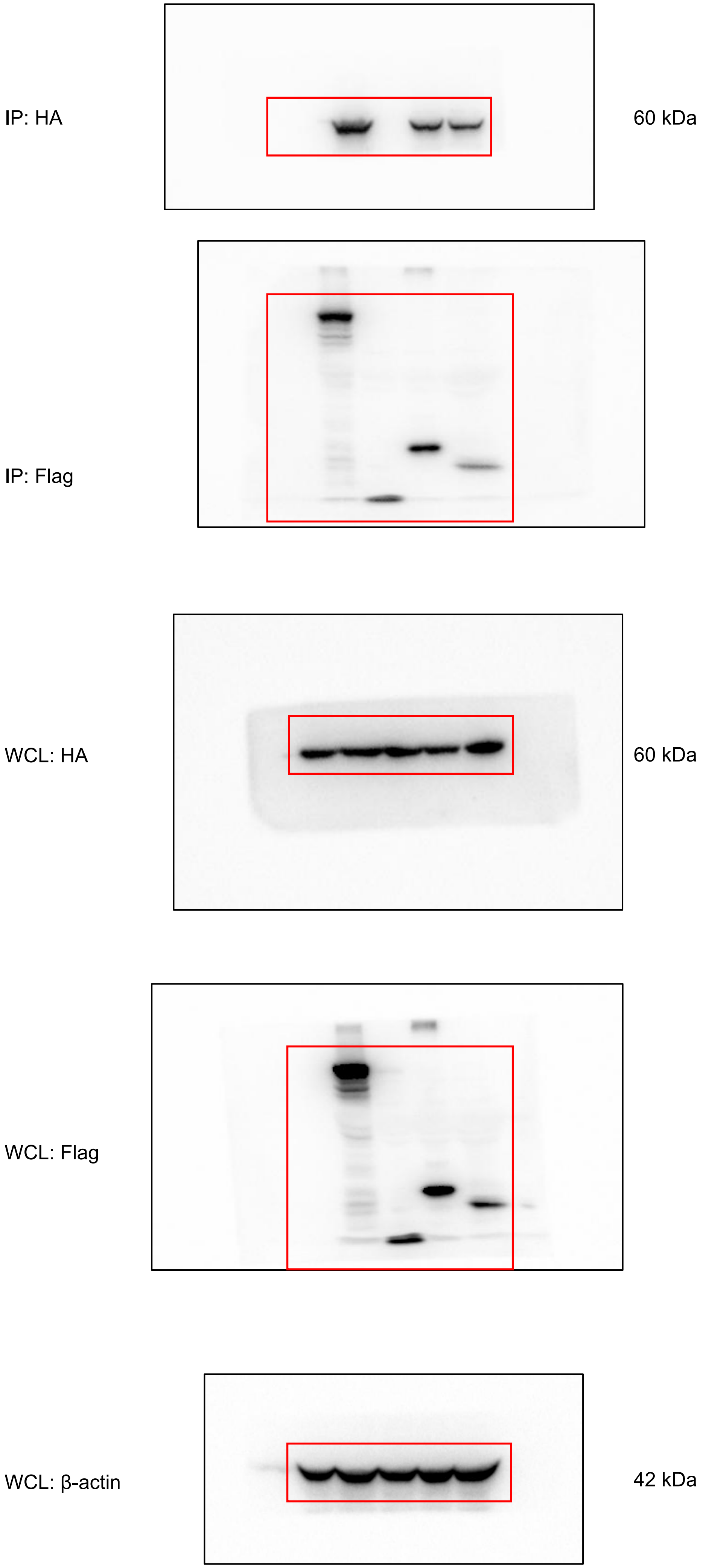

Figure 3I

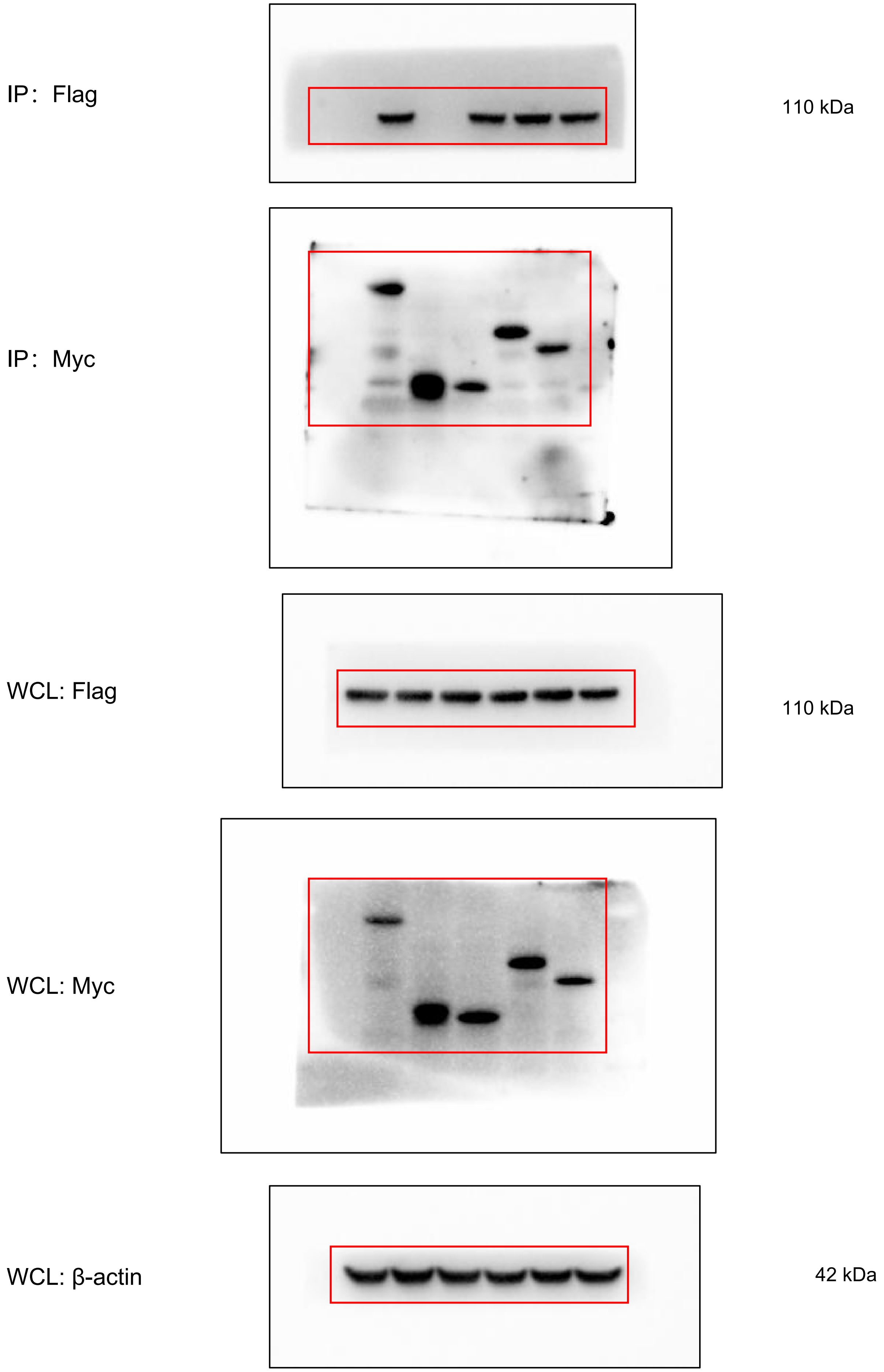

IP: Flag
110 kDa
IP: Myc
WCL: Flag
110 kDa
WCL: Myc
WCL: β-actin
42 kDa

Figure 3J

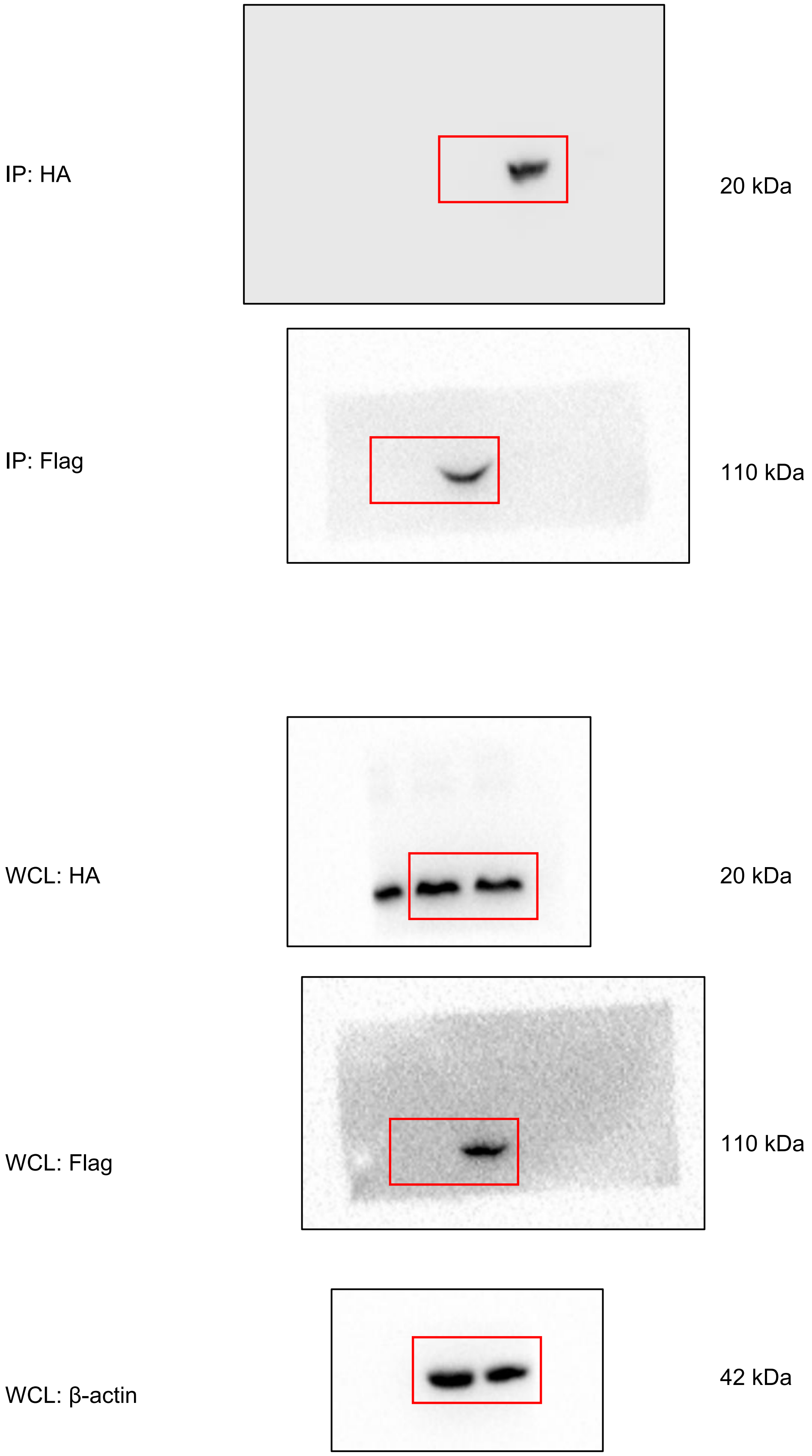
IP: HA
20 kDa
IP: Flag
110 kDa
WCL: HA
20 kDa
WCL: Flag
110 kDa
WCL: β-actin
42 kDa

Figure S3A

IP: HA

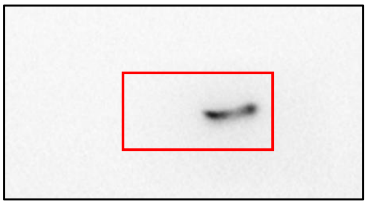

110 kDa

IP: Flag

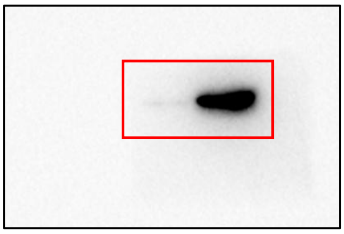

60 kDa

WCL: HA

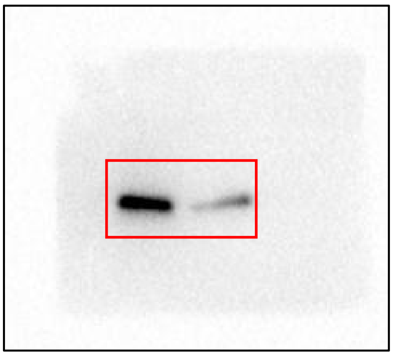

110 kDa

WCL: Flag

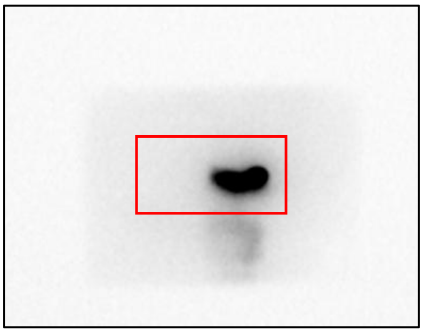

60 kDa

WCL: β-actin

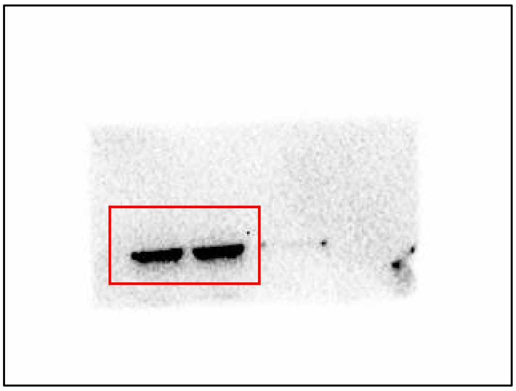

42 kDa

Figure S3B

IP: NLRP3

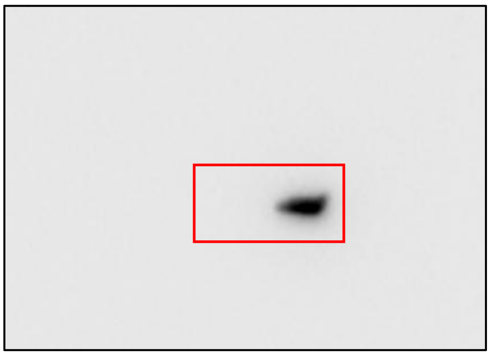

110 kDa

IP: VANGL2

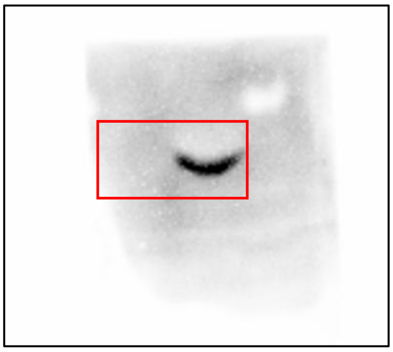

60 kDa

WCL: NLRP3

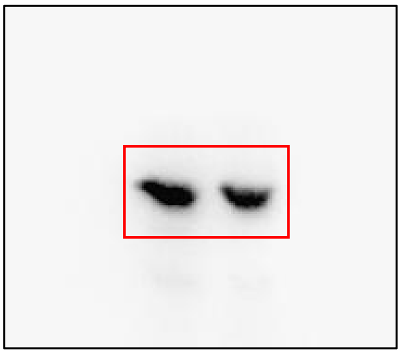

110 kDa

WCL: VANGL2

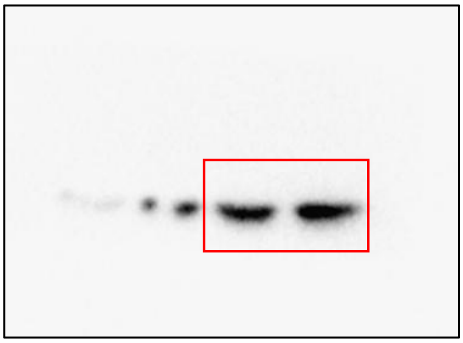

60 kDa

WCL: β-actin

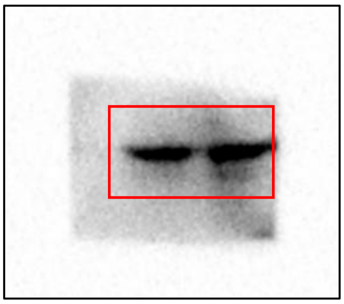

42 kDa

Figure S3D

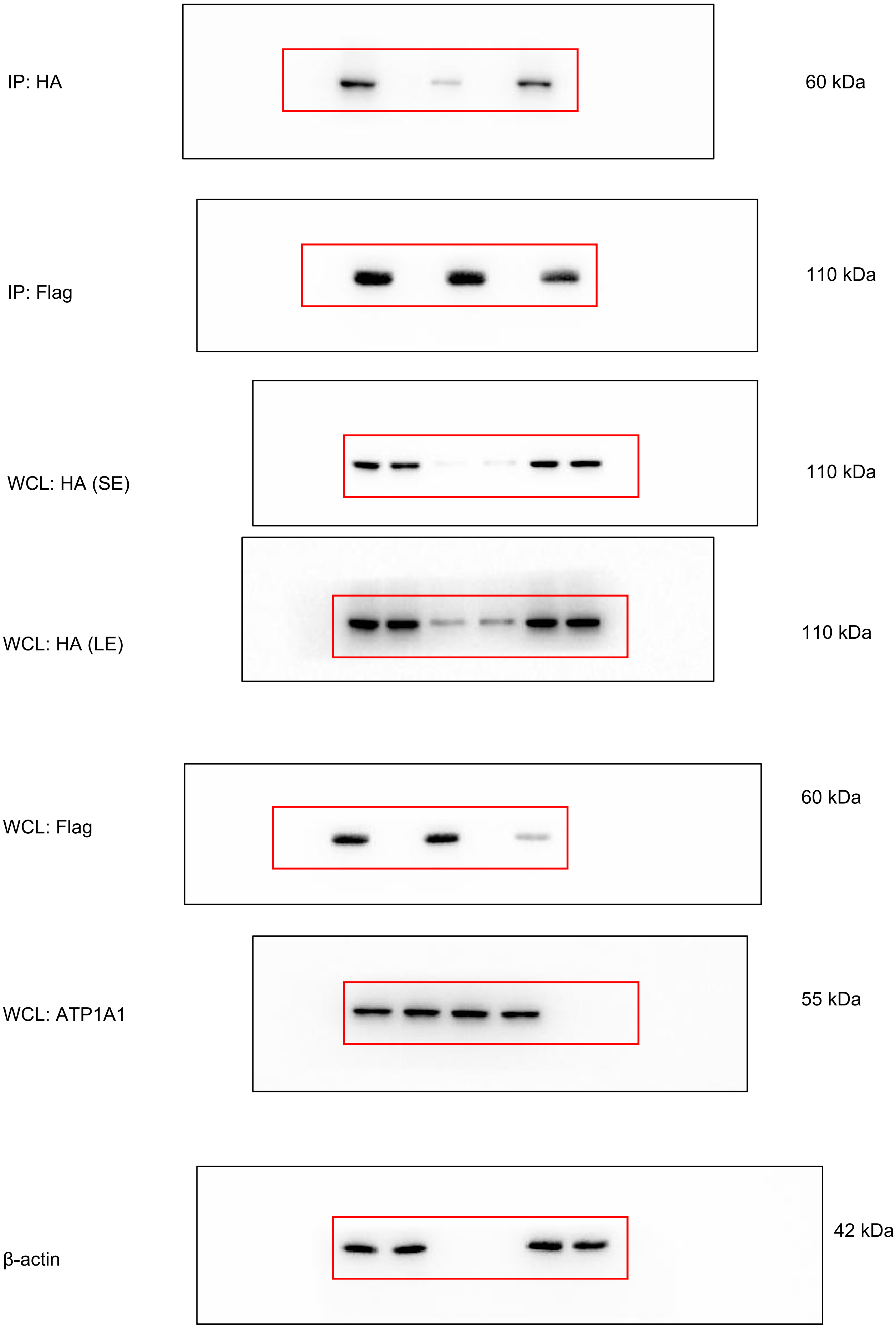
IP: HA
60 kDa
IP: Flag
110 kDa
WCL: HA (SE)
110 kDa
WCL: HA (LE)
110 kDa
WCL: Flag
60 kDa
WCL: ATP1A1
55 kDa
β-actin
42 kDa

Figure S3E

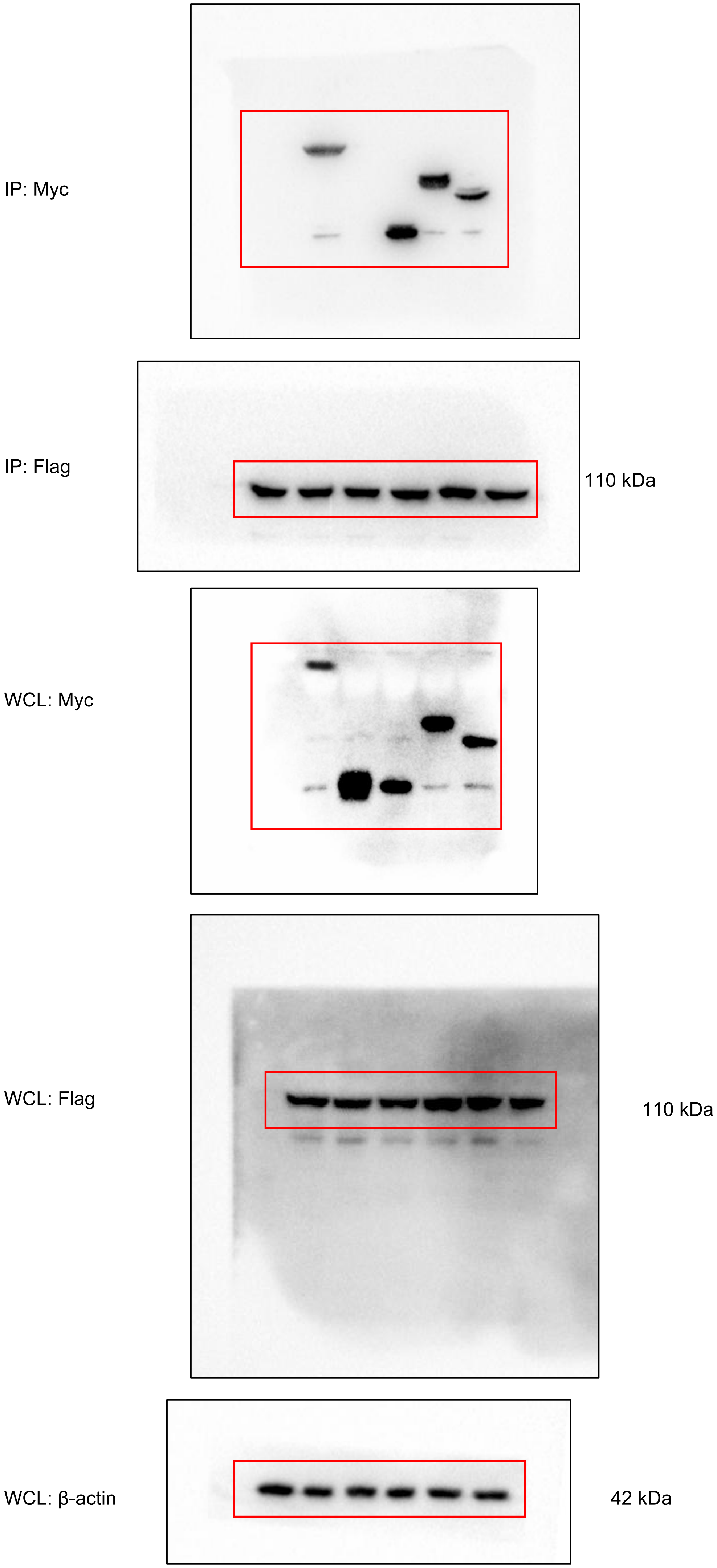
IP: Myc
IP: Flag
110 kDa
WCL: Myc
WCL: Flag
110 kDa
WCL: β-actin
42 kDa

Figure 4A

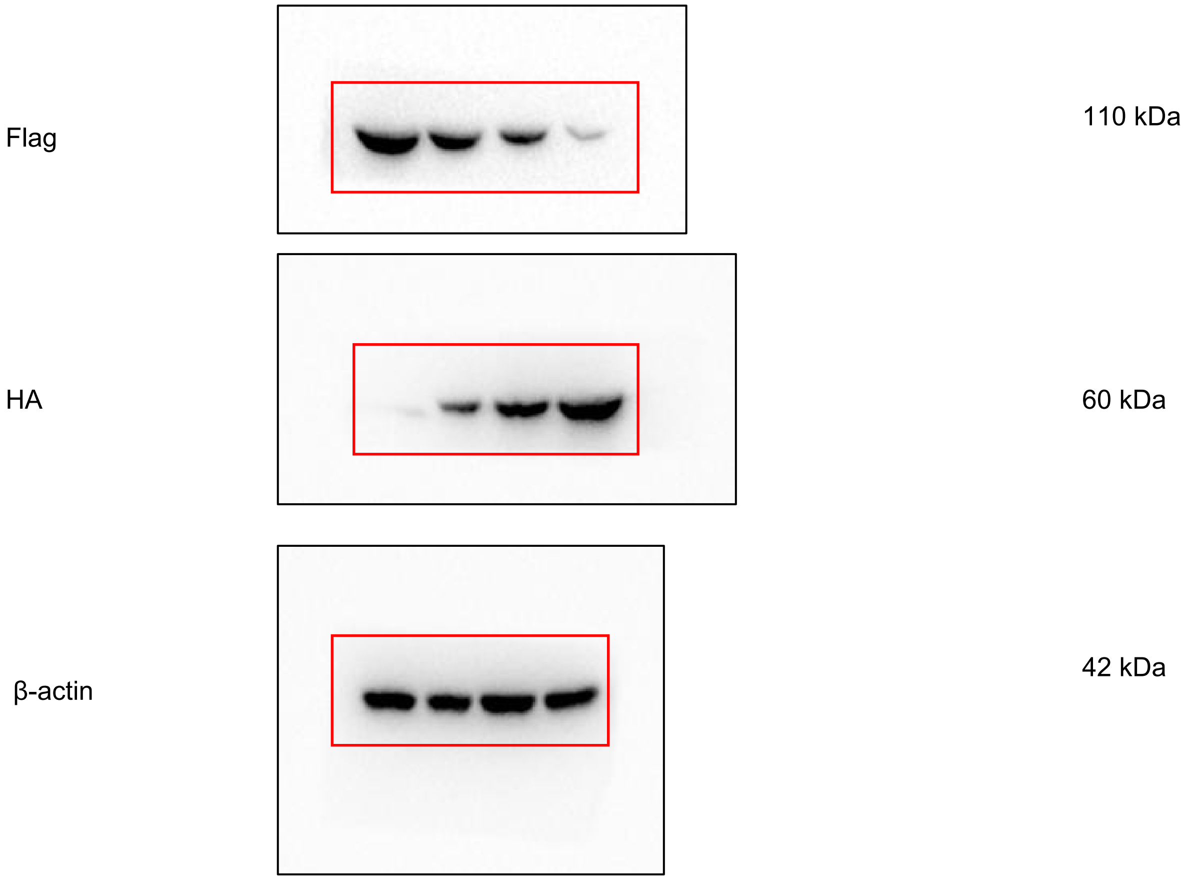

Figure 4C

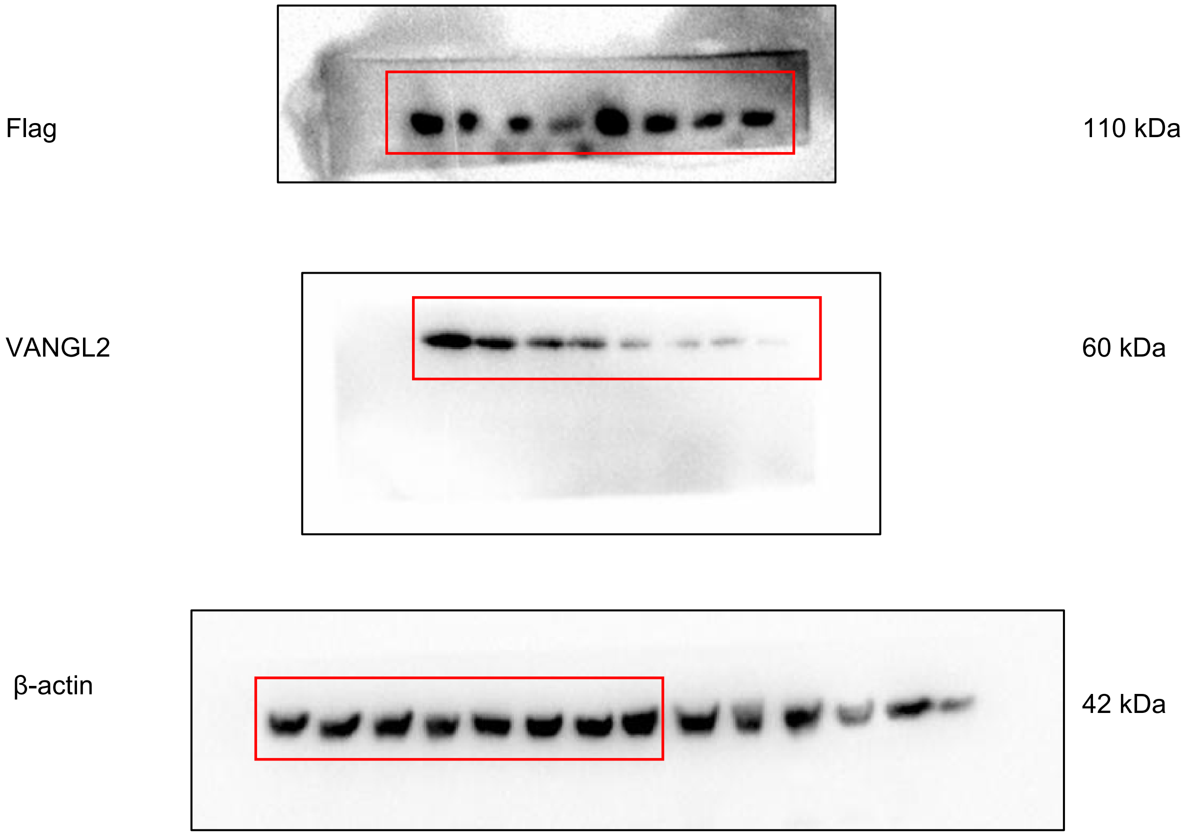

Figure 4E

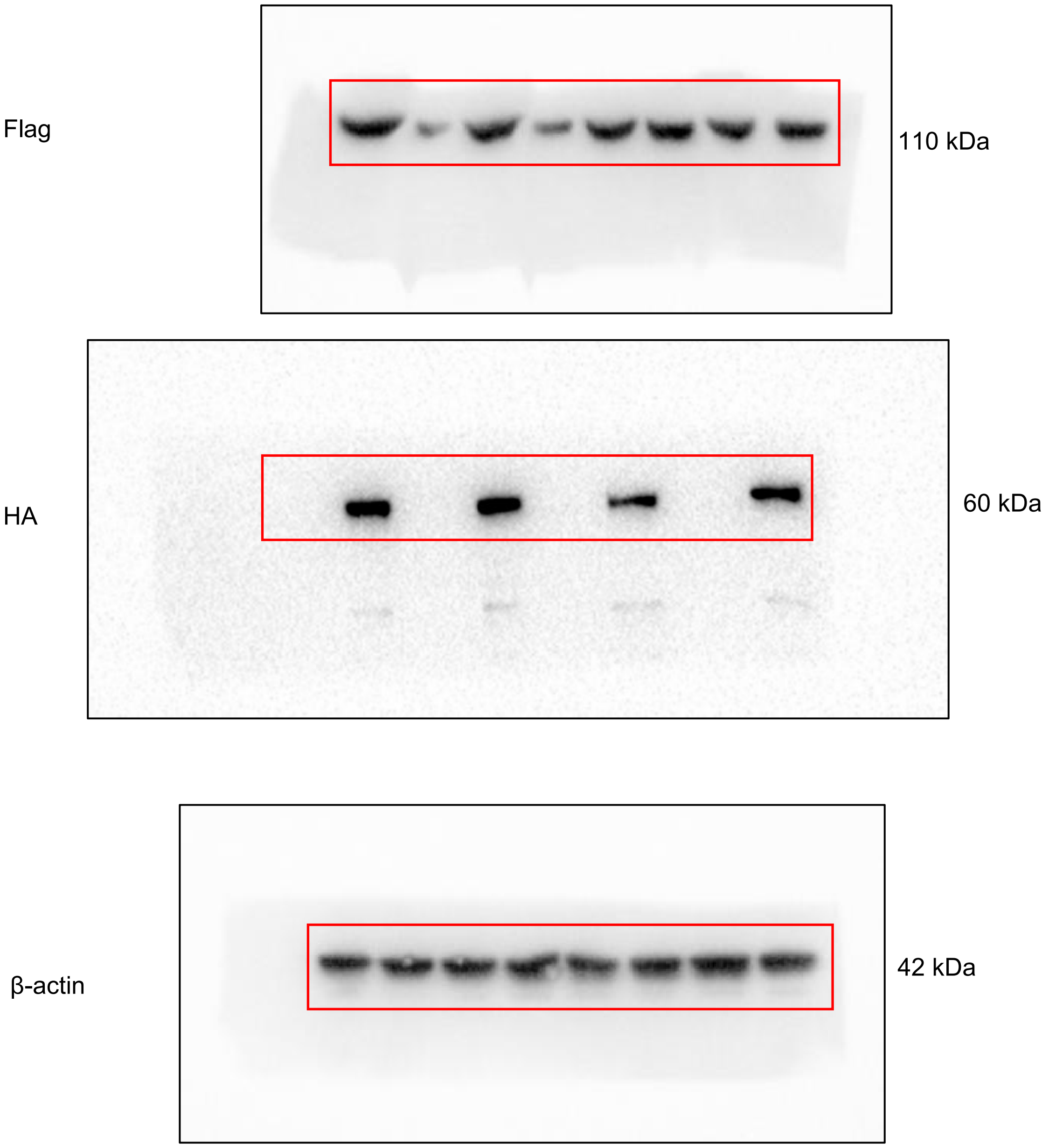

Figure 4F

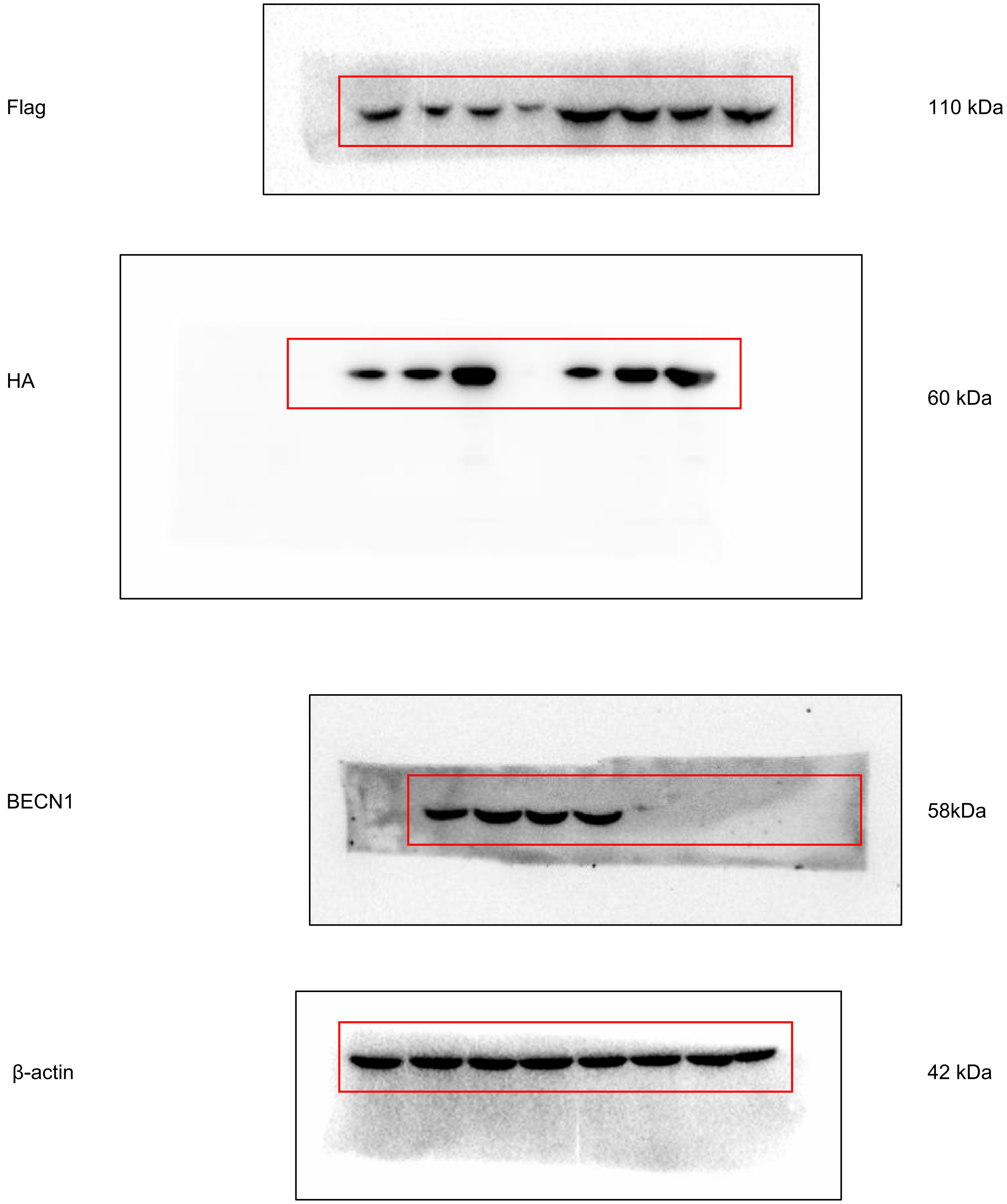

Figure 4G

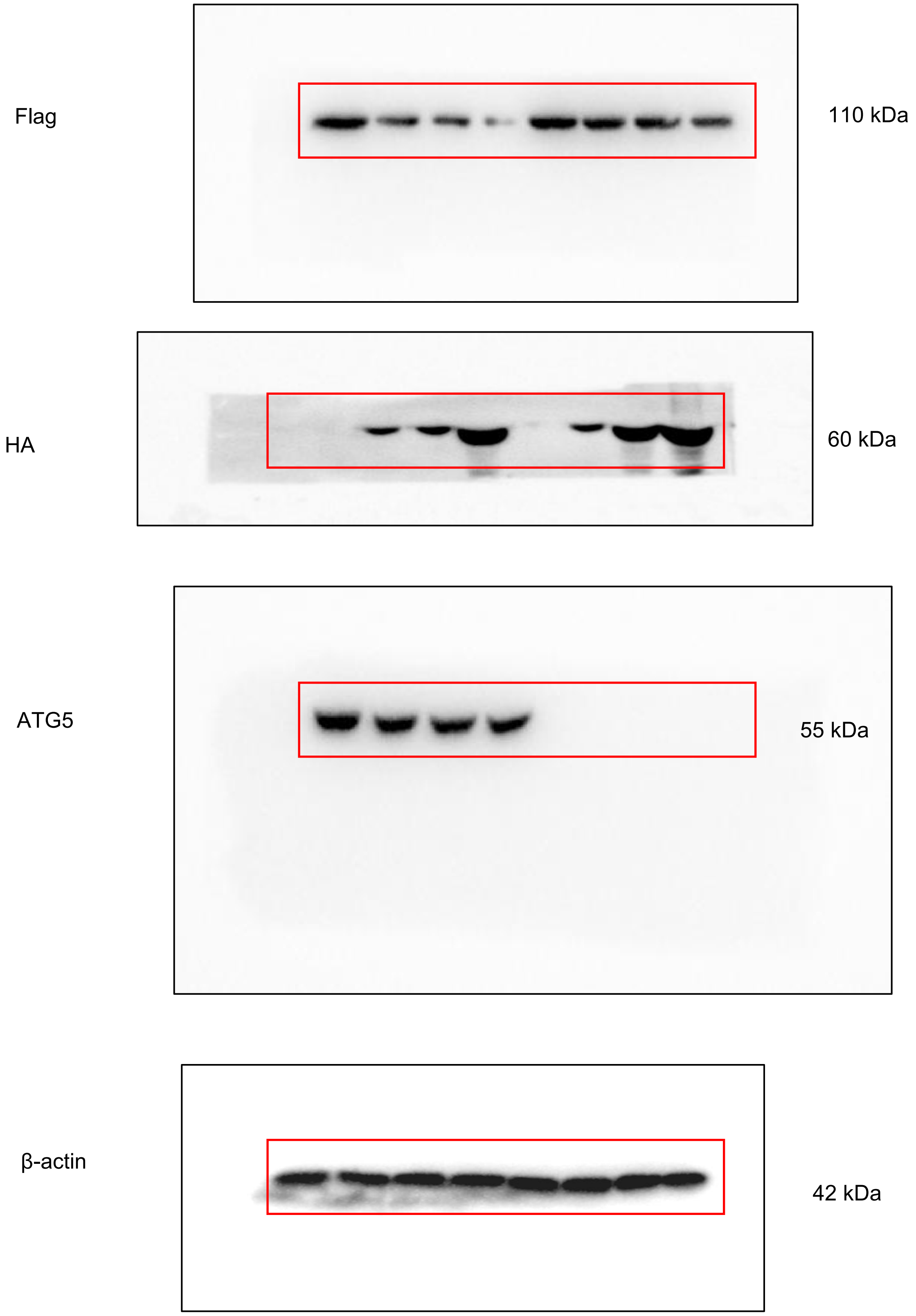

Figure 4H

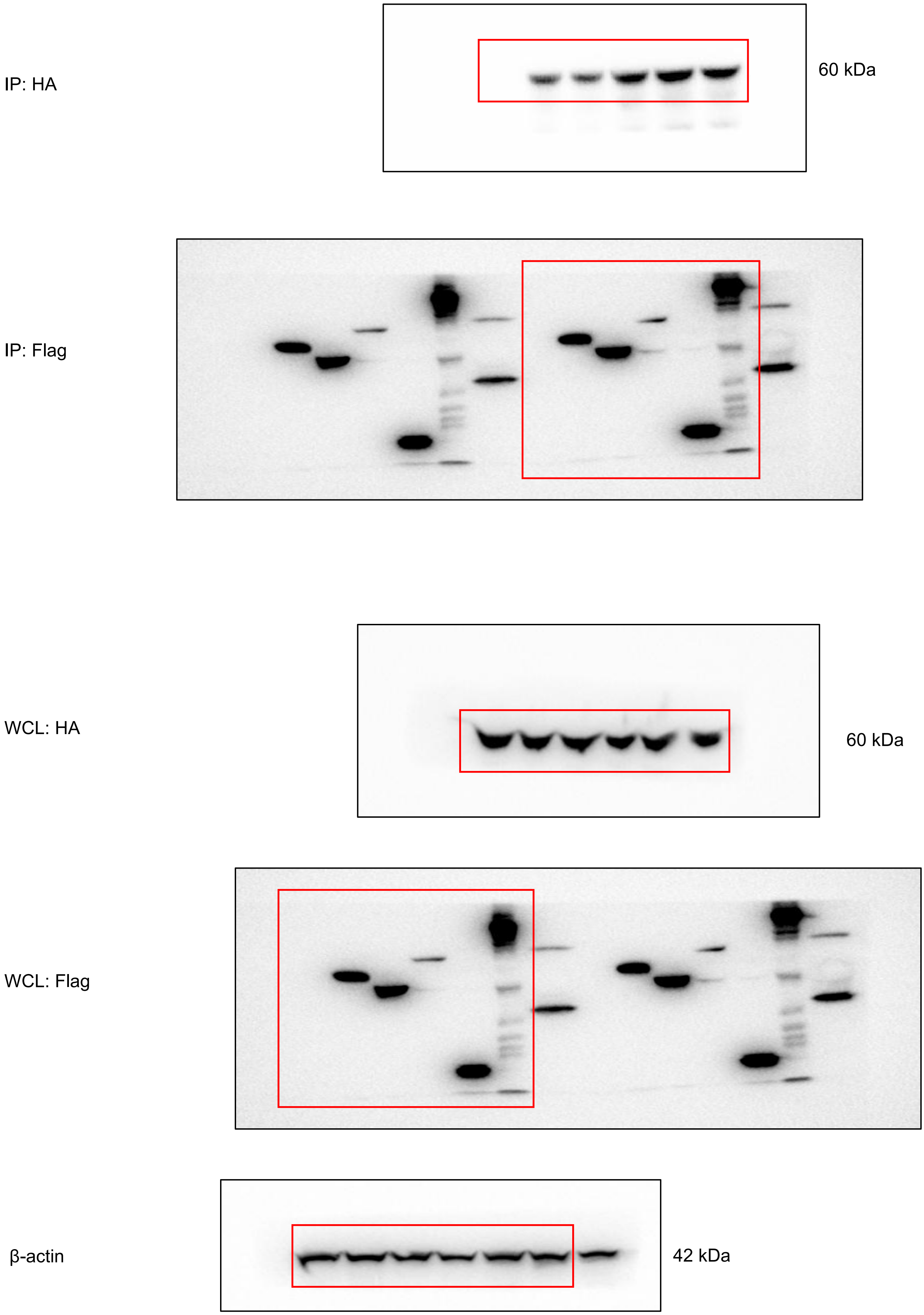

Figure 4I

IP: HA

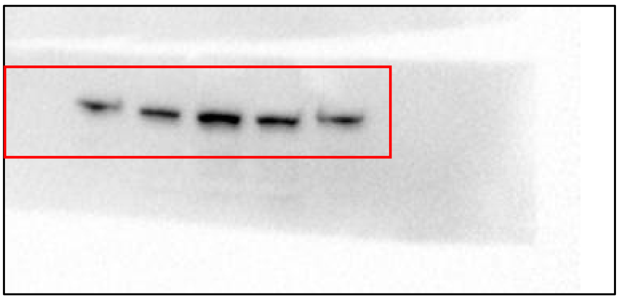

110 kDa

IP: Flag

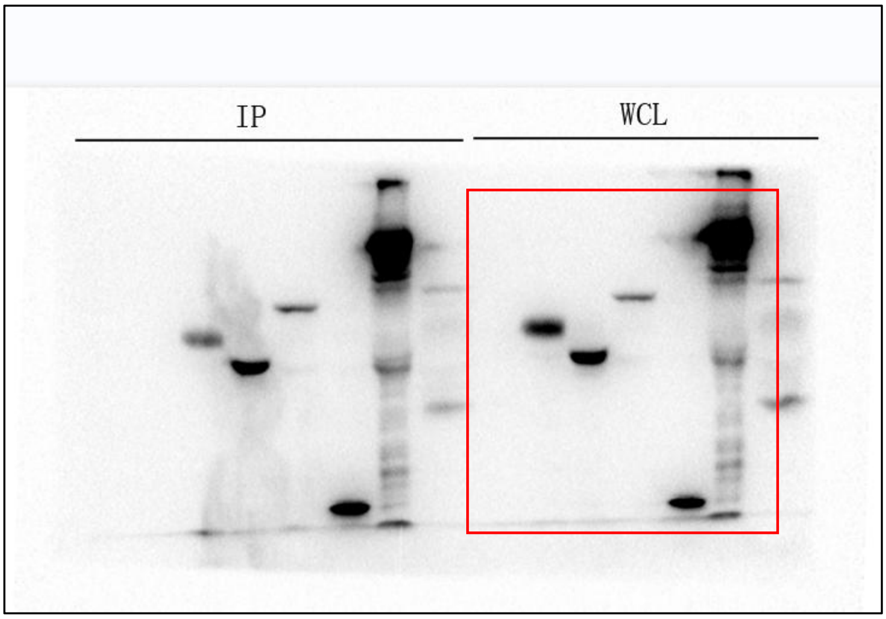

WCL: HA

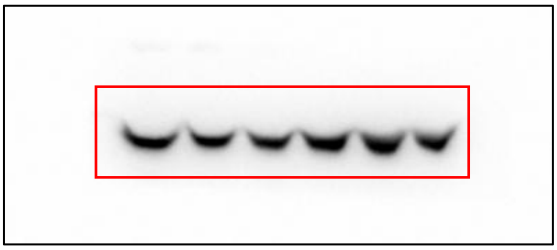

110 kDa

WCL: Flag

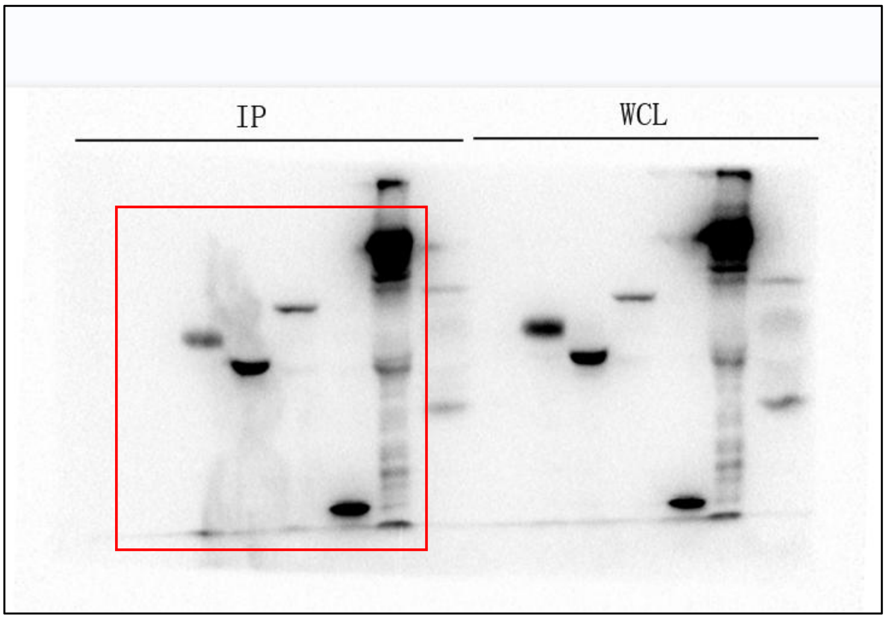

β-actin

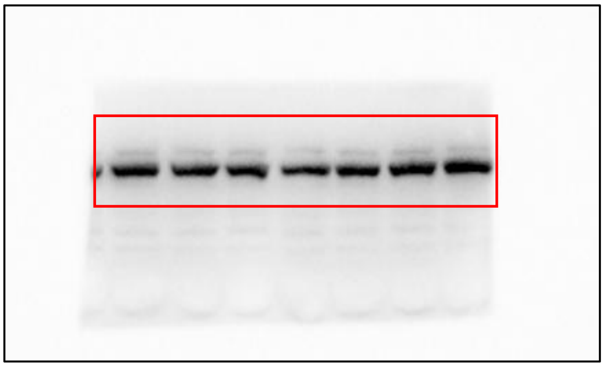

42 kDa

Figure 4J

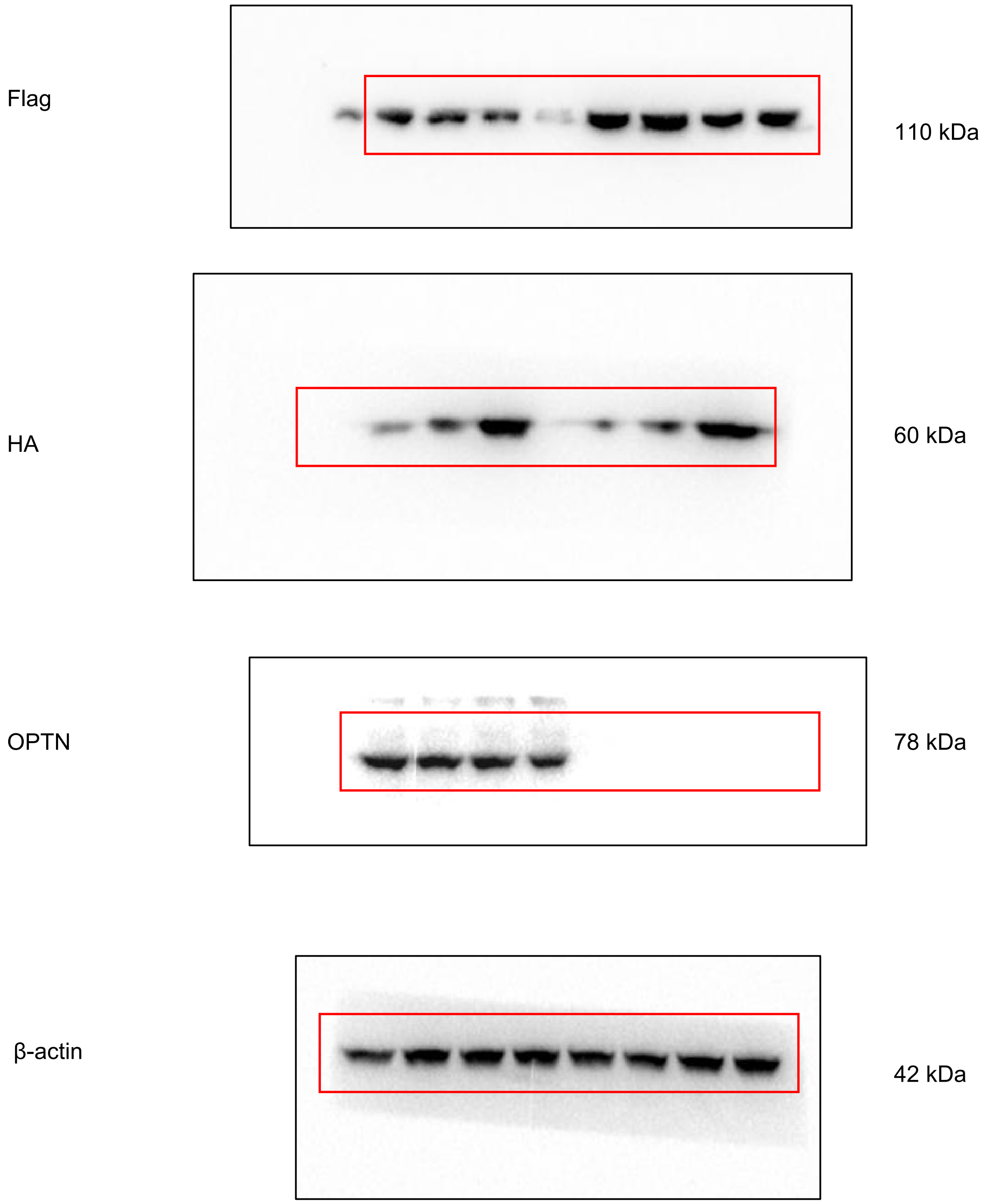

Figure 4L

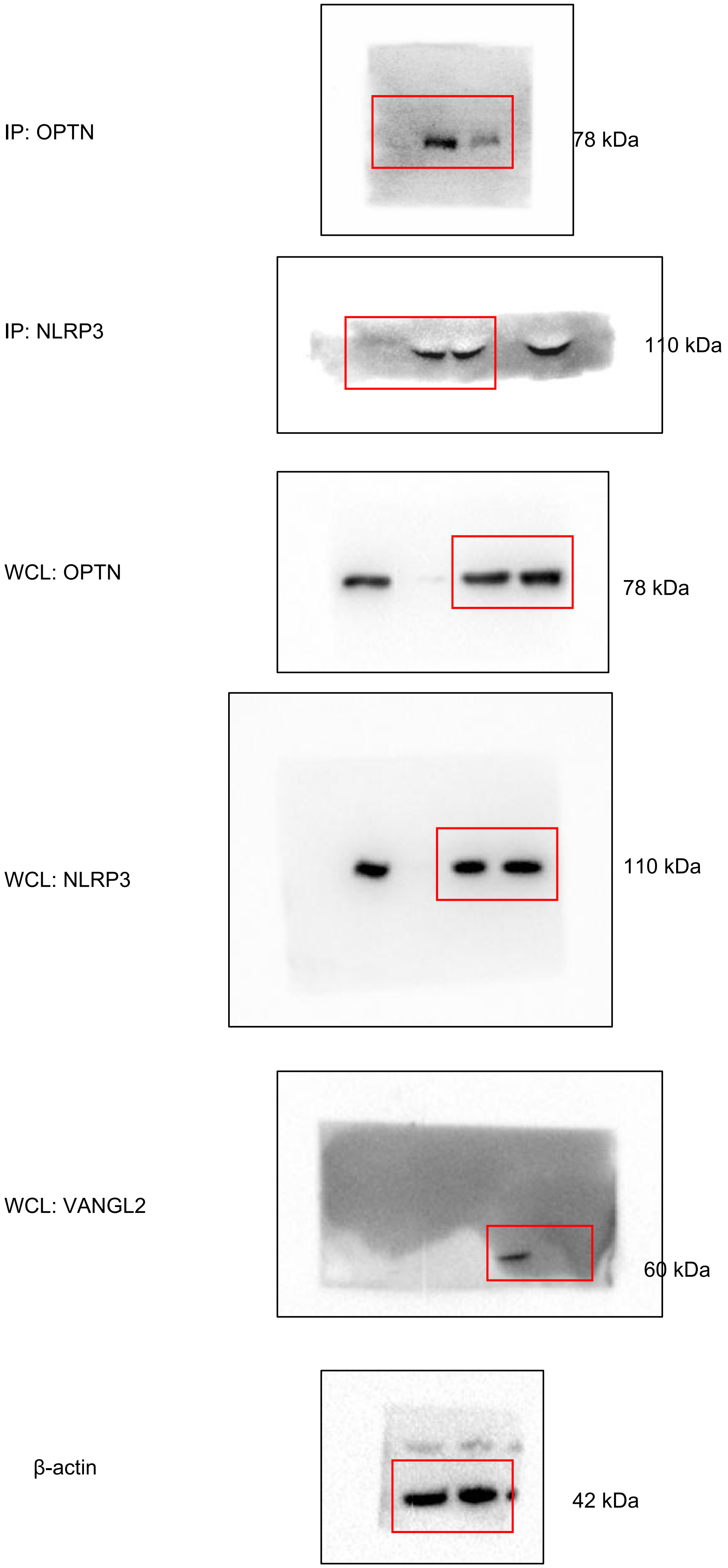
IP: OPTN
78 kDa
IP: NLRP3
110 kDa
WCL: OPTN
78 kDa
WCL: NLRP3
110 kDa
WCL: VANGL2
60 kDa
β-actin
42 kDa

Figure 4M

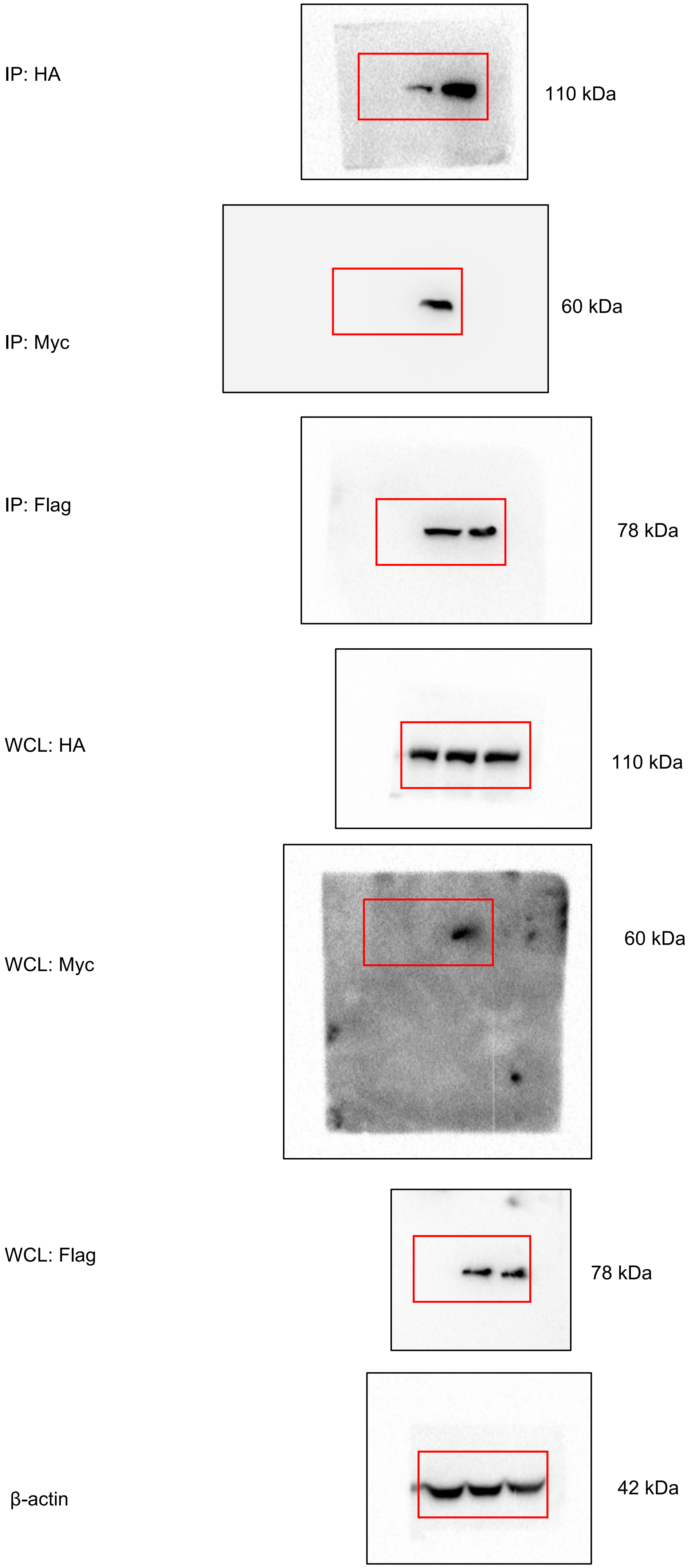
IP: HA
110 kDa
IP: Myc
60 kDa
IP: Flag
78 kDa
WCL: HA
110 kDa
WCL: Myc
60 kDa
WCL: Flag
78 kDa
β-actin
42 kDa

Figure 4N

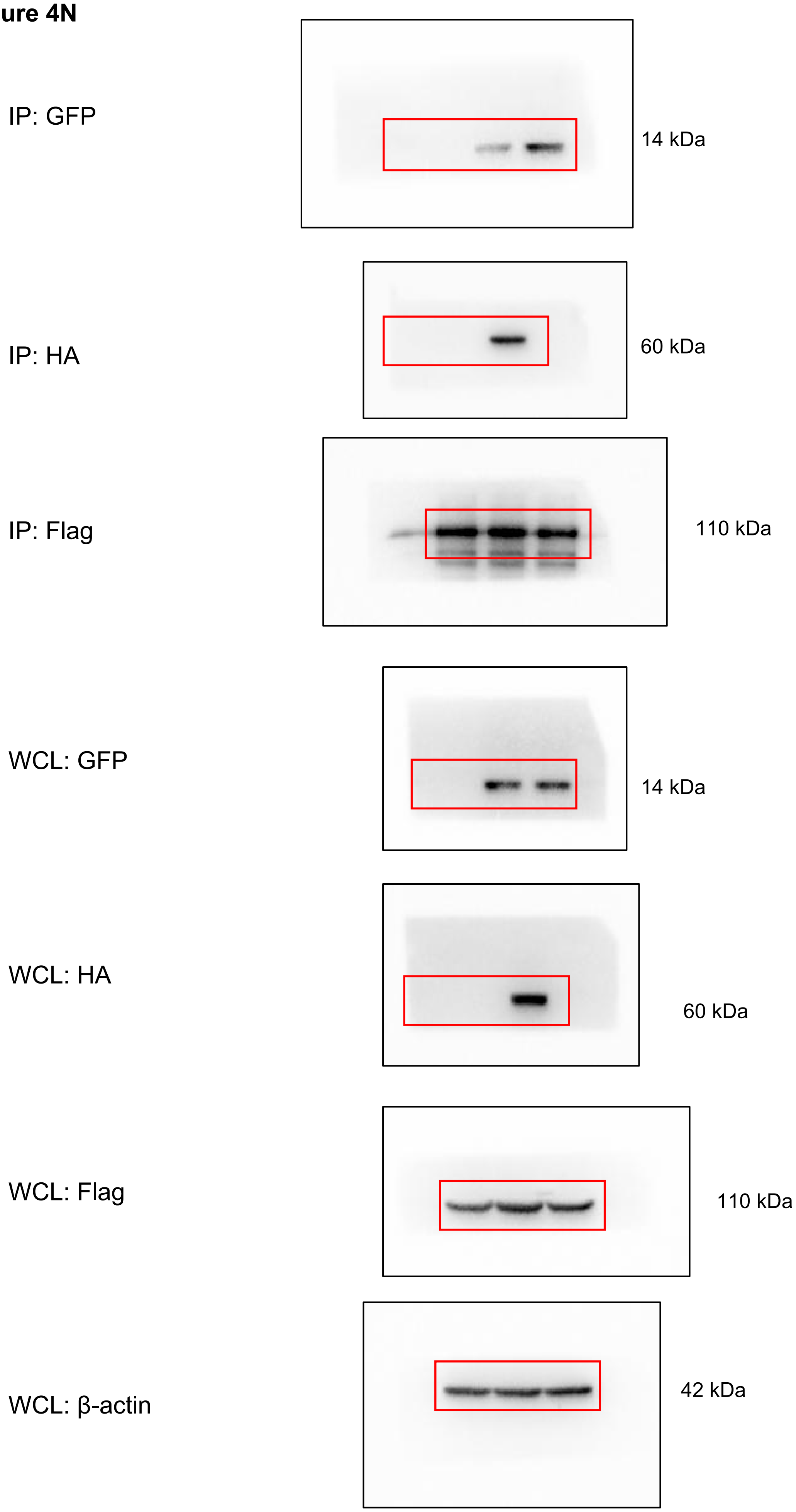
IP: GFP
14 kDa
IP: HA
60 kDa
IP: Flag
110 kDa
WCL: GFP
14 kDa
WCL: HA
60 kDa
WCL: Flag
110 kDa
WCL: β-actin
42 kDa

Figure S4A

Flag

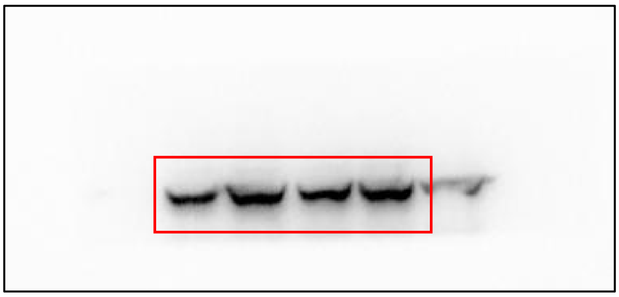

22 kDa

HA

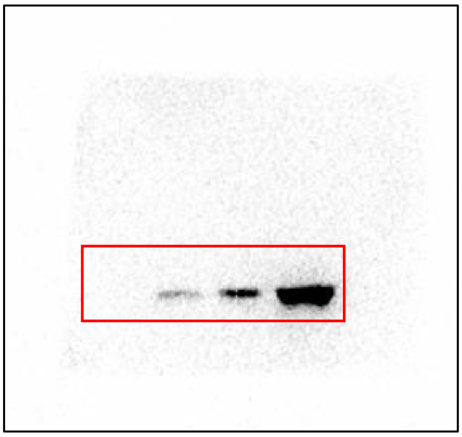

60 kDa

β-actin

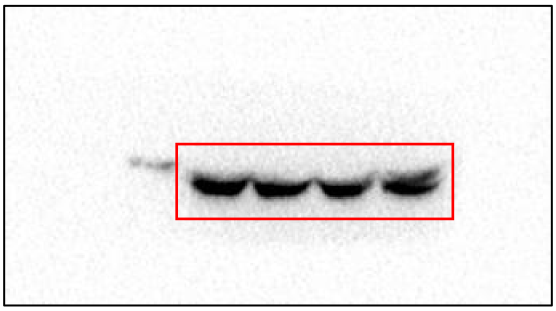

42 kDa

Figure S4B

Flag

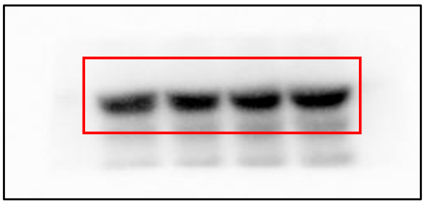

60 kDa

HA

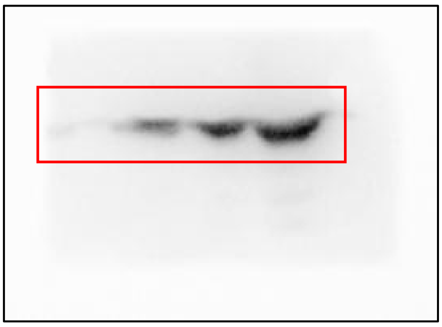

48 kDa

β-actin

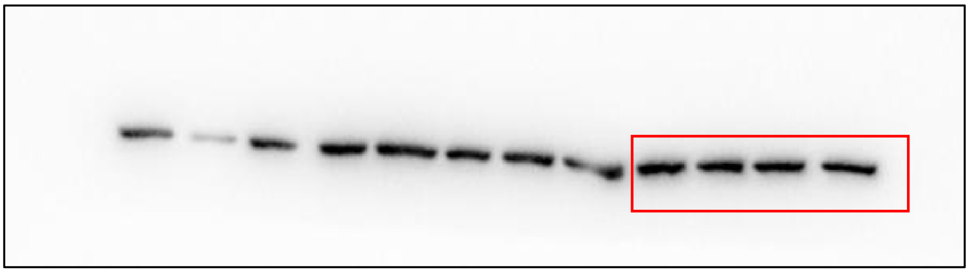

42 kDa

Figure S4C

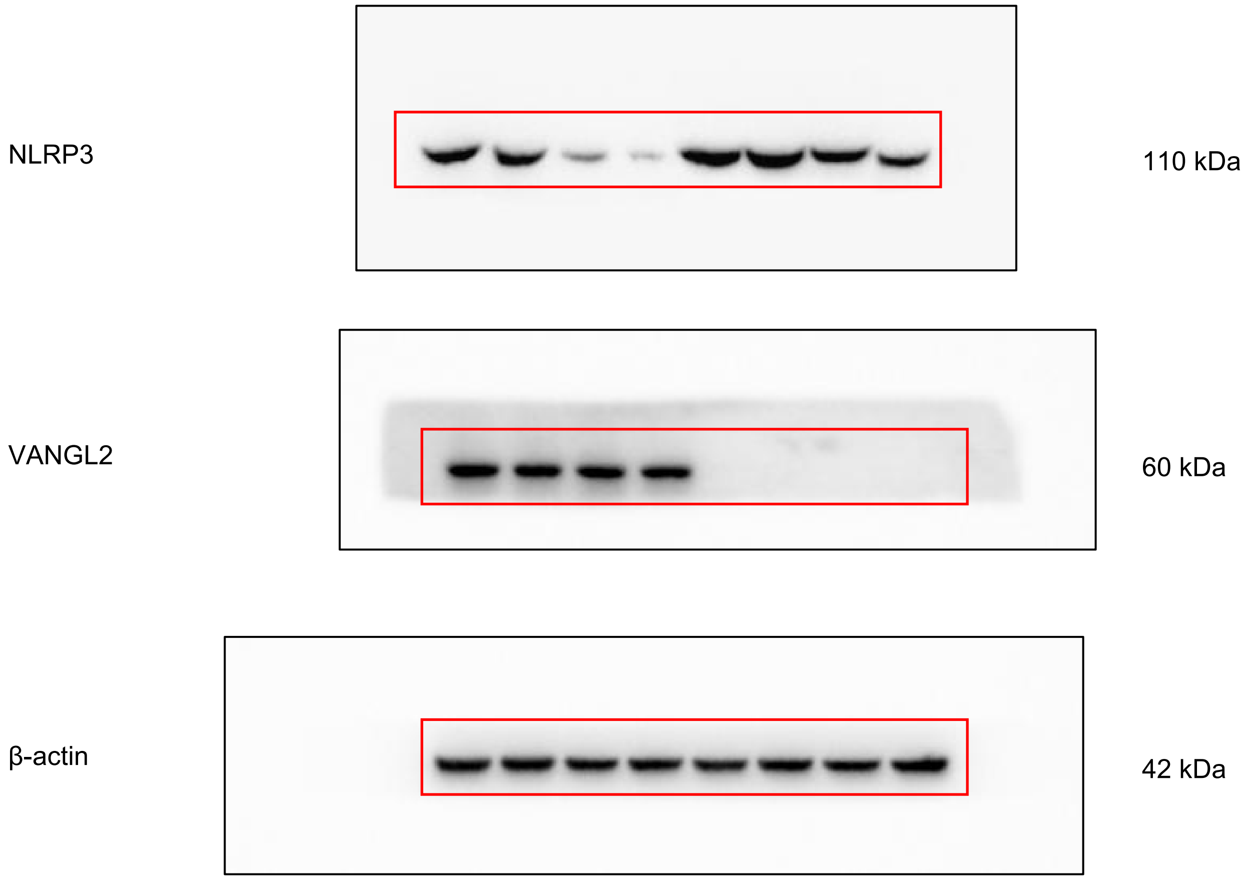

Figure S4D

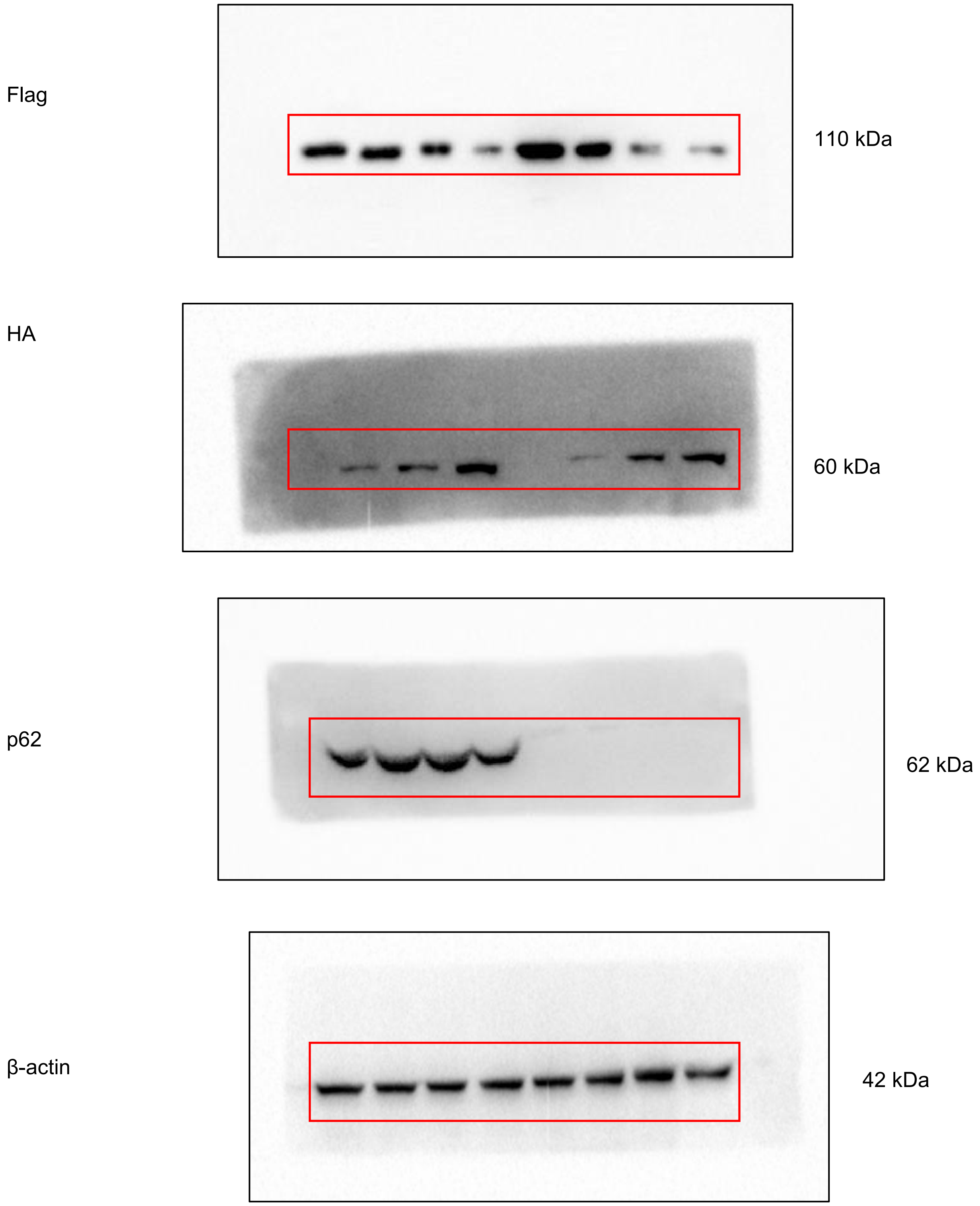

Figure S4E

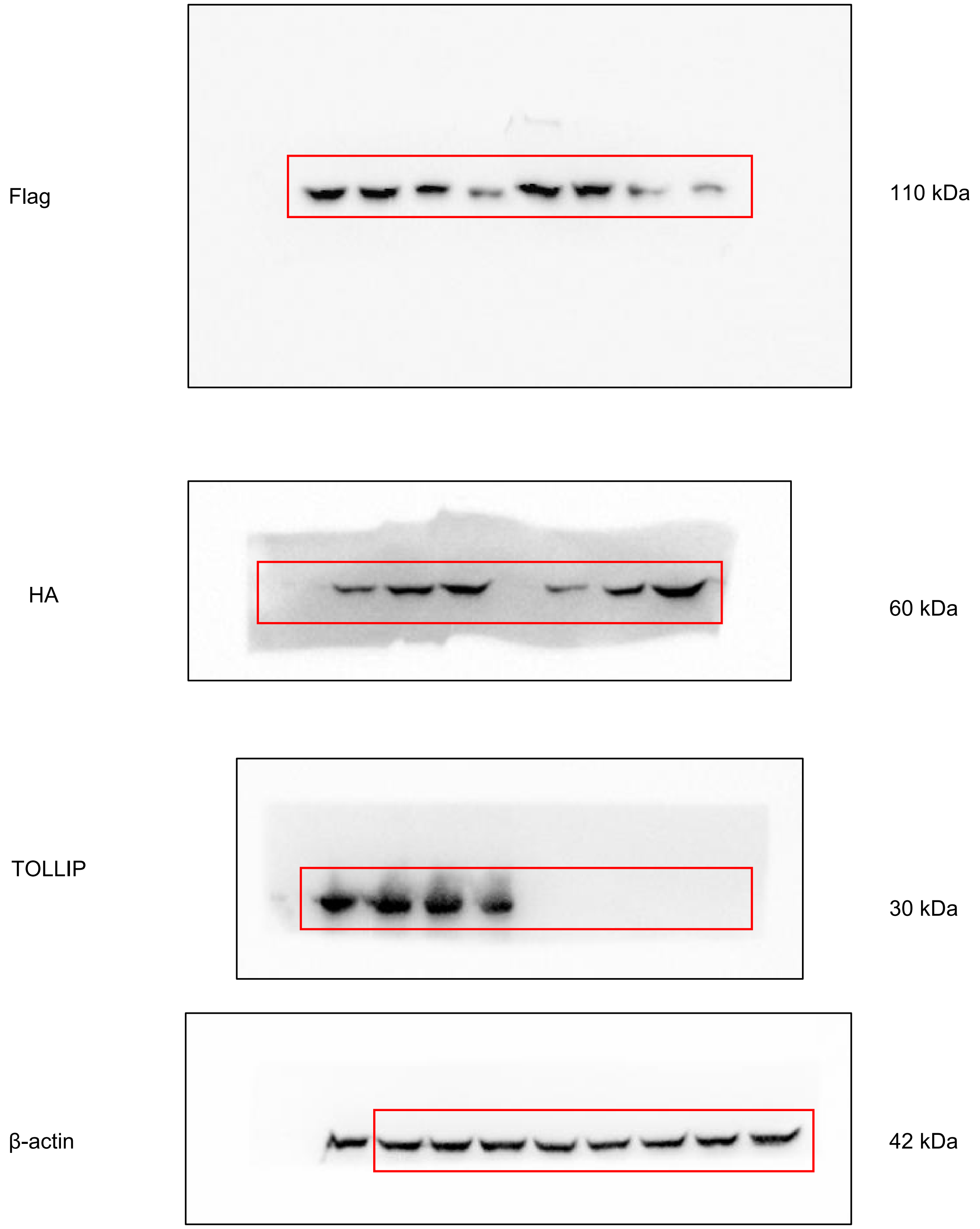

Figure S4F

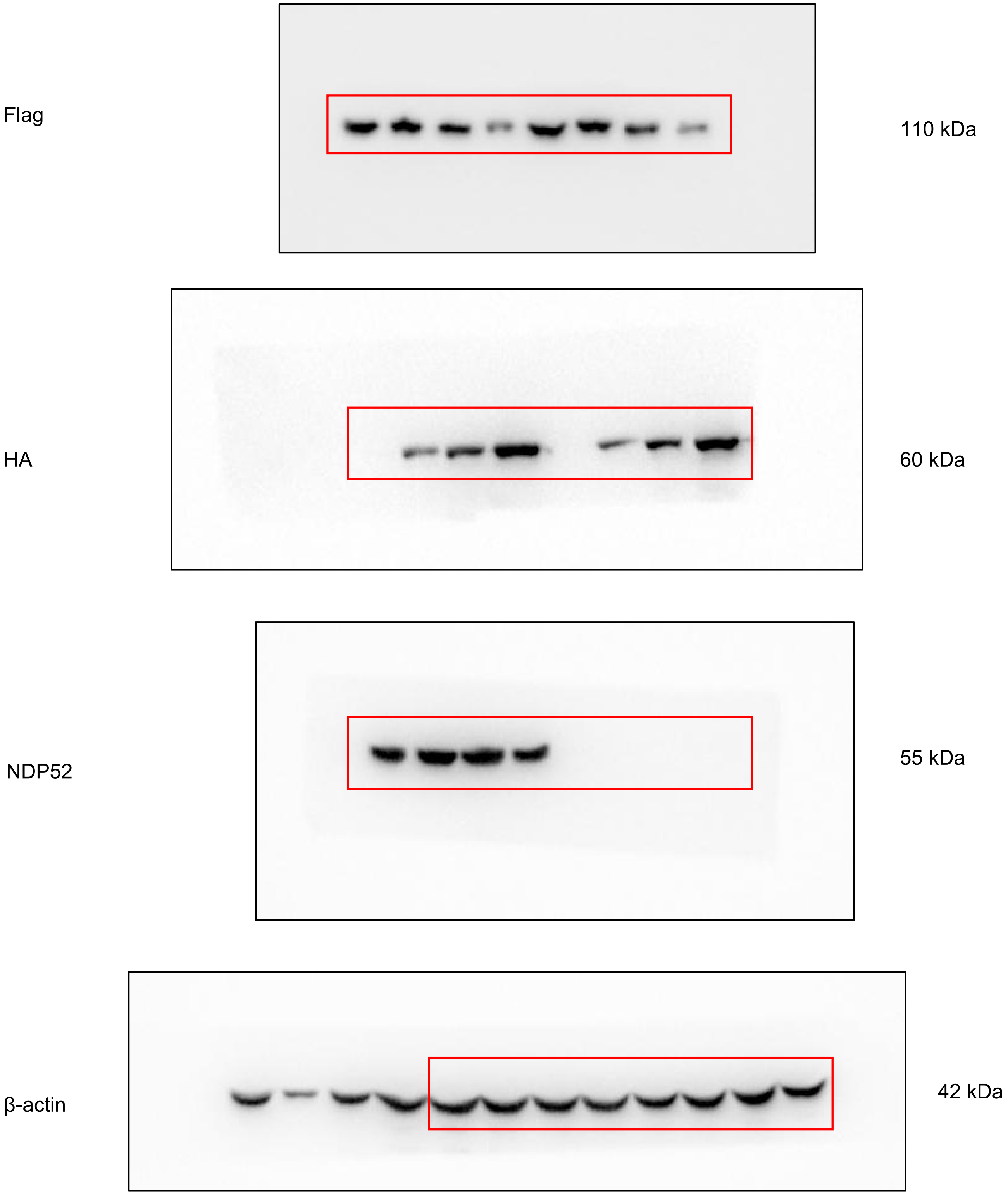

Figure S4G

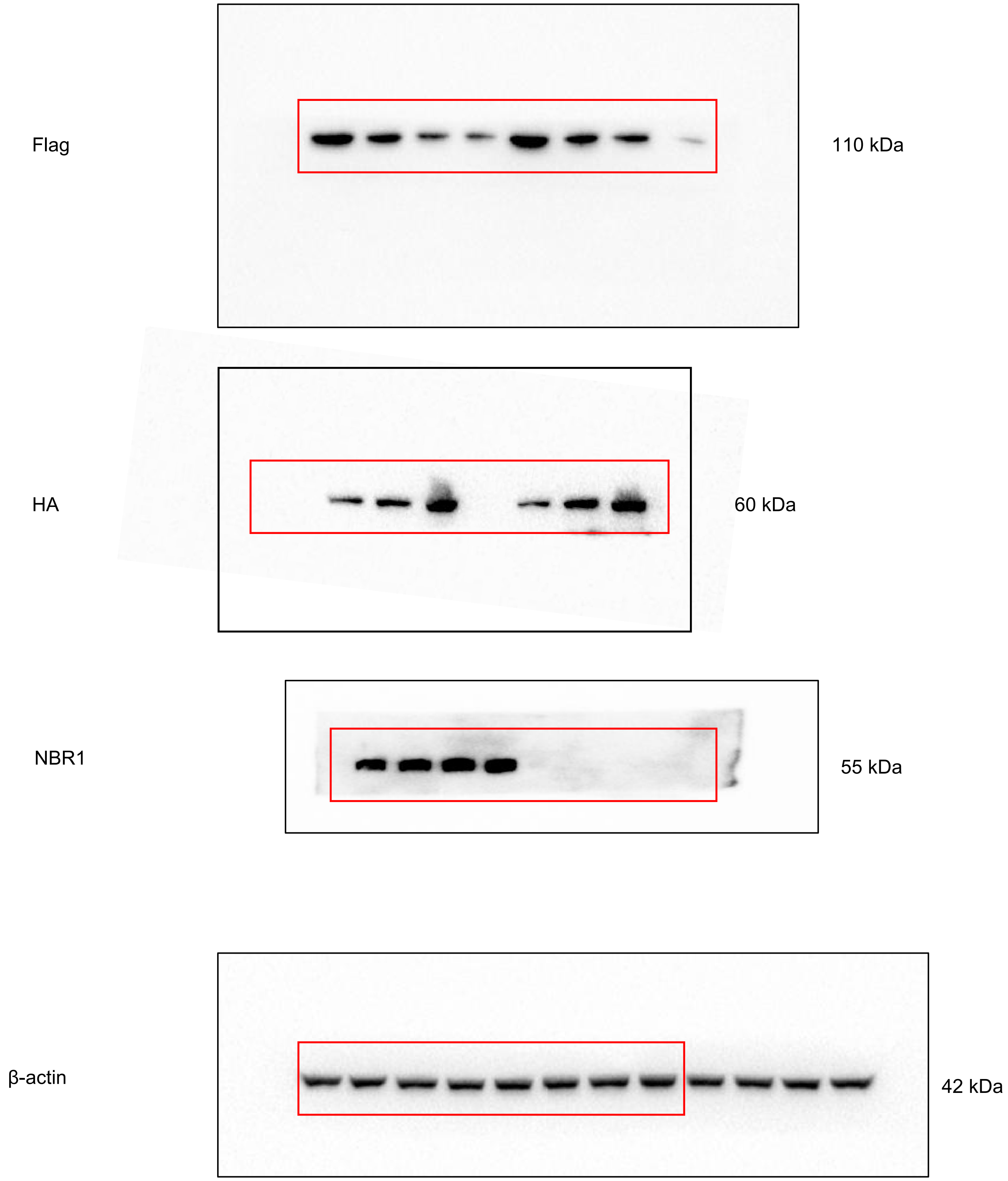

Figure S4H

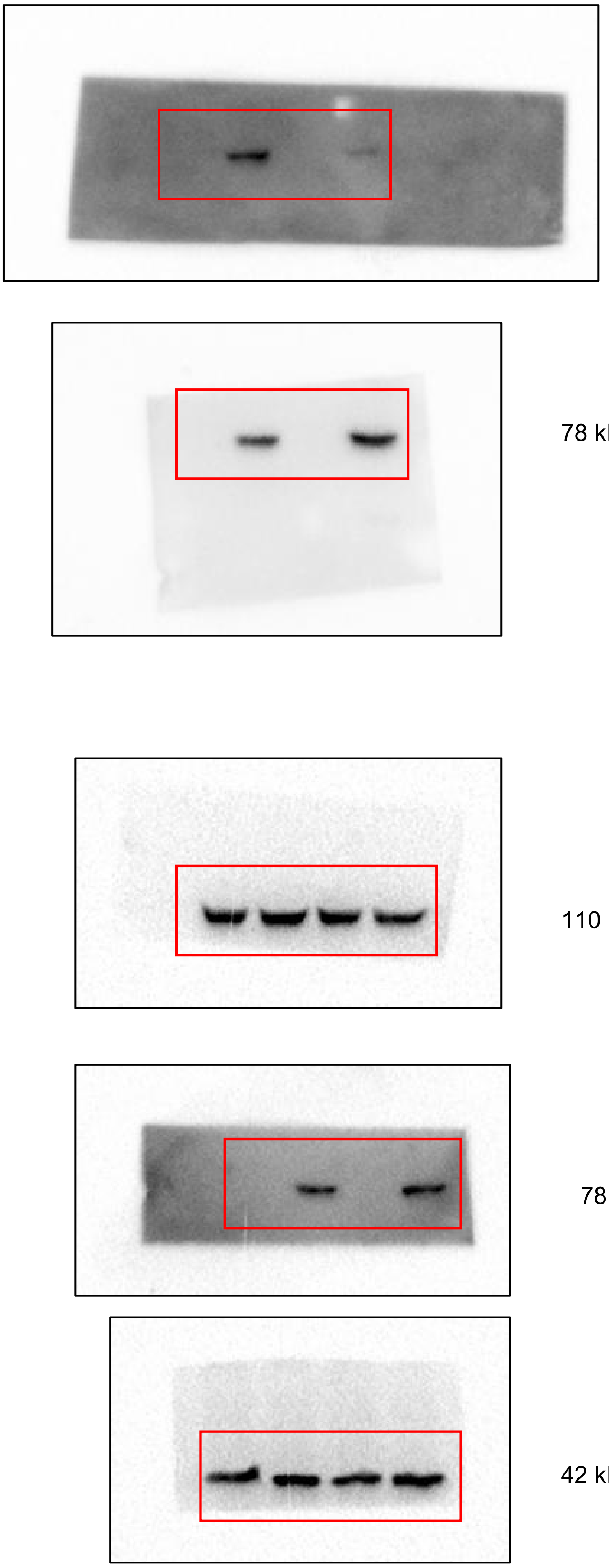

IP: HA — 110 kDa

IP: Flag — 78 kDa

WCL: HA — 110 kDa

WCL: Flag — 78 kDa

β-actin — 42 kDa

Figure S4I

IP: IP: LC3B

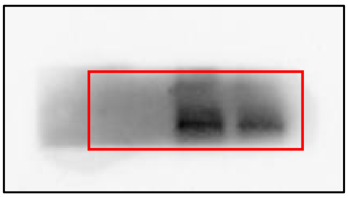

14 kDa

IP: NLRP3

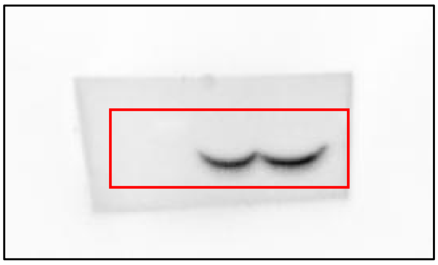

110 kDa

WCL: LC3B

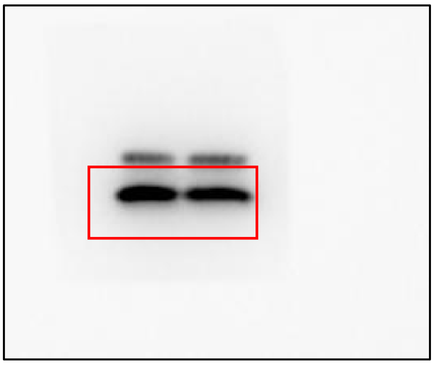

14 kDa

WCL: NLRP3

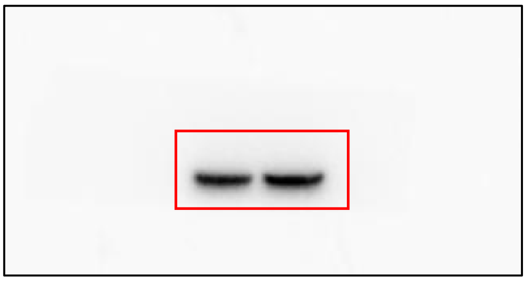

110 kDa

WCL: VANGL2

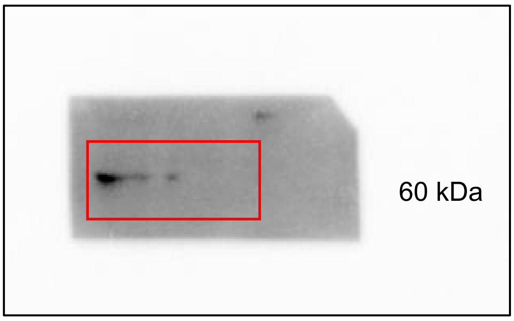

β-actin

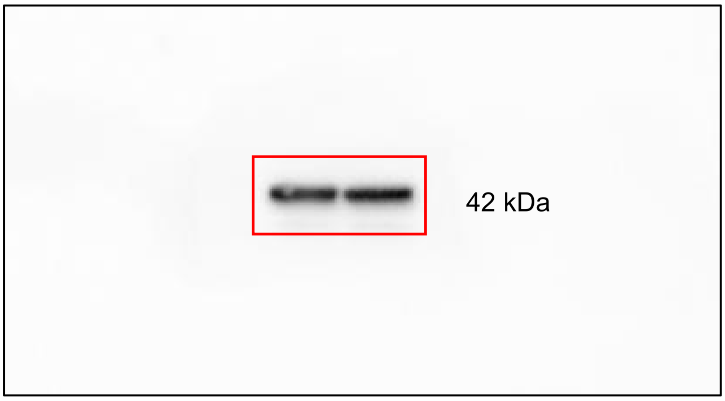

Figure S4J

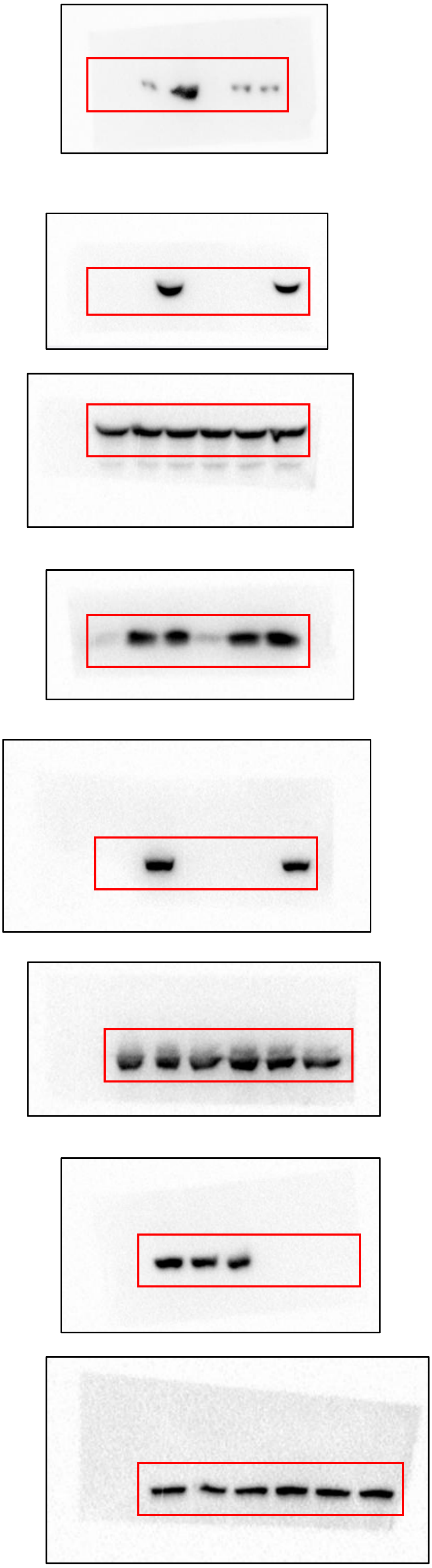

IP: GFP — 14 kDa

IP: HA — 60 kDa

IP: Flag — 110 kDa

WCL: GFP — 14 kDa

WCL: HA — 60 kDa

WCL: Flag — 110 kDa

OPTN — 78 kDa

β-actin — 42 kDa

Figure 5A

IP: Ub

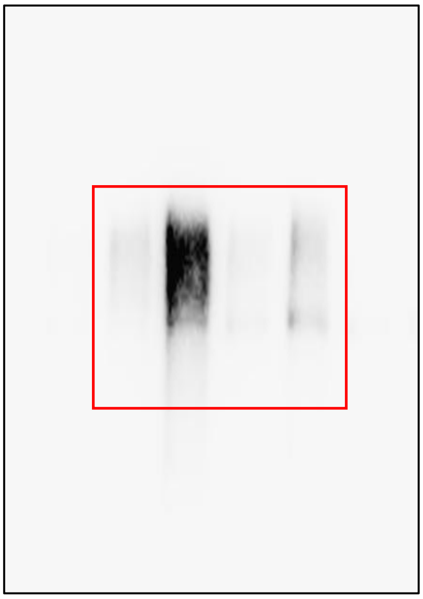

IP: NLRP3

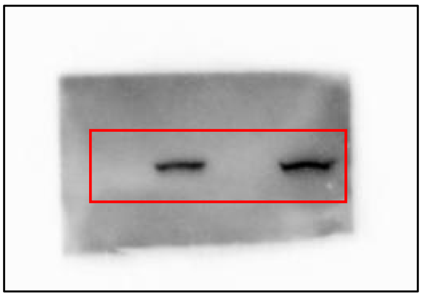

110 kDa

WCL: NLRP3

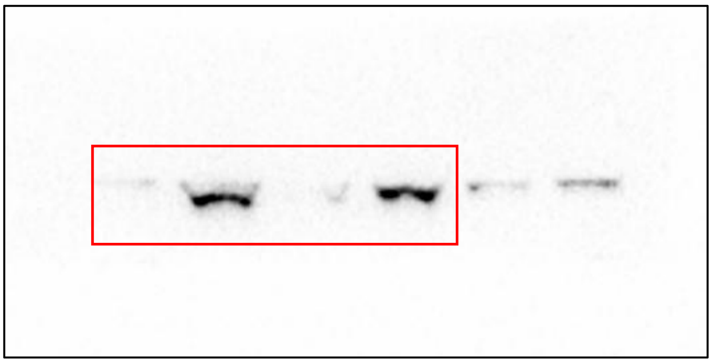

110 kDa

WCL: β-actin

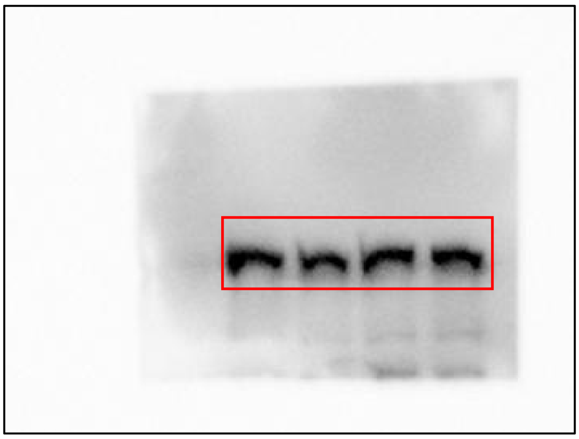

42 kDa

Figure 5B

Flag

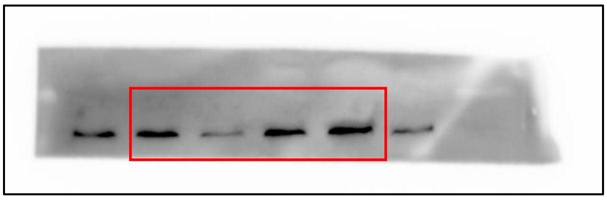

110 kDa

HA

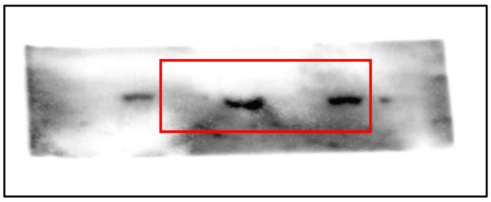

60 kDa

β-actin

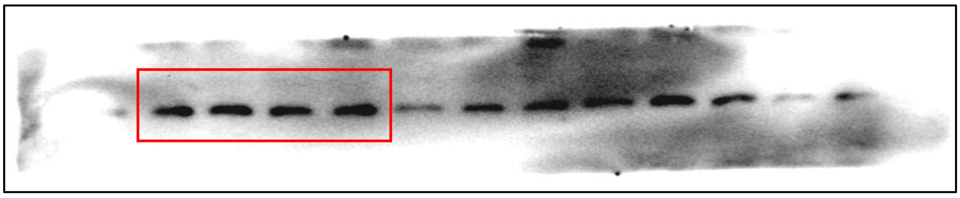

42 kDa

Figure 5C

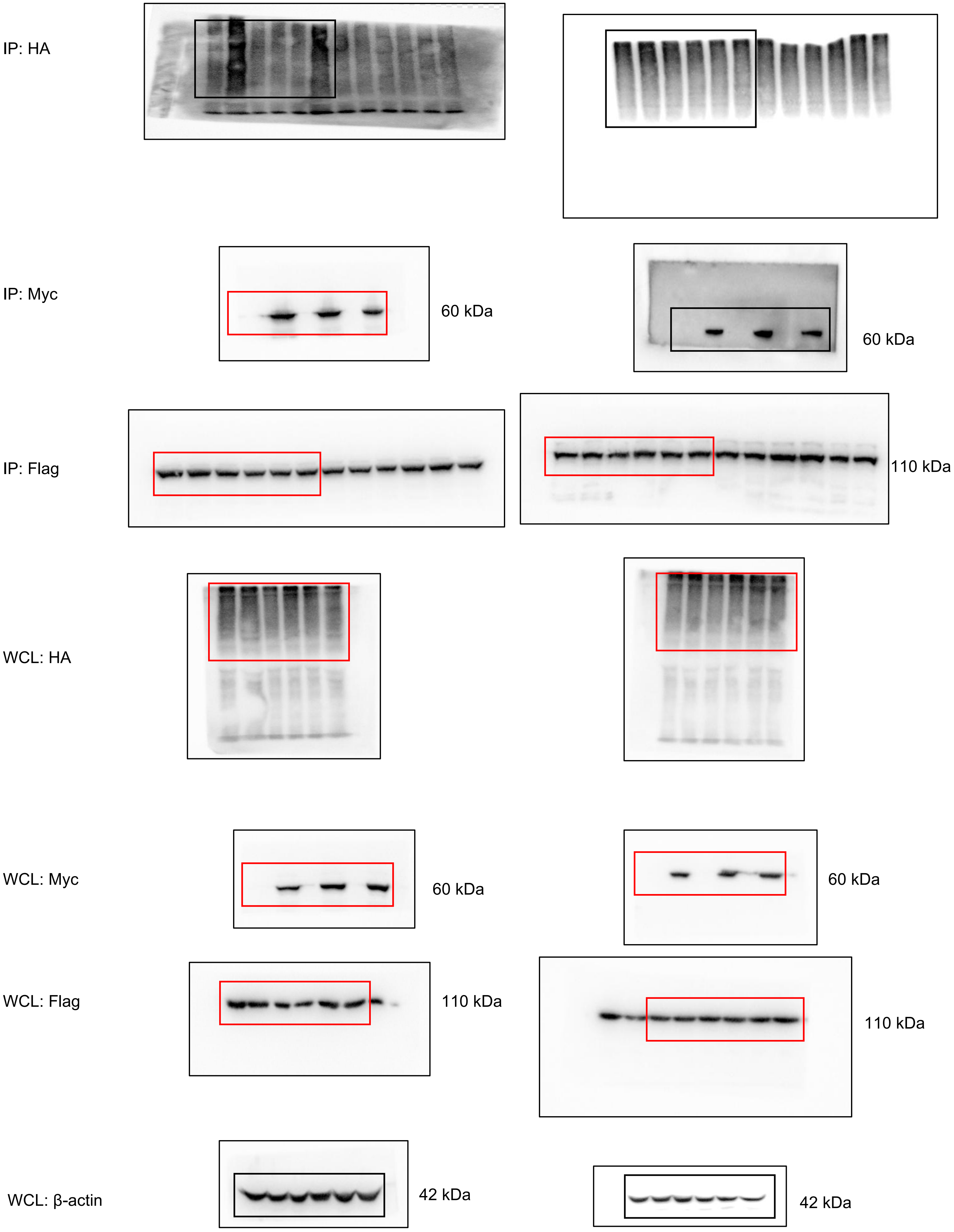
IP: HA
IP: Myc
60 kDa
60 kDa
IP: Flag
110 kDa
WCL: HA
WCL: Myc
60 kDa
60 kDa
WCL: Flag
110 kDa
110 kDa
WCL: β-actin
42 kDa
42 kDa

Figure 5D

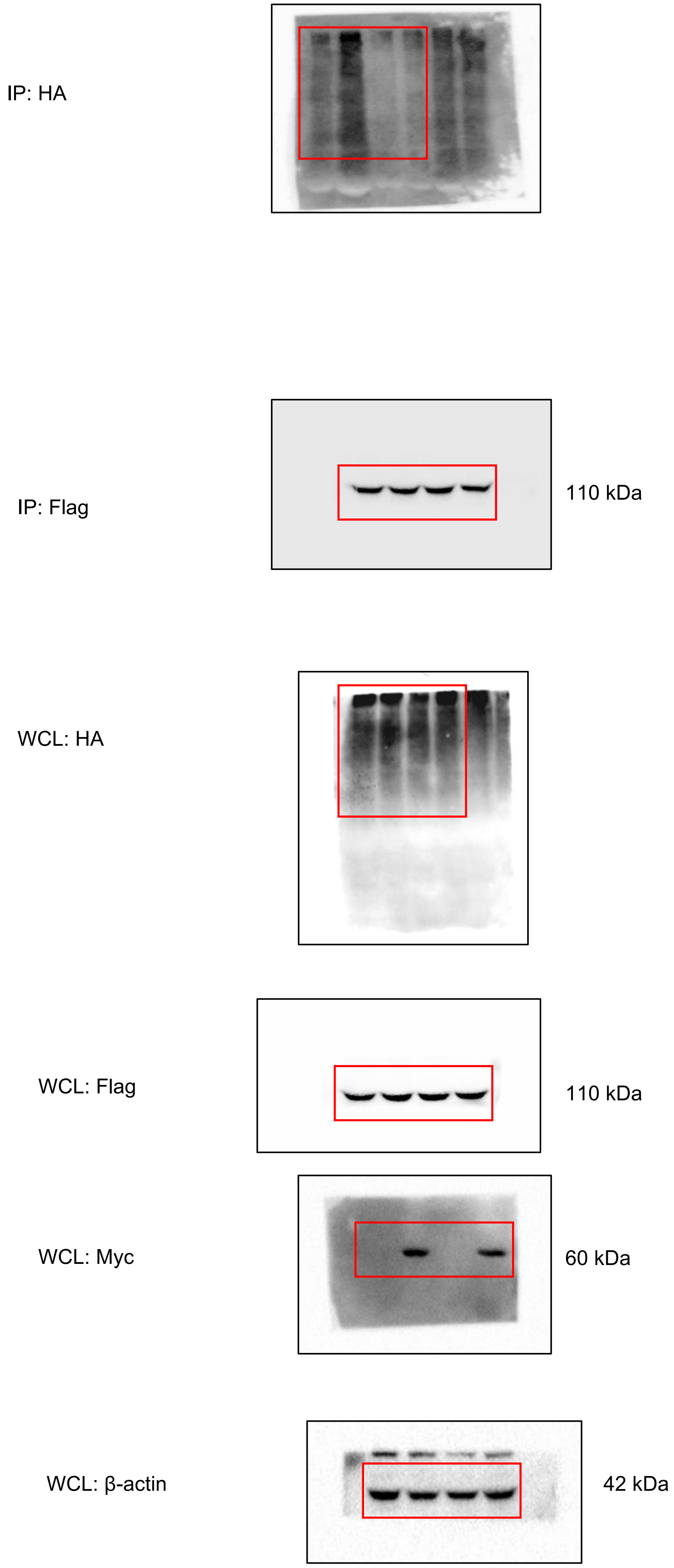
IP: HA
IP: Flag
110 kDa
WCL: HA
WCL: Flag
110 kDa
WCL: Myc
60 kDa
WCL: β-actin
42 kDa

Figure 5E

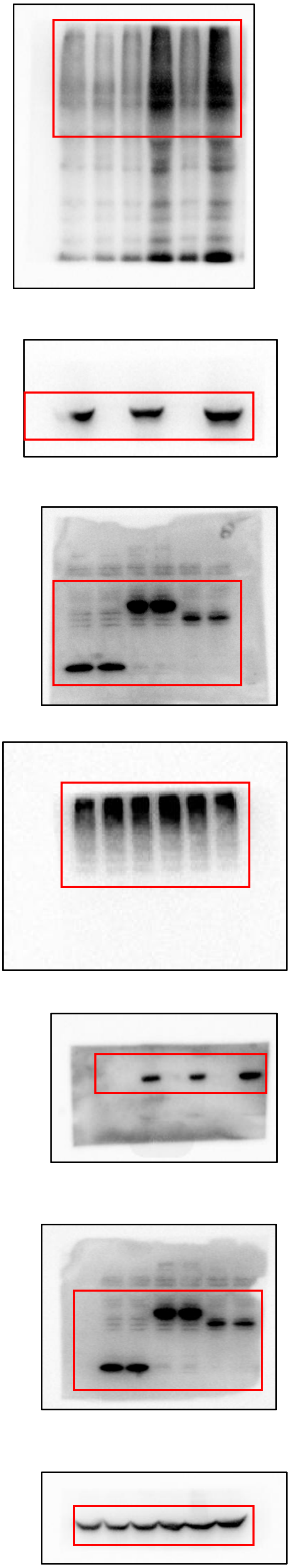
IP: HA
IP: Myc
60 kDa
IP: Flag
WCL: HA
WCL: Myc
60 kDa
WCL: Flag
WCL: β-actin
42 kDa

Figure 5F

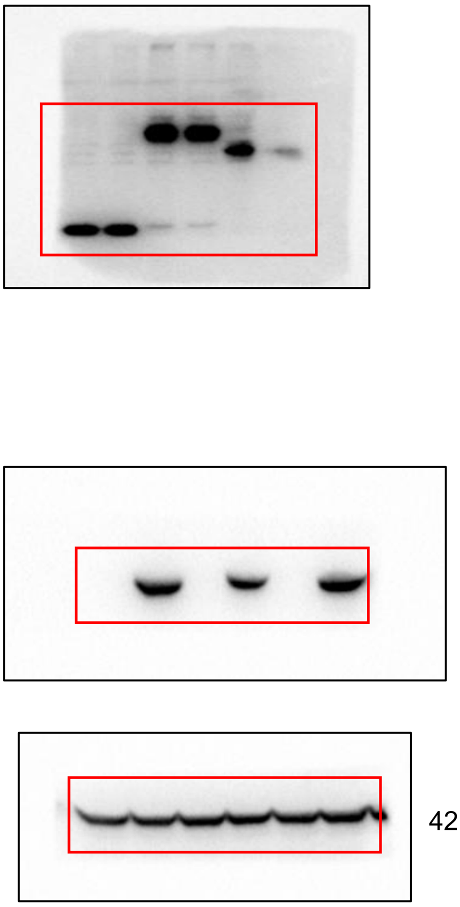

Flag

HA

60 kDa

β-actin

42 kDa

Figure 5G

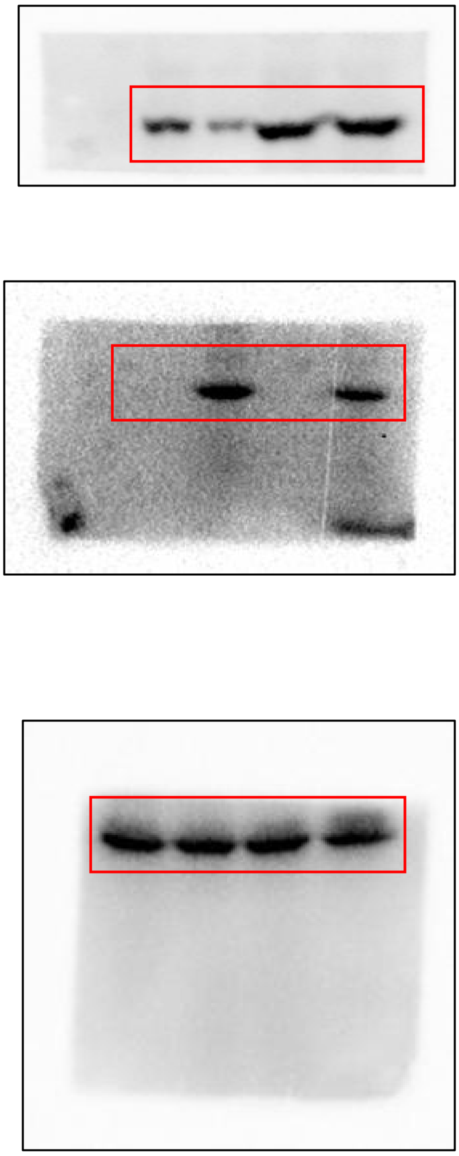

Flag 110 kDa

Myc 60 kDa

β-actin 42 kDa

Figure 5H

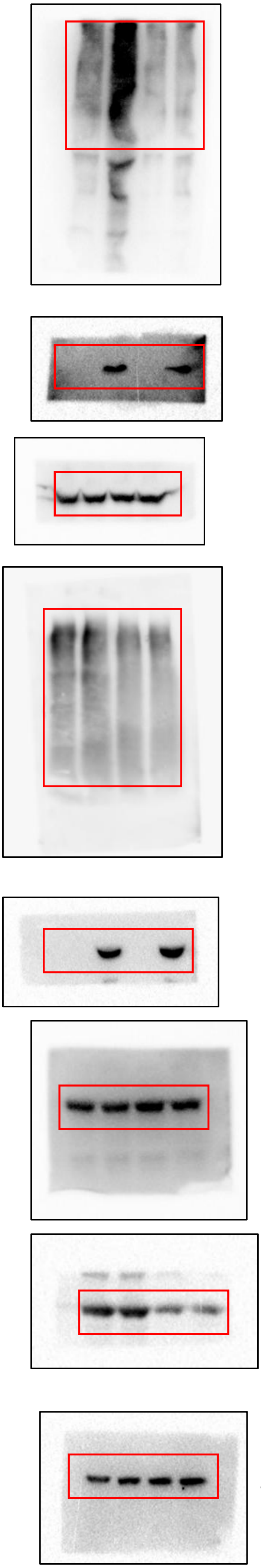

IP: HA

IP: Myc 60 kDa

IP: Flag 110 kDa

WCL: HA

WCL: Myc 60 kDa

WCL: Flag 110 kDa

WCL: MARCH8 33 kDa

WCL: β-actin 42 kDa

Figure 5J

IP: MARCH8

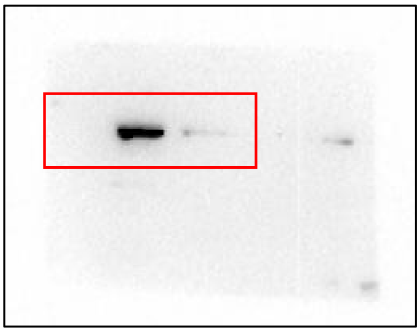

33 kDa

IP: NLPR3

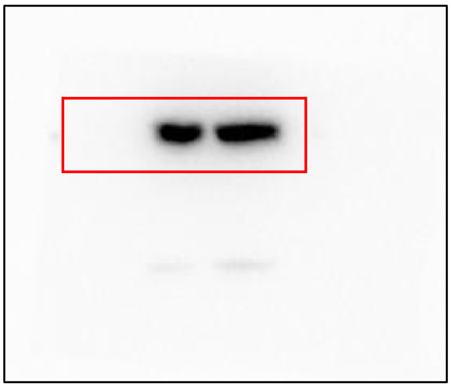

110 kDa

WCL: MARCH8

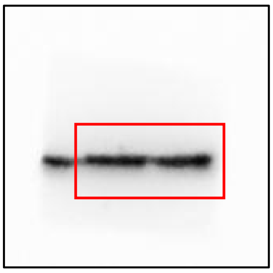

33 kDa

WCL: NLPR3

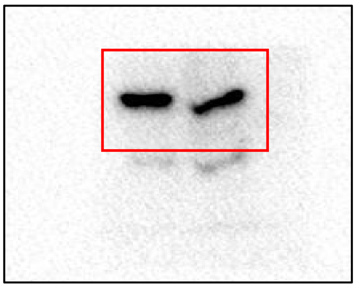

110 kDa

WCL: β-actin

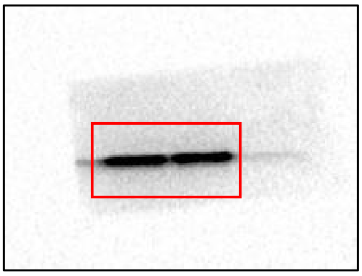

42 kDa

Figure 5K

IP: GFP

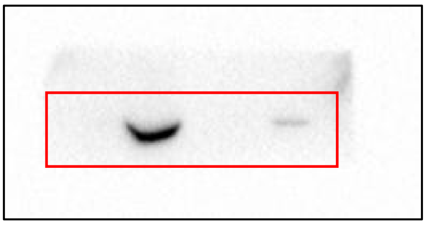

33 kDa

IP: Flag

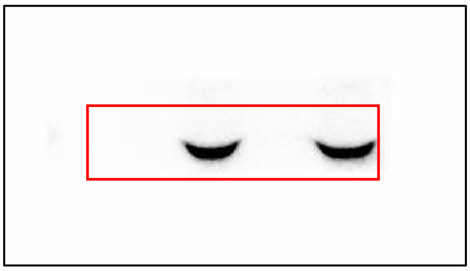

110 kDa

WCL: GFP

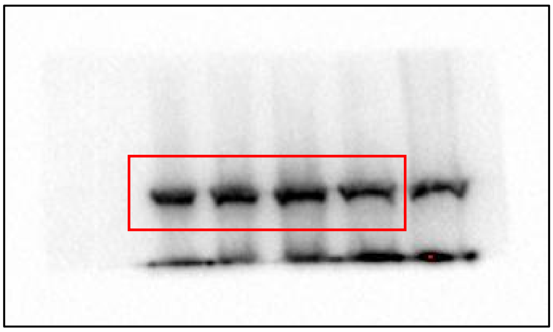

33 kDa

WCL: Flag

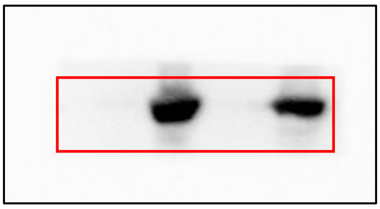

110 kDa

WCL: β-actin

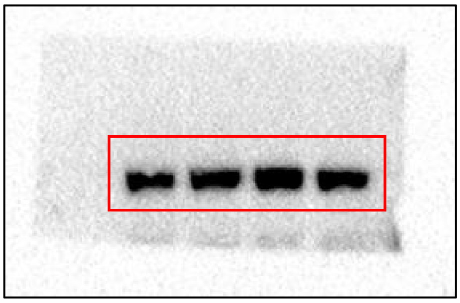

42 kDa

Figure 5L

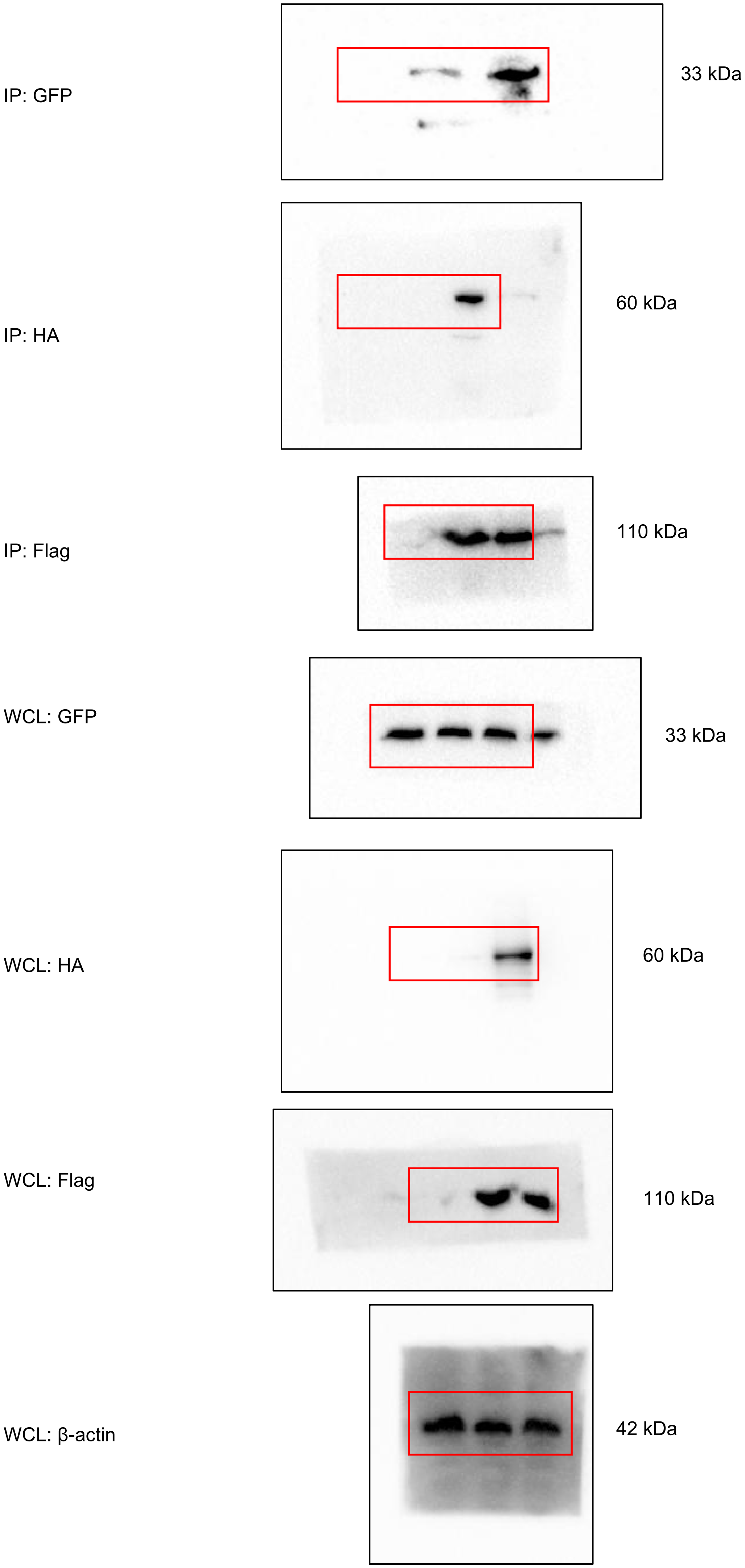
IP: GFP
33 kDa
IP: HA
60 kDa
IP: Flag
110 kDa
WCL: GFP
33 kDa
WCL: HA
60 kDa
WCL: Flag
110 kDa
WCL: β-actin
42 kDa

Figure 5M

IP: GFP

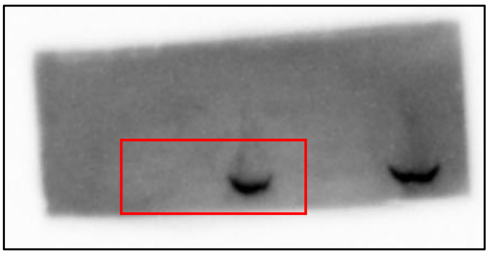

33 kDa

IP: Flag

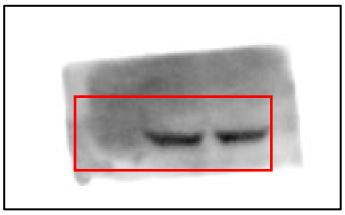

60 kDa

WCL: GFP

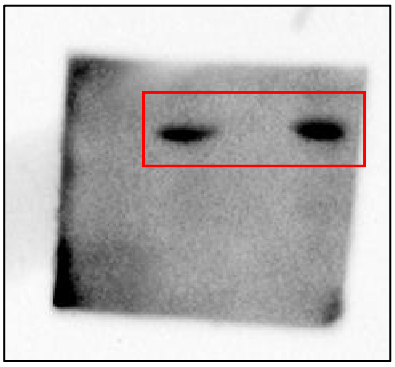

33 kDa

WCL: Flag

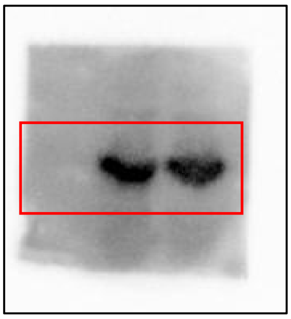

60 kDa

WCL: β-actin

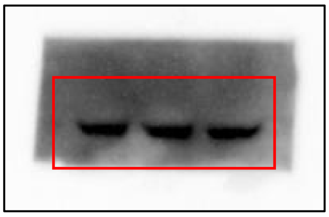

42 kDa

Figure 5N

IP: MARCH8

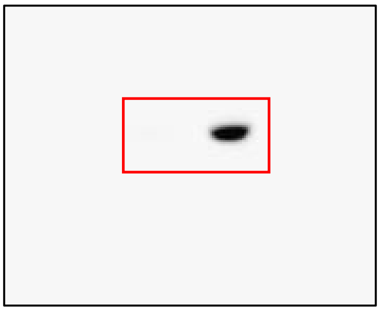

33 kDa

IP: VANGL2

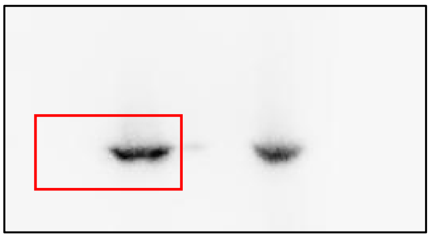

60 kDa

WCL: MARCH8

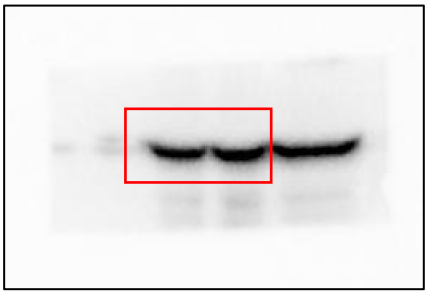

33 kDa

WCL: VANGL2

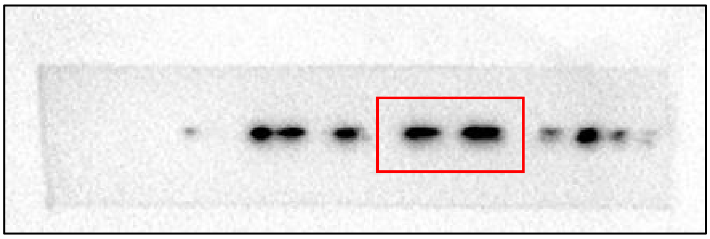

60 kDa

WCL: β-actin

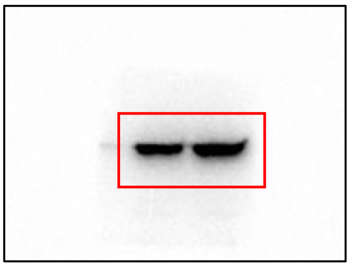

42 kDa

Figure S5A

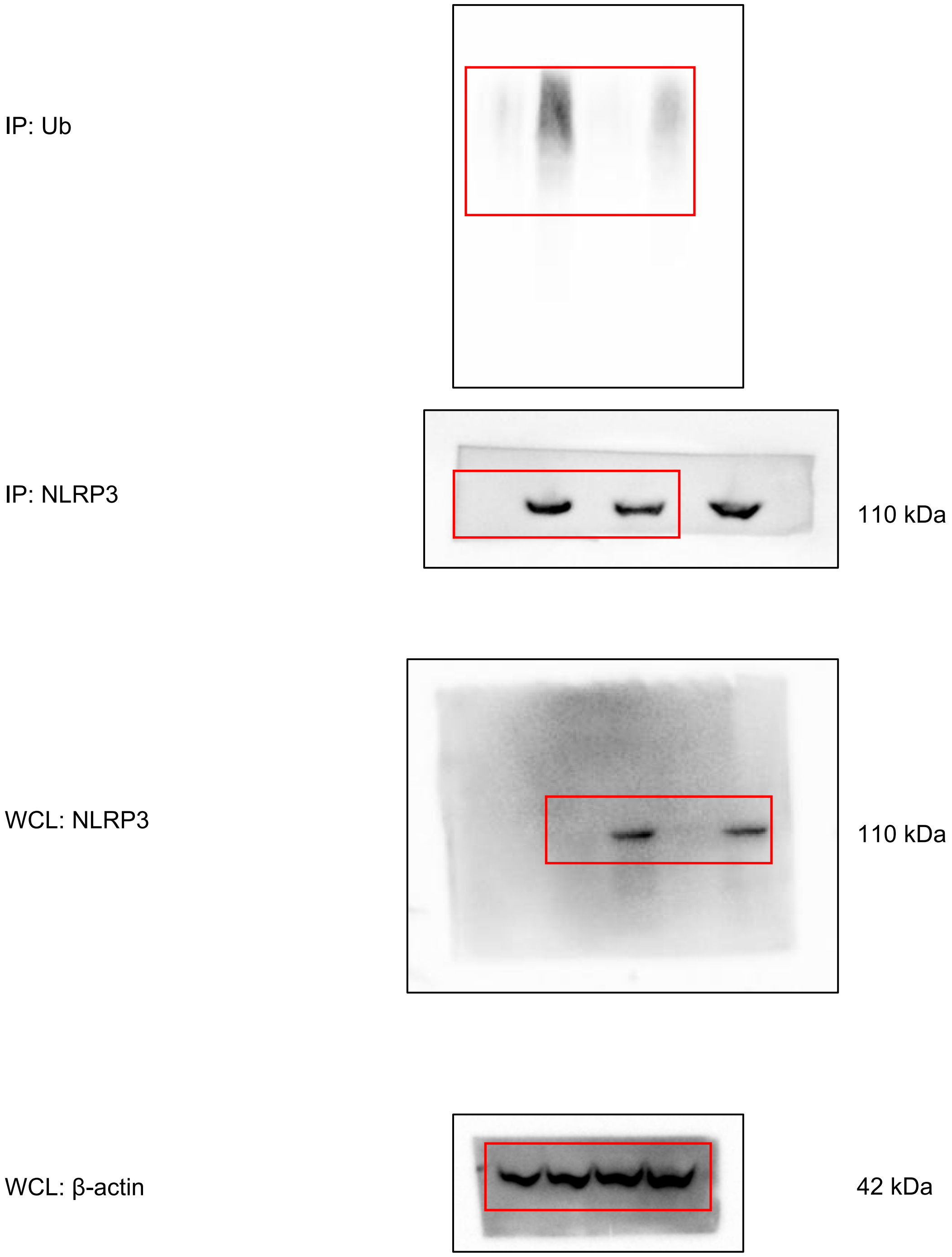

Figure S5B

IP: HA

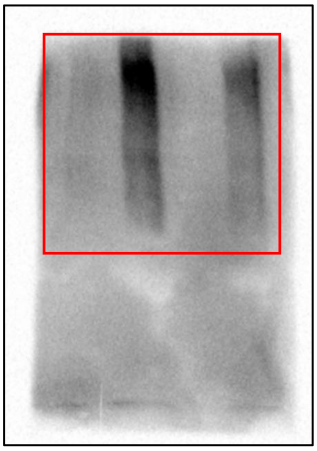

IP: Flag

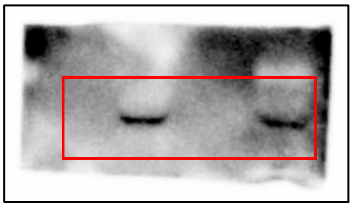

110 kDa

WCL: HA

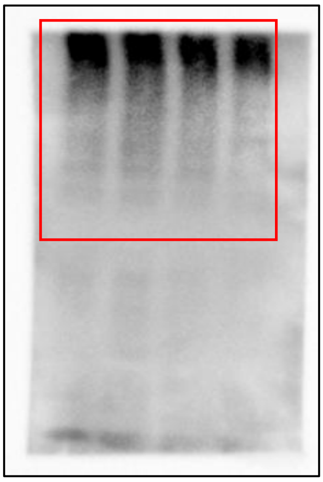

WCL: Flag

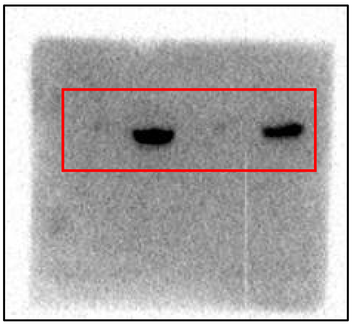

110 kDa

WCL: β-actin

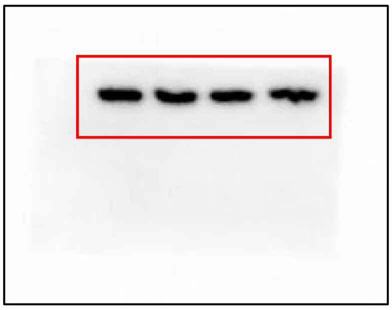

42 kDa

Figure S5C

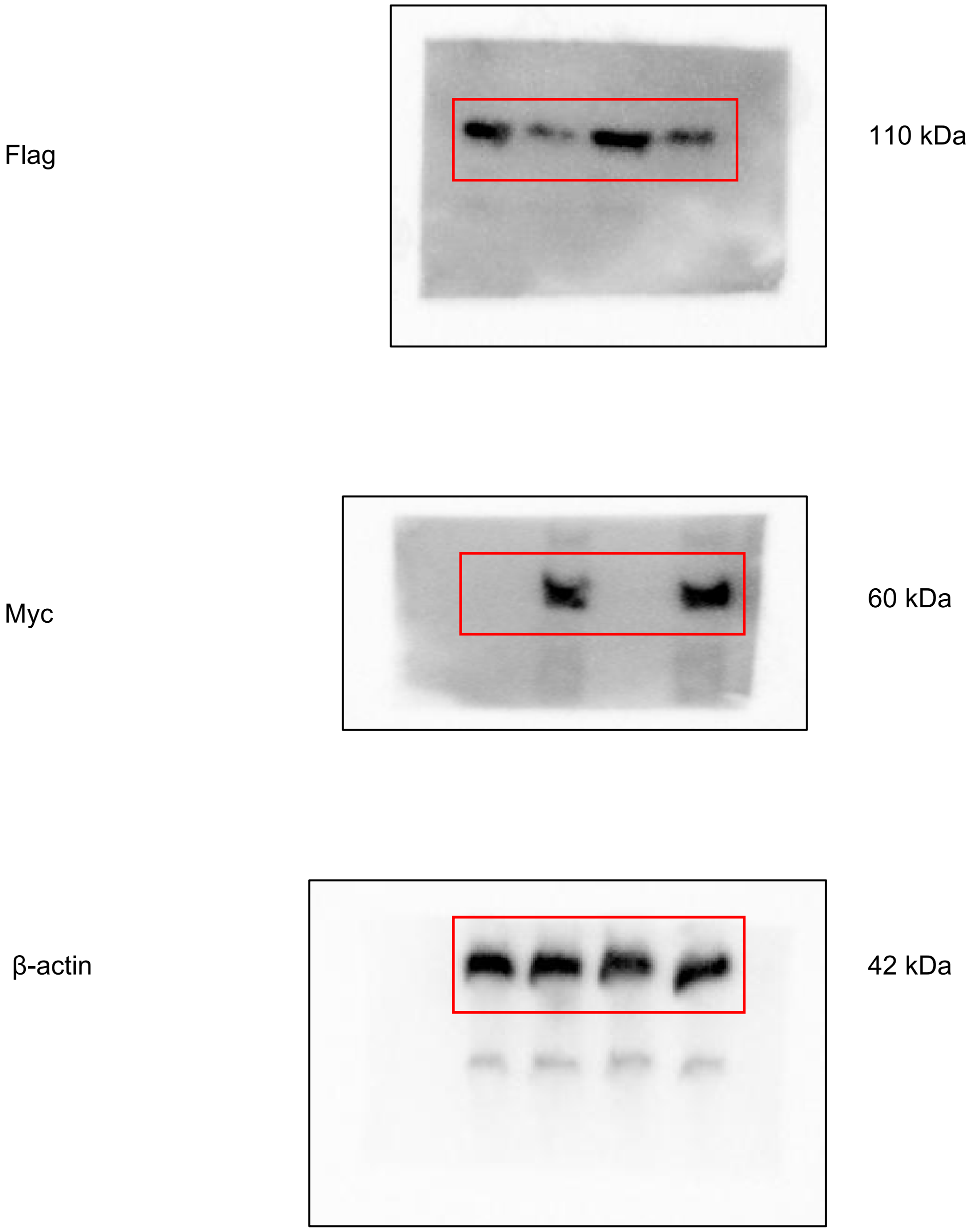

Figure S5D

Flag

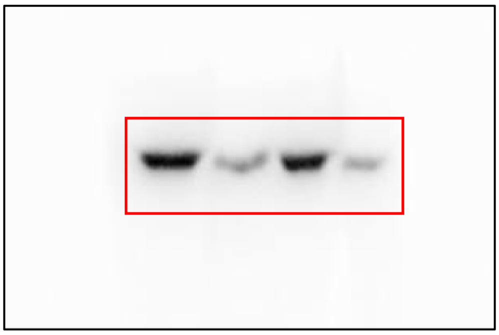

110 kDa

Myc

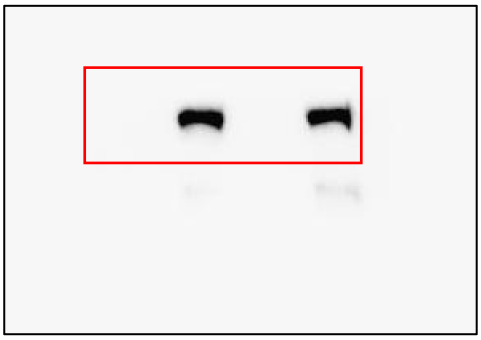

60 kDa

β-actin

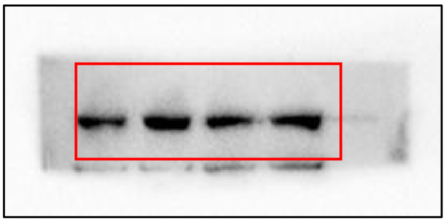

42 kDa

Figure S5E

Flag

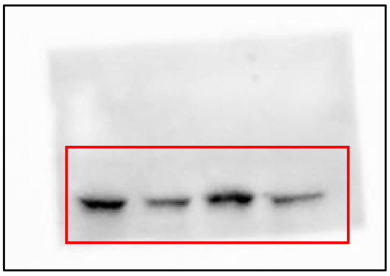

110 kDa

Myc

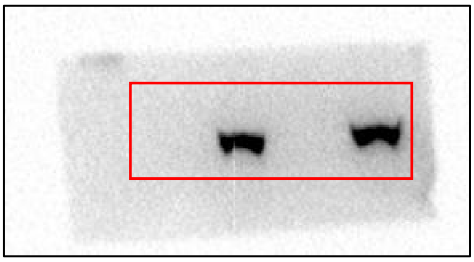

60 kDa

β-actin

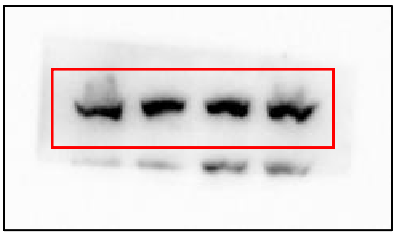

42 kDa

Figure S5F

IP HA

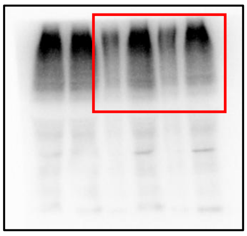

IP Flag

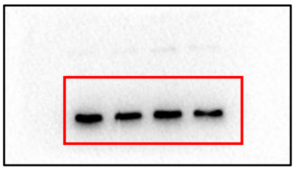

110 kDa

WCL HA

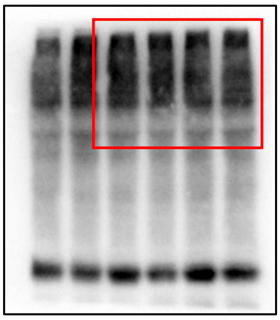

WCL Flag

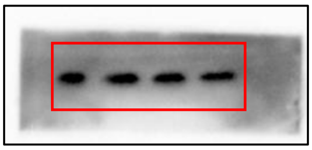

110 kDa

WCL Myc

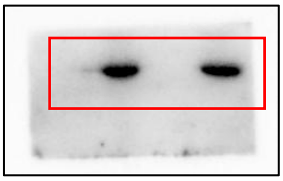

60 kDa

WCL MARCH7

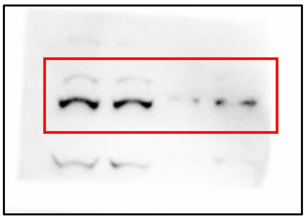

78 kDa

β-actin

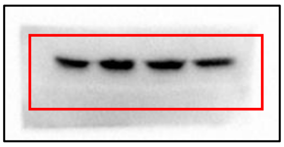

42 kDa

Figure S5G

Flag

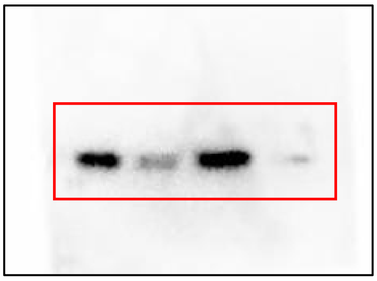

110 kDa

Myc

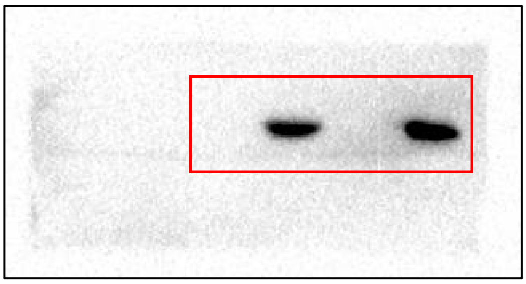

60 kDa

MARCH7

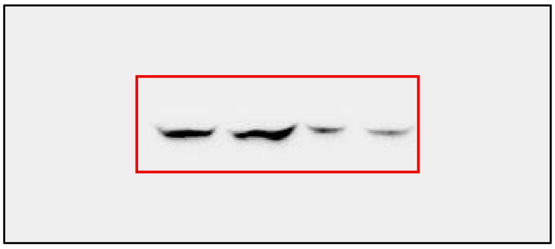

78 kDa

β-actin

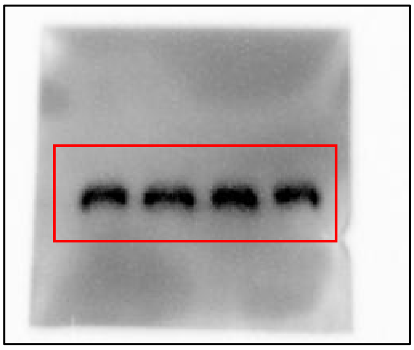

42 kDa

Figure S5H

NLRP3

110 kDa

β-actin

42 kDa

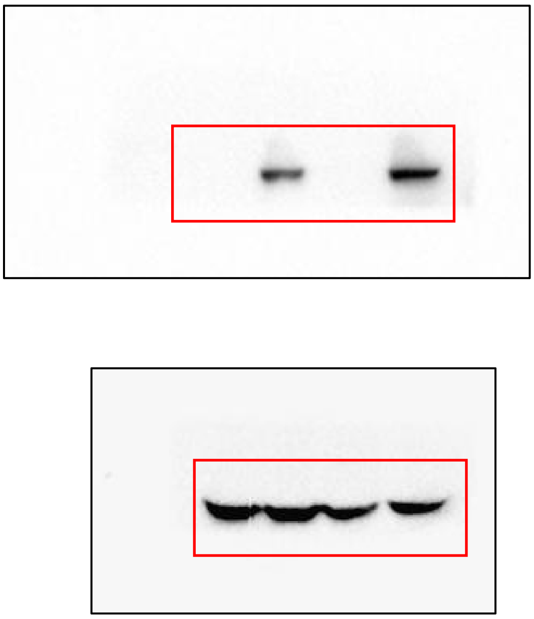

Figure 6B

IP: HA

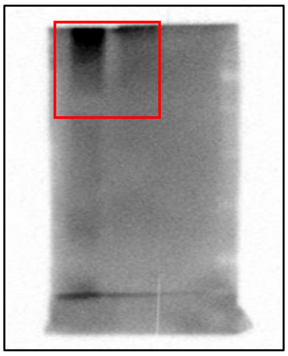

IP: Flag

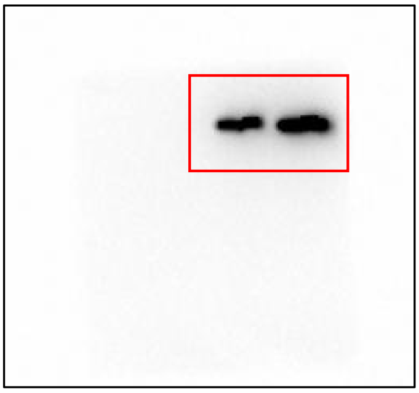

110 kDa

WCL: HA

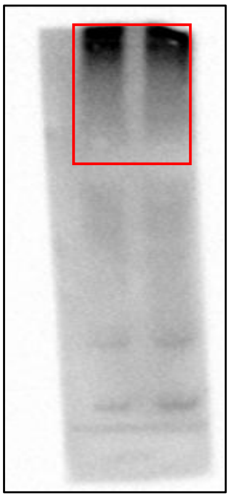

WCL: Flag

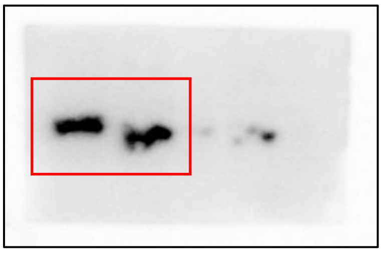

110 kDa

WCL: β-actin

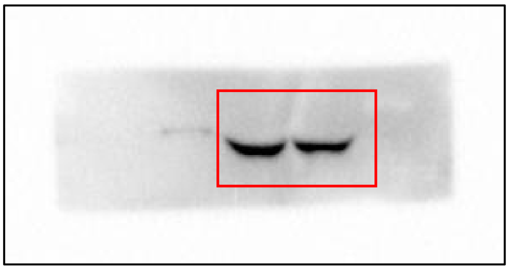

42 kDa

Figure 6C

IP: HA

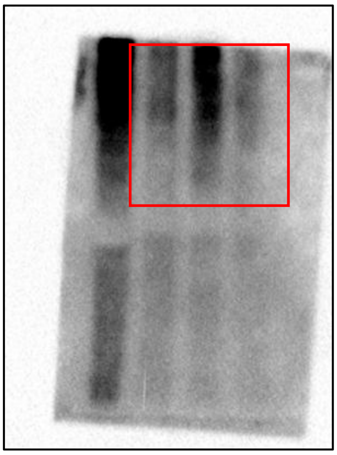

IP: Flag

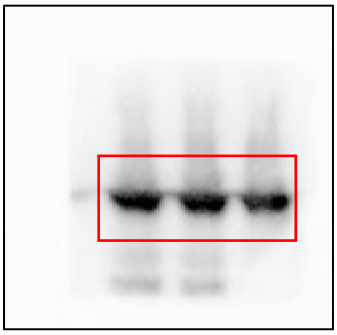

110 kDa

WCL: HA

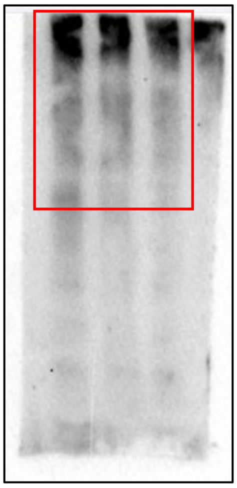

WCL: GFP

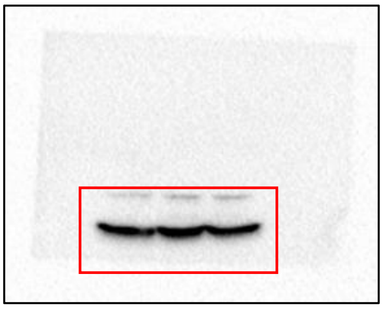

33 kDa

WCL: Flag

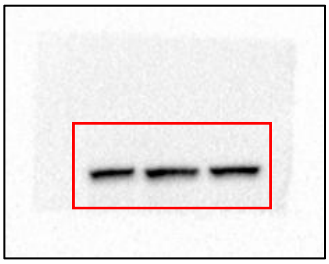

110 kDa

WCL: β-actin

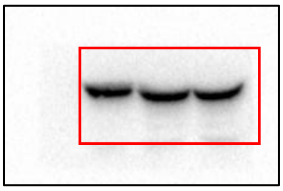

42 kDa

Figure 6D

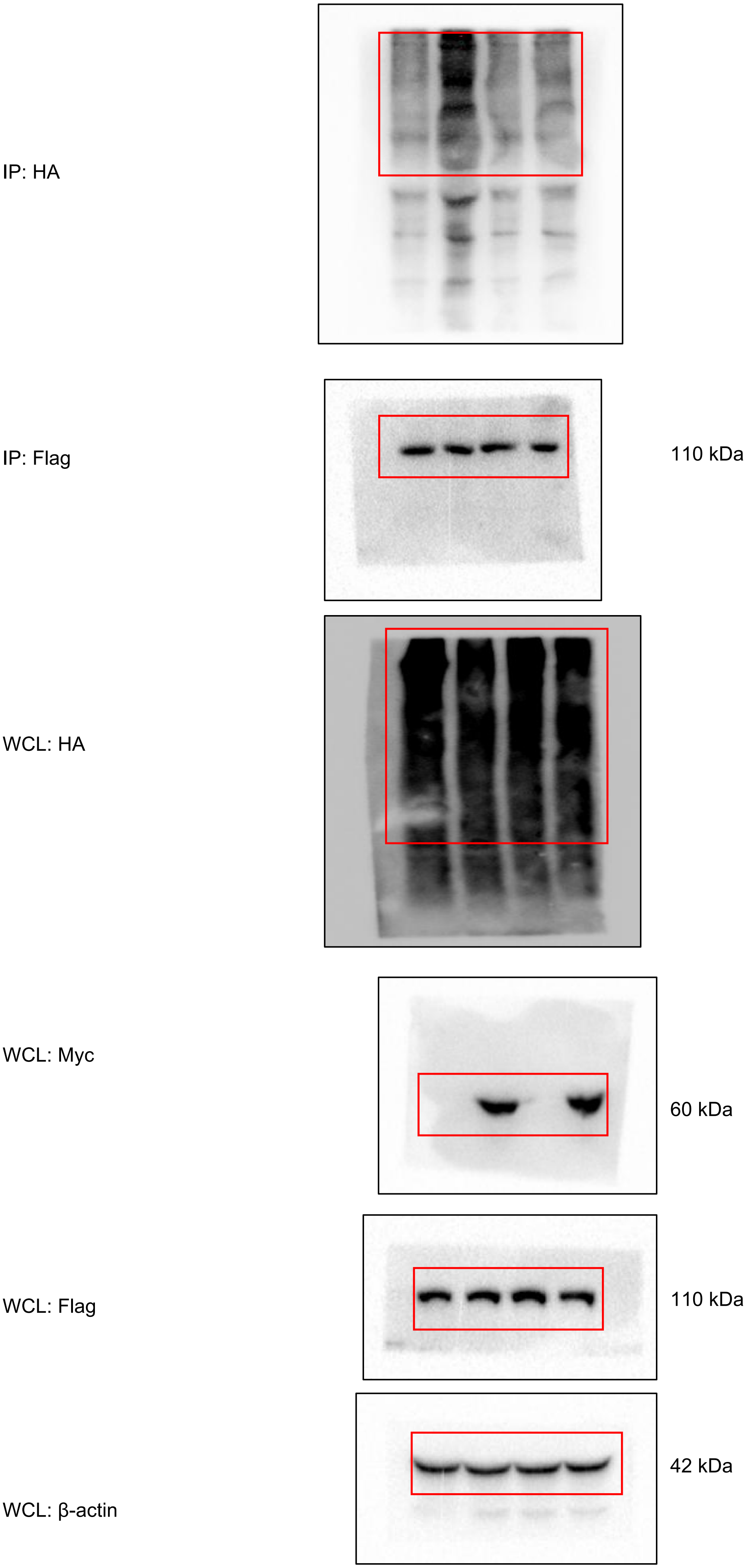
IP: HA
IP: Flag
110 kDa
WCL: HA
WCL: Myc
60 kDa
WCL: Flag
110 kDa
42 kDa
WCL: β-actin

Figure 6E

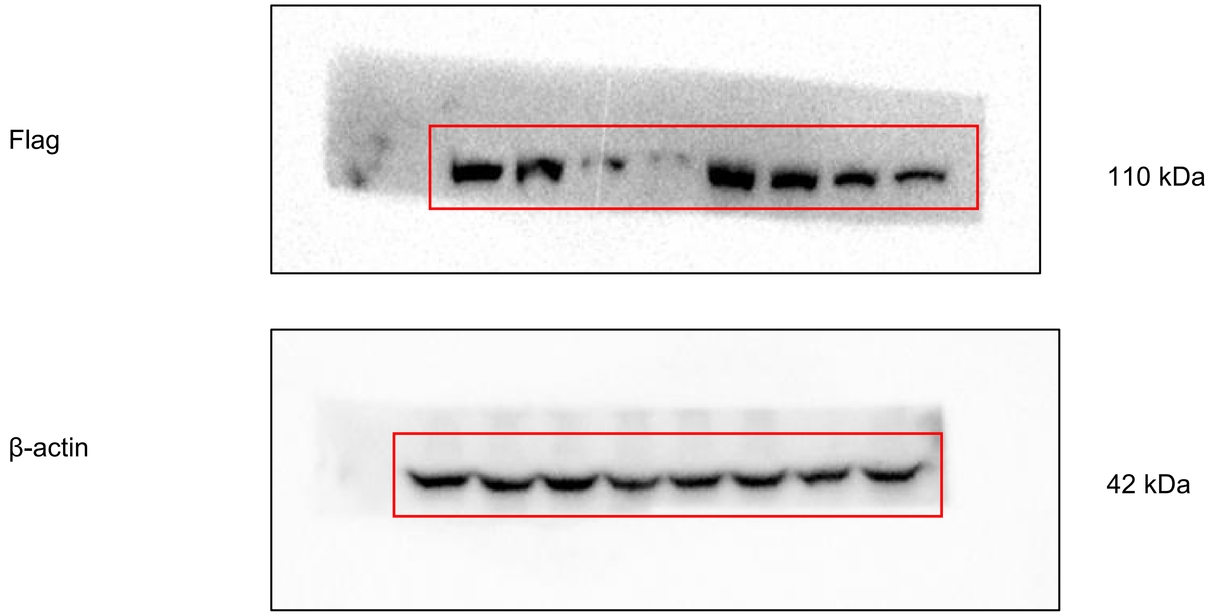

Figure 6G

Flag

GFP

β-actin

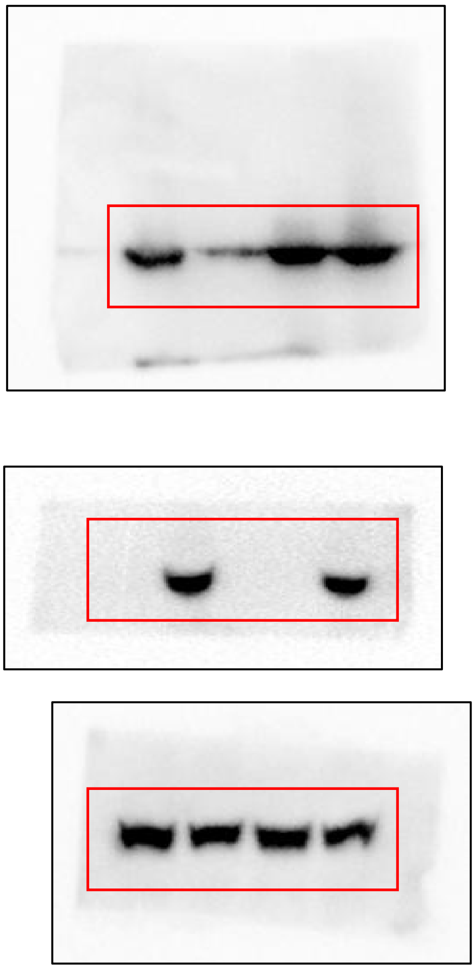

110 kDa

33 kDa

42 kDa

Figure 6H

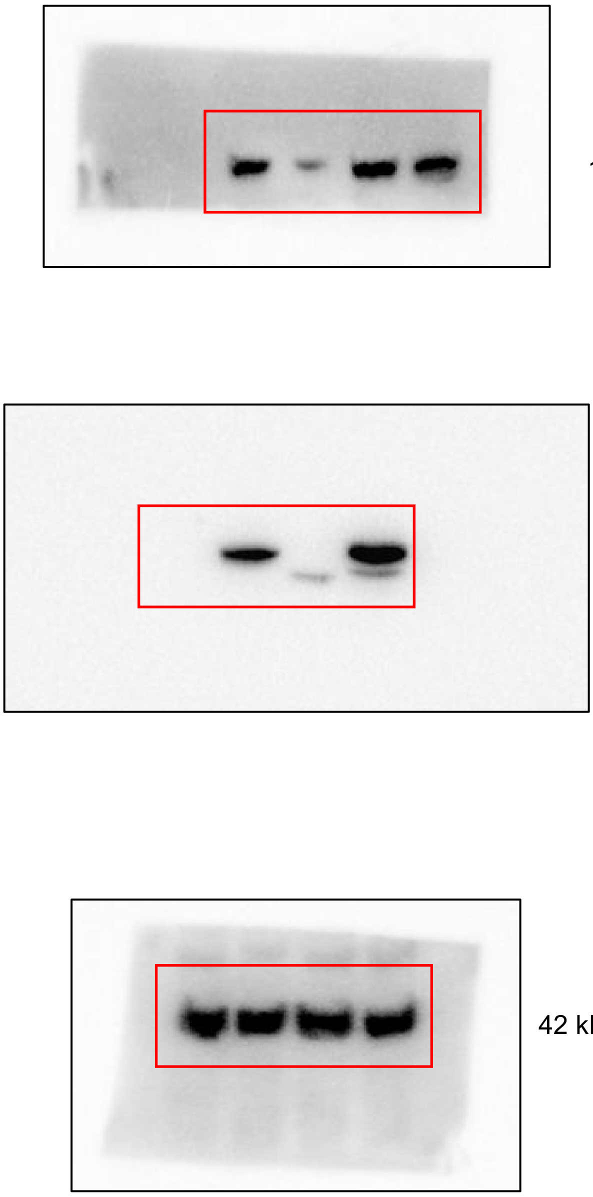

Flag 110 kDa

Myc 60 kDa

β-actin 42 kDa

Figure 6I

IP: HA

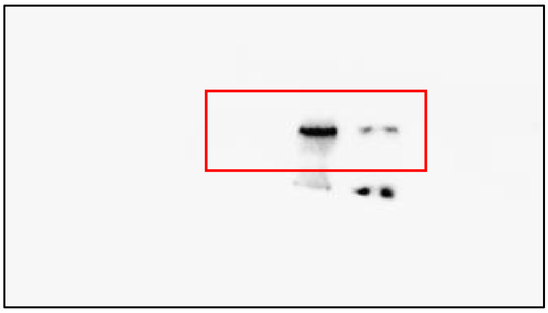

78 kDa

IP: Flag

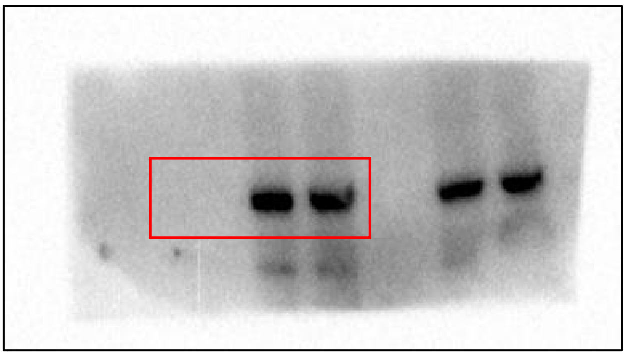

110 kDa

WCL: HA

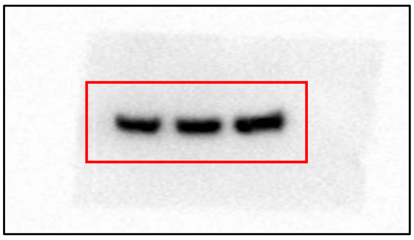

78 kDa

WCL: Flag

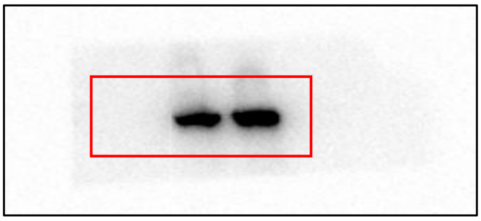

110 kDa

WCL: β-actin

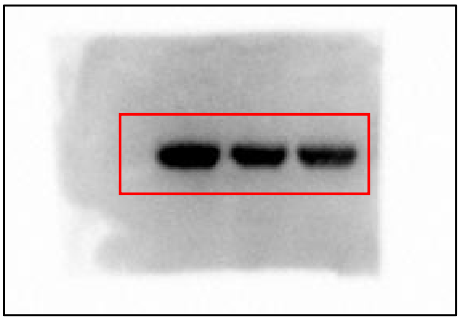

42 kDa

Figure 6J

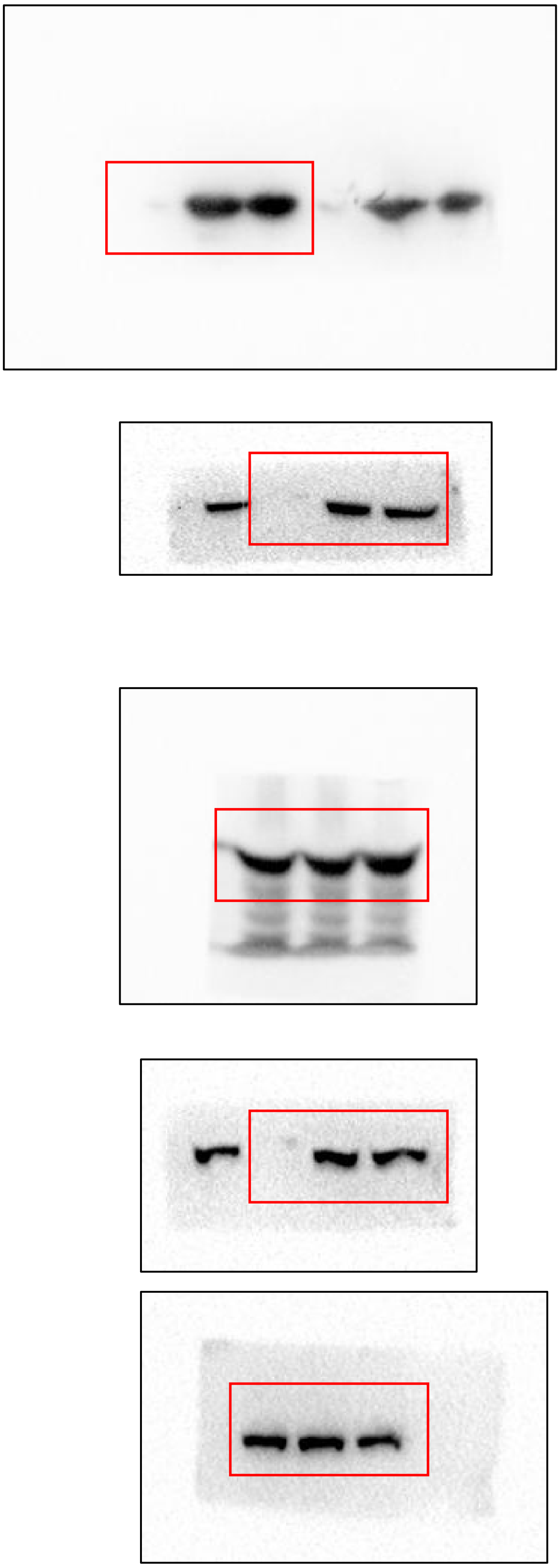

IP: HA — 60 kDa

IP: Flag — 110 kDa

WCL: HA — 60 kDa

WCL: Flag — 110 kDa

WCL: β-actin — 42 kDa

Figure S6A

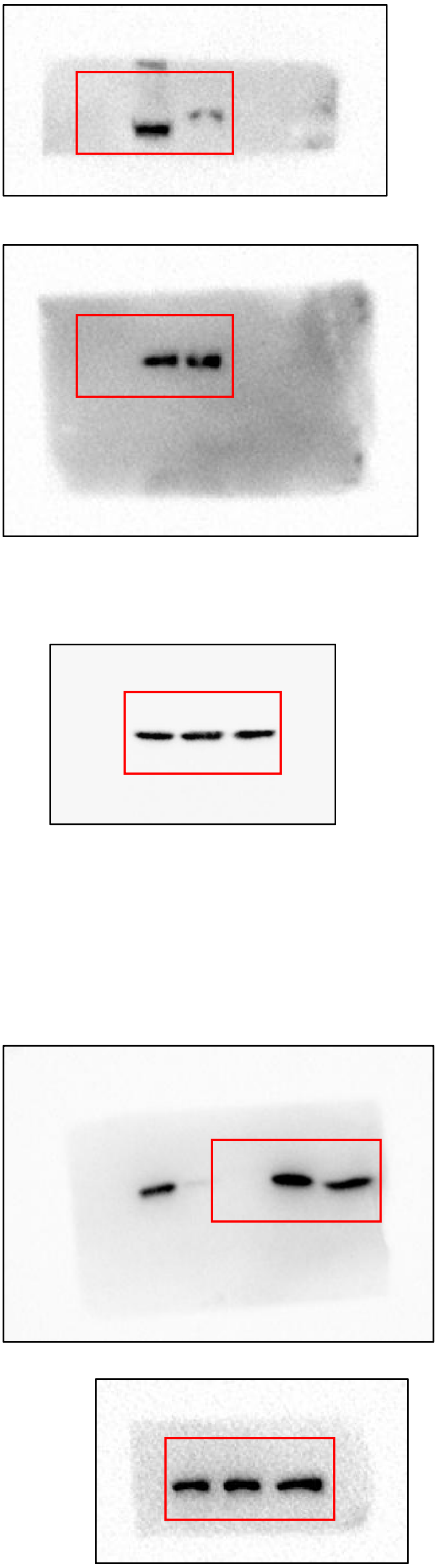

IP: GFP — 14 kDa

IP: Flag — 110 kDa

14 kDa

WCL: GFP

WCL: Flag — 110 kDa

WCL: β-actin — 42 kDa

Figure S6B

VANGL2

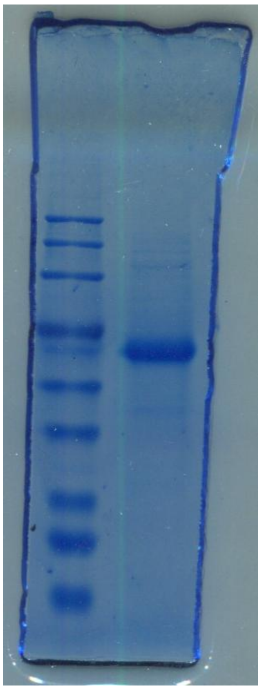

NLRP3

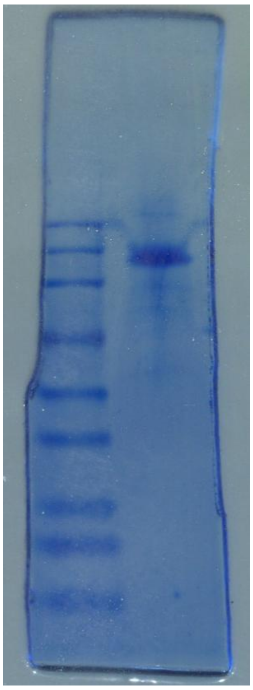

MARCH8

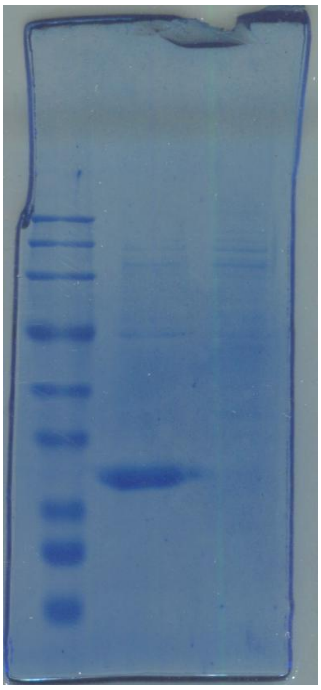

OPTN

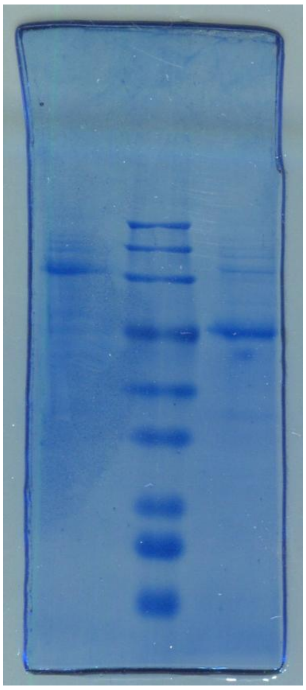

Figure S6C

IP VANGL2

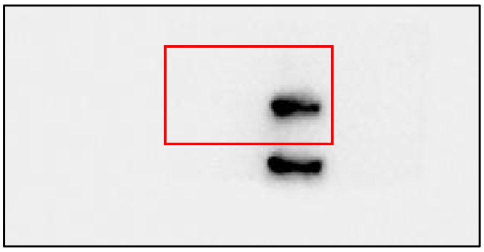

60 kDa

IP NLRP3

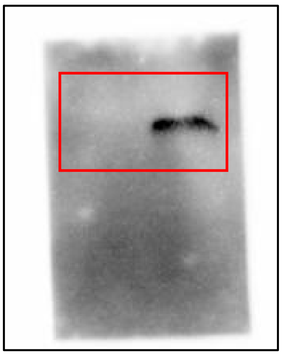

110 kDa

WCL VANGL2

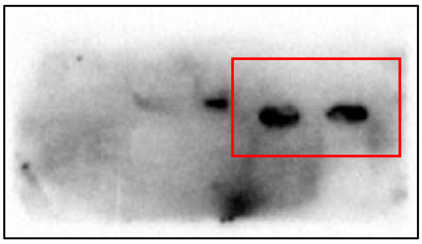

60 kDa

WCL NLRP3

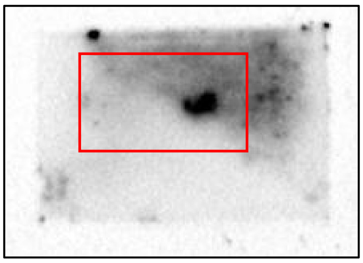

110 kDa

Figure S6D

IP MARCH8

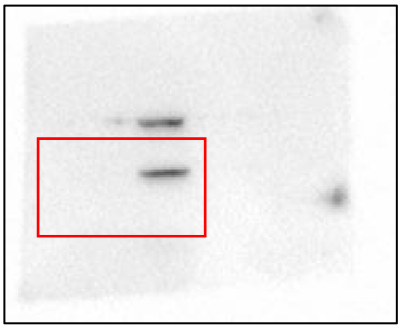

33 kDa

IP NLRP3

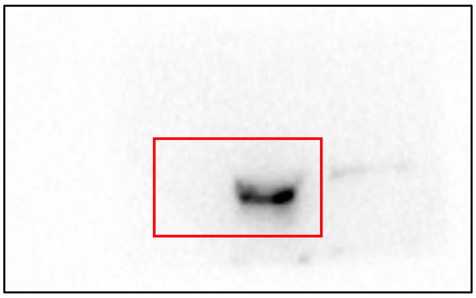

110 kDa

WCL MARCH8

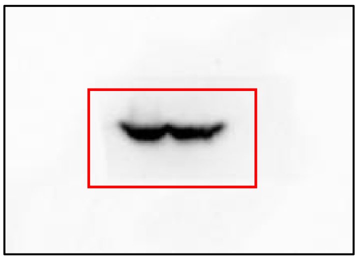

33 kDa

WCL NLRP3

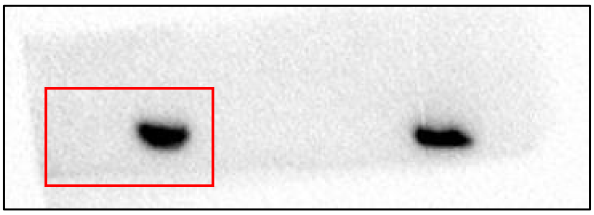

110 kDa

Figure S6E

IP OPTN

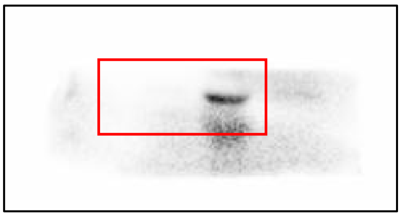

78 kDa

IP NLRP3

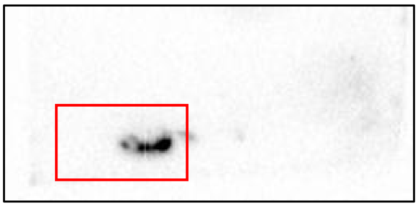

110 kDa

WCL OPTN

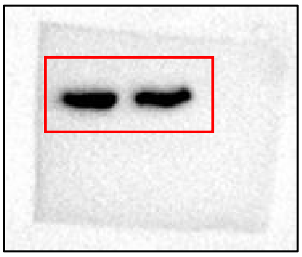

78 kDa

WCL NLRP3

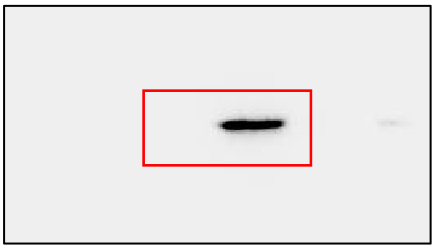

110 kDa

Figure S6F

IP MARCH8

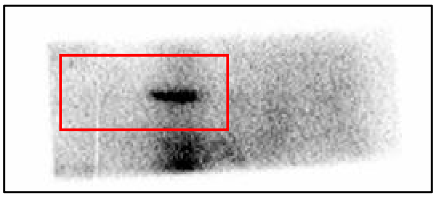

33 kDa

IP VANGL2

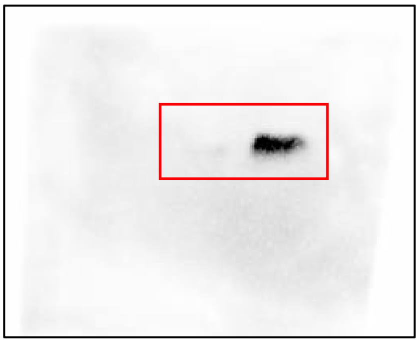

60 kDa

WCL MARCH8

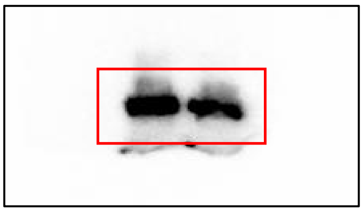

33 kDa

WCL VANGL2

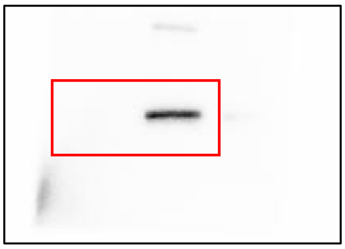

60 kDa

Figure S6G

IP OPTN

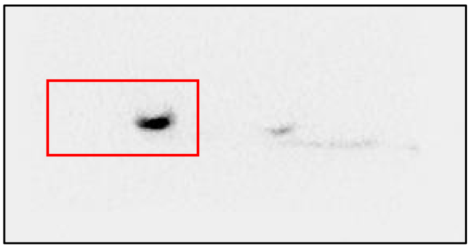

78 kDa

IP VANGL2

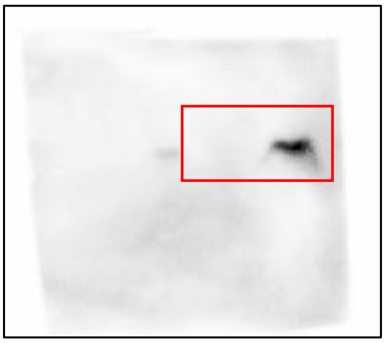

60 kDa

WCL OPTN

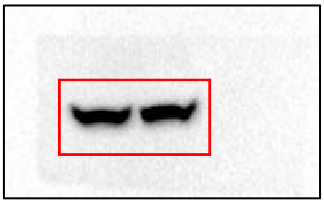

78 kDa

WCL VANGL2

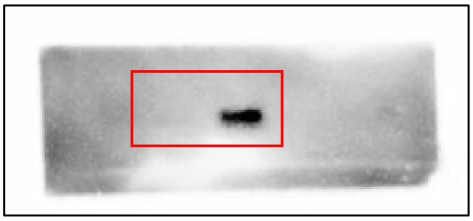

60 kDa

Figure S6H

IP MARCH8

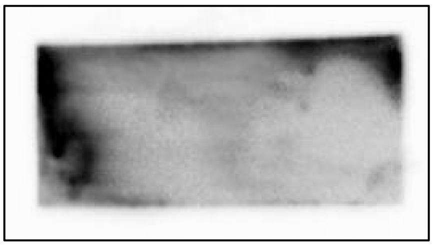

33 kDa

IP OPTN

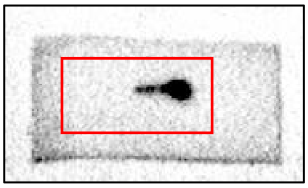

78 kDa

WCL MARCH8

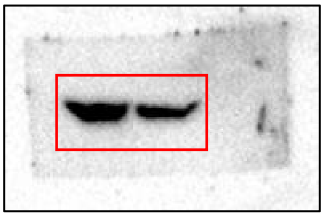

33 kDa

WCL OPTN

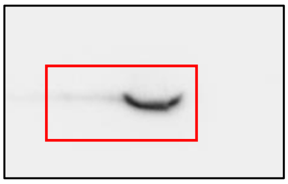

78 kDa

Figure S6I

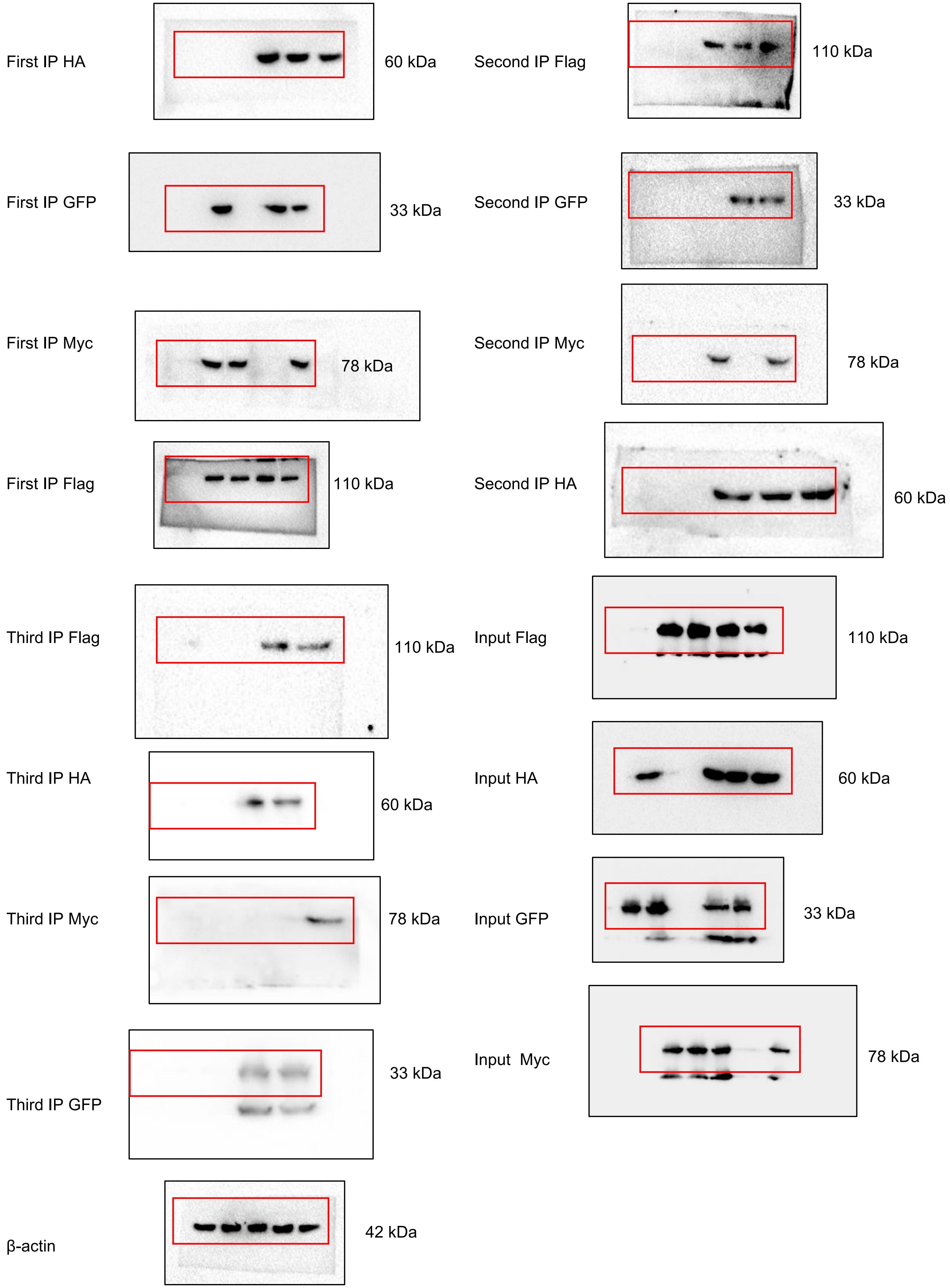
First IP HA
60 kDa
Second IP Flag
110 kDa
First IP GFP
33 kDa
Second IP GFP
33 kDa
First IP Myc
78 kDa
Second IP Myc
78 kDa
First IP Flag
110 kDa
Second IP HA
60 kDa
Third IP Flag
110 kDa
Input Flag
110 kDa
Third IP HA
60 kDa
Input HA
60 kDa
Third IP Myc
78 kDa
Input GFP
33 kDa
Input Myc
78 kDa
Third IP GFP
33 kDa
β-actin
42 kDa

Figure 7J

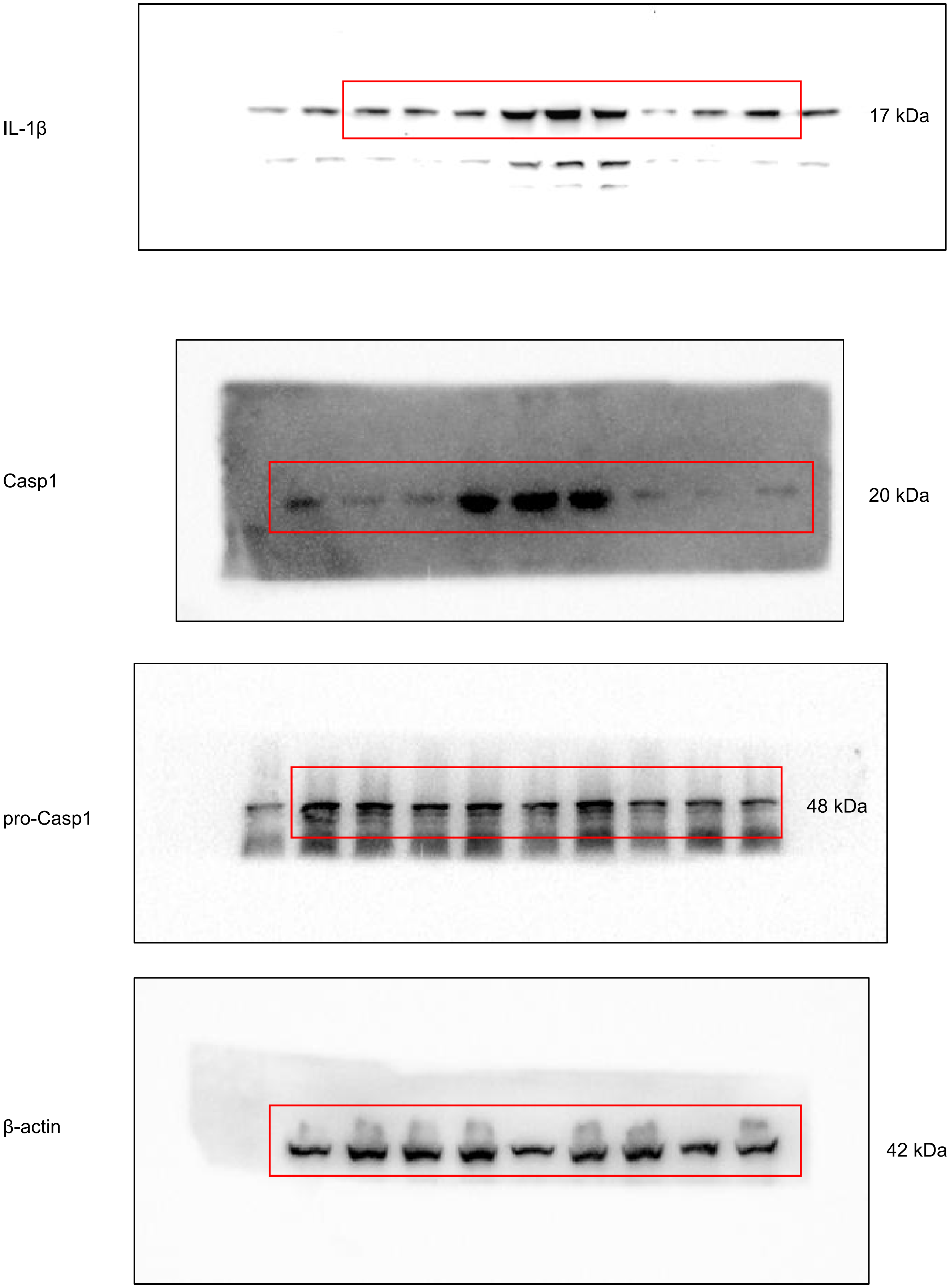

Figure 8G

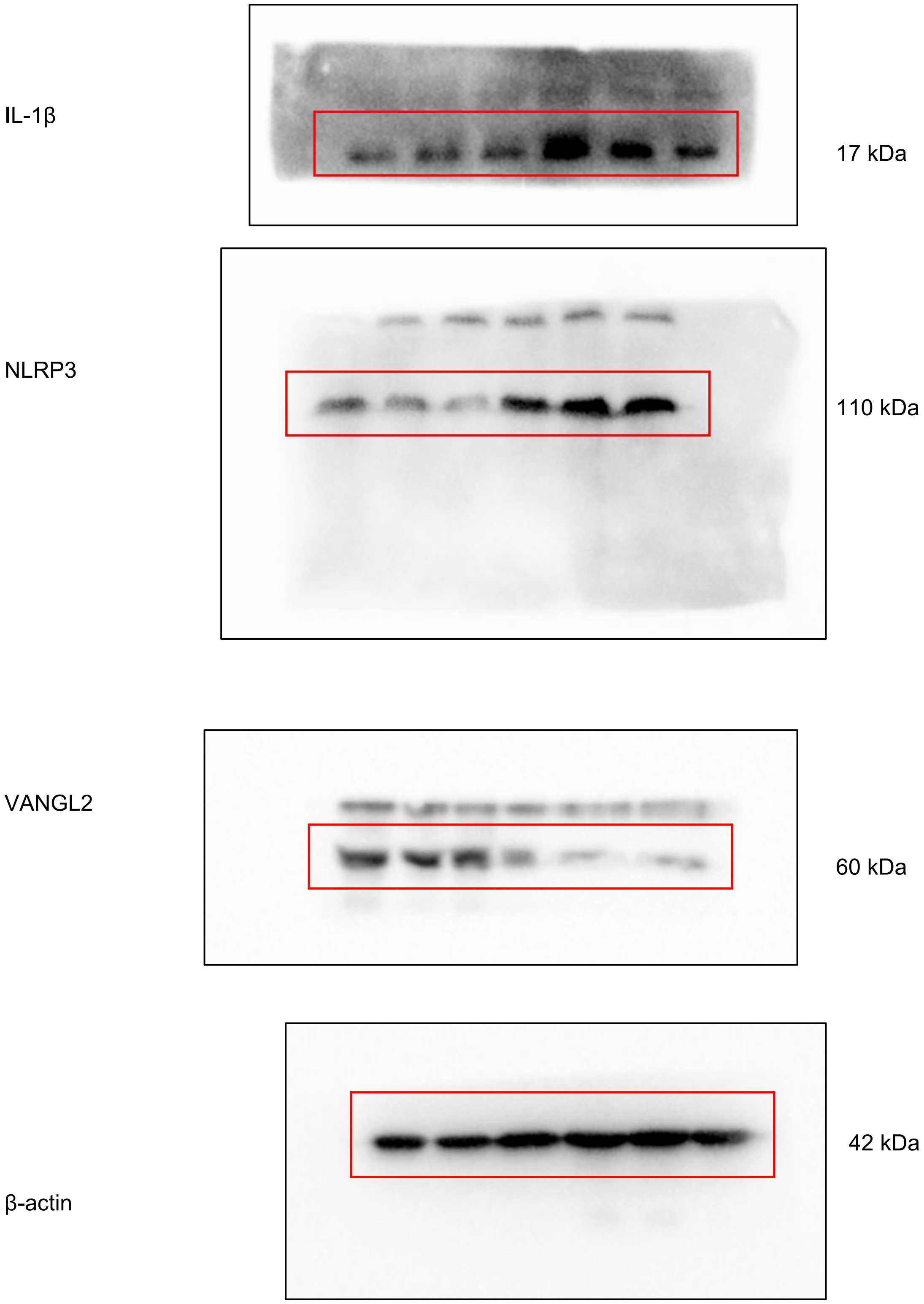

Figure 8I

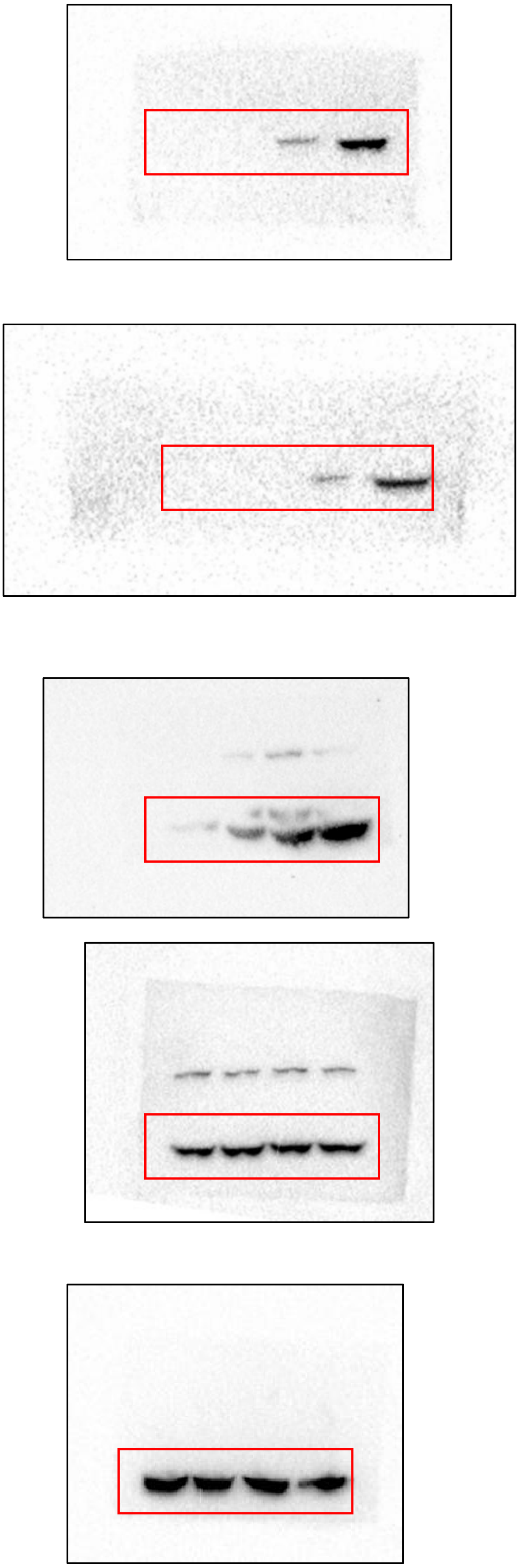

IL-1β — 17 kDa

Casp1 — 20 kDa

NLRP3 — 110 kDa

pro-Casp1 — 48 kDa

β-actin — 42 kDa

Figure 8K

IL-1β

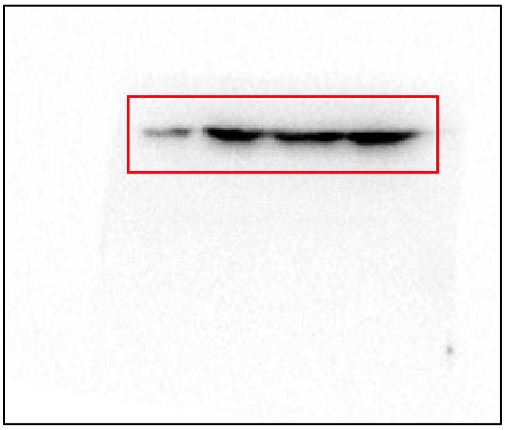

17 kDa

Casp1

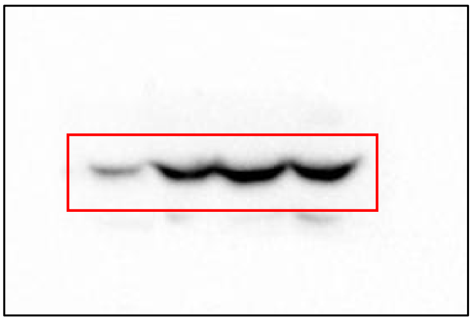

20 kDa

NLRP3

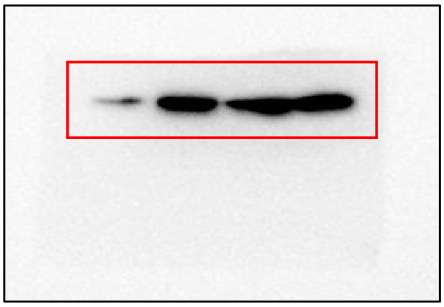

110 kDa

pro-Casp1

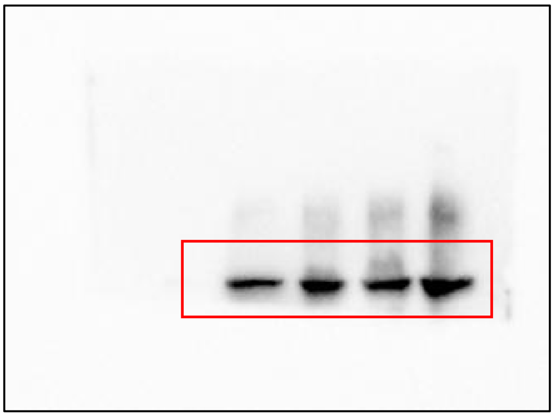

48 kDa

β-actin

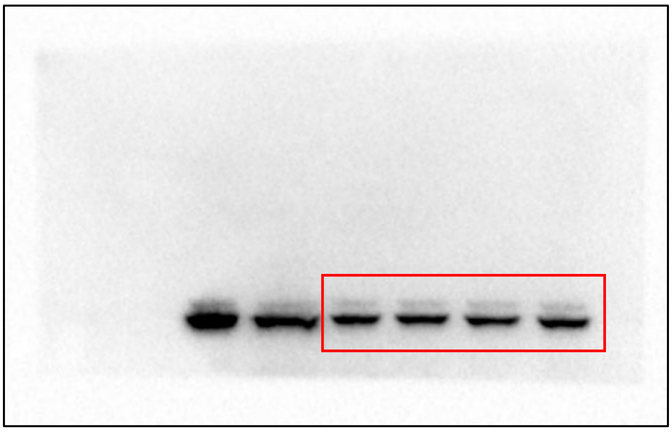

42 kDa

Figure 8M

IL-1β

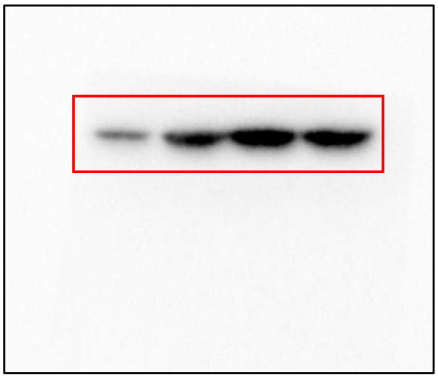

17 kDa

Casp1

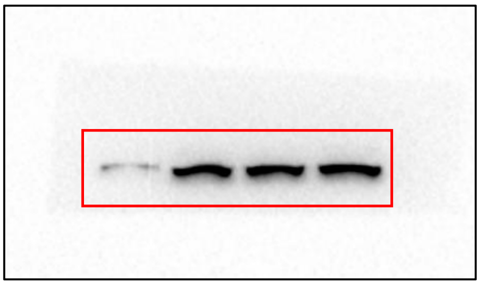

20 kDa

NLRP3

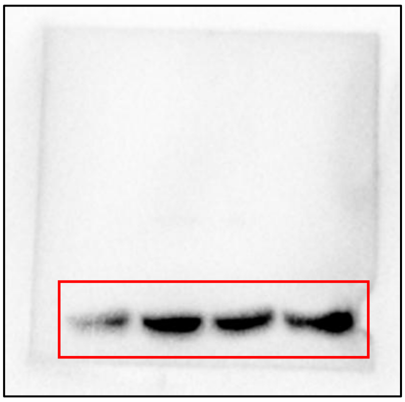

110 kDa

pro-Casp1

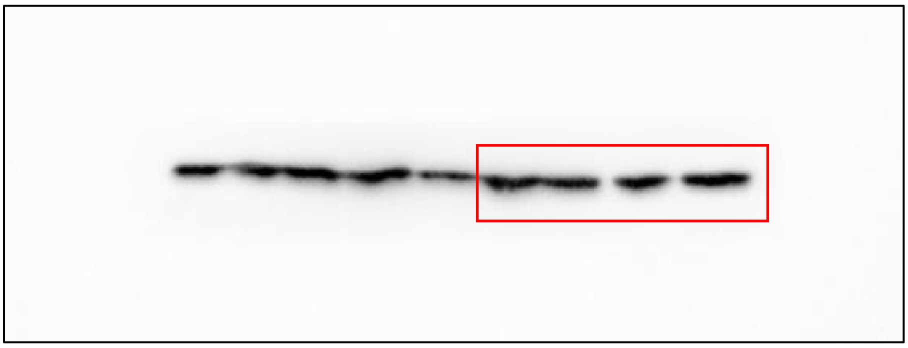

48 kDa

β-actin

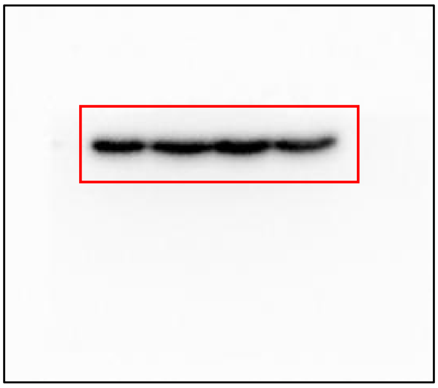

42 kDa
